# Exploring Genetic Factors Involved in Huntington Disease Age of Onset: *E2F2* as a New Potential Modifier Gene

**DOI:** 10.1371/journal.pone.0131573

**Published:** 2015-07-06

**Authors:** Leire Valcárcel-Ocete, Gorka Alkorta-Aranburu, Mikel Iriondo, Asier Fullaondo, María García-Barcina, José Manuel Fernández-García, Elena Lezcano-García, José María Losada-Domingo, Javier Ruiz-Ojeda, Amaia Álvarez de Arcaya, José María Pérez-Ramos, Raymund A. C. Roos, Jørgen E. Nielsen, Carsten Saft, REGISTRY investigators of the European Huntington's Disease Network, Ana M. Zubiaga, Ana Aguirre

**Affiliations:** 1 Department of Genetics, Physical Anthropology and Animal Physiology, University of the Basque Country (UPV/EHU), Leioa, Spain; 2 Department of Human Genetics, University of Chicago, Chicago, United States of America; 3 Genetics Unit, Basurto University Hospital, Bilbao, Spain; 4 Neurology Service, Basurto University Hospital, Bilbao, Spain; 5 Department of Neurology, Cruces University Hospital, Barakaldo, Spain; 6 Department of Neurology, Galdakao-Usansolo Hospital, Galdakao, Spain; 7 Department of Neurology, Alava University Hospital, Txagorritxu, Vitoria-Gasteiz, Spain; 8 Department of Neurology, Alava University Hospital, Santiago Apóstol, Vitoria-Gasteiz, Spain; 9 Department of Neurology, Leiden University Medical Centre (LUMC), Leiden, The Netherlands; 10 Danish Dementia Research Centre, Neurogenetics Clinic, University Hospital of Copenhagen- Rigshospitalet, Copenhagen, Denmark; 11 Huntington-Zentrum (NRW) Bochum, St. Josef-Hospital, Bochum, Germany; Inserm U837, FRANCE

## Abstract

Age of onset (AO) of Huntington disease (HD) is mainly determined by the length of the CAG repeat expansion (CAGexp) in exon 1 of the *HTT* gene. Additional genetic variation has been suggested to contribute to AO, although the mechanism by which it could affect AO is presently unknown. The aim of this study is to explore the contribution of candidate genetic factors to HD AO in order to gain insight into the pathogenic mechanisms underlying this disorder. For that purpose, two AO definitions were used: the earliest age with unequivocal signs of HD (earliest AO or eAO), and the first motor symptoms age (motor AO or mAO). Multiple linear regression analyses were performed between genetic variation within 20 candidate genes and eAO or mAO, using DNA and clinical information of 253 HD patients from REGISTRY project. Gene expression analyses were carried out by RT-qPCR with an independent sample of 35 HD patients from Basque Country Hospitals. We found suggestive association signals between HD eAO and/or mAO and genetic variation within the *E2F2*, *ATF7IP*, *GRIN2A*, *GRIN2B*, *LINC01559*, *HIP1* and *GRIK2* genes. Among them, the most significant was the association between eAO and rs2742976, mapping to the promoter region of *E2F2* transcription factor. Furthermore, rs2742976 T allele patient carriers exhibited significantly lower lymphocyte *E2F2* gene expression, suggesting a possible implication of E2F2-dependent transcriptional activity in HD pathogenesis. Thus, *E2F2* emerges as a new potential HD AO modifier factor.

## Introduction

Huntington’s disease (HD) (OMIM 143100) is a neurodegenerative disorder characterized by movement abnormalities (chorea, hypokinesia), cognitive decline and psychiatric disturbances, which most often become noticeable between the ages of 35 and 50 [1]. HD is caused by an expanded CAG trinucleotide repeat (>39 CAGs, CAGexp) in exon 1 of the *HTT* gene. Expanded alleles result in an elongated polyglutamine tract in the Huntingtin protein (HTT), which leads to defects in intracellular trafficking and signalling pathways, as well as in nervous system development during embryogenesis [2]. Assessment of Huntington disease age of onset (AO), that is, the point in time when a carrier of the expanded allele develops unequivocal HD signs [3], remains to be clearly defined at the phenotypic level, as different criteria are being used to estimate AO. Involuntary movements, such as chorea, are the most distinctive HD symptom that can be established with reliability, and their debut commonly defines HD age of onset [4]. However, motor symptoms are often preceded by cognitive and/or psychiatric decline [5]. Consequently, some authors have defined AO as the age at which the first possible symptom is detected [6–8].

Multiple studies have shown an inverse correlation between HD age of onset and CAGexp. However, this correlation only accounts for a fraction of the total AO variability, which ranges between 42% and 73% [6–10]. This range may be attributed, among others, to the characteristics of the studied population, the HD phenotypes considered (i.e. inclusion or not of juvenile HD cases) and/or the criteria used to define the age of onset [11,12].

AO variability not explained by CAGexp shows strong heritability (40% to 56%) [12,13], suggesting the contribution of additional genetic factors in determining AO in HD. Indeed, genetic variation influencing AO has been reported previously. Association signals have been replicated for some of those genes (e.g. *GRIK2* [10,14,15], *GRIN2A* and *GRIN2B* [16–18], *TCERG1* [10,19], *HAP1* [20,21], *ADORA2A* [8,21], *PPARGC1A* [22,23], *ATG7* [24,25]), but not for others (e.g. *TP53*, *UCHL1*, *DFFB*, *APOE* and *MTHFR* [26], *GSTO1* [27], *ASK1* or *MAP3K5* and *MAP2K6* [28]).

Most polymorphisms exhibiting association signals with HD AO are located in non-coding regions. They may affect mRNA splicing or transcriptional regulation [18], for example, or they may represent markers in linkage disequilibrium (*LD*) with AO modifiers. However, none of the associated SNPs has been functionally validated to date, and little is known about the mechanisms by which these polymorphisms may affect AO in HD.

The aim of this study is to improve our understanding of the contribution of AO modifiers to HD pathogenesis. To this end, we have studied the effect of genetic variation within 20 candidate genes on HD age of onset. We replicate some of the previously reported association signals. Moreover, we identify genetic variation in the *E2F2* promoter region that associates with HD AO and *E2F2* gene expression, suggesting a potential molecular explanation for this association.

## Material and Methods

### Patients and phenotype data

The European Huntington’s Disease Network (EHDN) provided DNA samples and clinical data from 284 individuals forming part of the REGISTRY project. The clinical data supplied included age (from 18 to 82 years old), sex (146 men and 138 women), self-assigned ethnicity (99.3% had European origin), mutated CAG repeat number (CAGexp), symptoms and AO information (S1 Table). Additionally, the EHDN obtained a written informed consent, in compliance with the Declaration of Helsinki, Internal Conference of Harmonisation-Good Clinical Practice (ICH-GCP), and local regulations, from each participant. AO was estimated by the raters, following the Unified Huntington's Disease Rating Scale (UHDRS’99) and the Hamilton Depression Rating Scale (HDRS). Two definitions of AO provided by EHDN were used: the earliest AO (eAO), i.e., the age of the patient at which the first unequivocal signs of HD (motor, cognitive or psychiatric) appeared, and the motor AO (mAO) i.e., the age at which the first motor symptoms appeared. The eAO data was available for all individuals in the sample; the mAO data was available for 196 individuals.

Only European origin individuals with adult-onset HD (> 20 years old) were considered in the study, and single outliers with CAGexp repeat number outside the 40–53 range were excluded [29,30]. Individuals with psychiatric symptoms as their first HD manifestation and with family history of mental disease were excluded to avoid possible effects of hereditary psychiatric disorders. The final number of selected patients in the eAO and mAO groups was 255 and 180, respectively. The analyzed individuals were from Germany (N = 62), Italy (N = 40), United Kingdom (N = 36), Poland (N = 31), The Netherlands (N = 25), Spain (N = 17), Denmark (N = 11), Norway (N = 8), Austria (N = 7), Portugal (N = 6), Finland (N = 5), Belgium (N = 4), Czech Republic (N = 2) and Sweden (N = 1).

For gene expression analyses, blood samples from 35 European origin individuals belonging to 27 HD families from hospitals of the Basque Country were collected after clinical and molecular HD diagnosis. Most of patient’s ancestors come from Spanish regions other than the Basque Country, and two have Basque ancestry (their four grandparents were Basque). This sample was composed of 20 men and 15 women with ages ranging between 28 and 83 years old. Details of each sample are shown in S2 Table. Written informed consent was obtained from all patients and the study was approved by the Ethics Committee for Clinical Research of Euskadi and by the Ethics Committee for Research and Teaching of the University of the Basque Country (UPV/EHU).

### Genes, SNPs and Genotypes

Twenty candidate genes were targeted. Seventeen genes were selected based on the following three criteria: (a) reported association with HD AO in previous studies (*BDNF* [31], *DFFB* [7], *GRIK2* [10,14,15], *GRIN2A* and *GRIN2B* [16–18], *HAP1* [20,21], *PPARGC1* [22,23] and *TCERG1* [10,19]); (b) participation of the gene product in a pathway or process altered in HD (*CASP6* [32,33], *CASP8* [34], *E2F1* [35] and *E2F2* [36]); (c) direct interaction of the gene product with HTT (*CDK5* [37,38], *HIP1* [39], *SGK1* [40,41], *SIRT1* [42] and *SP1* [43]). Functionally, these genes are involved in processes such as apoptosis (*DFFB* [44,45], *CASP8* [34,46], *CASP6* [32,33] and *HIP1* [46,47]), neuronal survival (*BDNF* [48,49], *SGK1* [40,41] and *CDK5* [37,38]), glutamatergic synapse/transmission (*GRIK2*, *GRIN2A* and *GRIN2B* [50,51]), transcriptional control/cell proliferation (*E2F1* [35], *E2F2* [36], *SP1* [52], *PPARGC1* [53], *TCERG1* [19] and *SIRT1* [54,55]) or intracellular trafficking (*HAP1* [56]). The remaining three genes were selected on the basis of their physical proximity to suggestive signals observed in our preliminary studies: *CCL26* (mapping close to *HIP1*), *LINC01559* and *ATF7IP* (both mapping close to *GRIN2B*).

Using HapMap CEU population data, a total of 117 SNPs not in *LD* (D’< 0.7) were selected in the 20 genes, all with intermediate allele frequencies and located within exonic, intronic or regulatory regions to ensure allele detection and to aim a comprehensive coverage of the majority of common variation in each gene. *LD* information used in the SNP selection process was obtained from the Centre for Genomics and Global Health (http://www.gmap.net/marker/). Some of the selected SNPs had been studied in previous association analyses between genetic polymorphisms and AO in HD. Details about each genotyped SNP are shown in S3 Table.

SNPs were genotyped using SNPlex genotyping system (Life Technologies) and these genotypes are shown in S4 Table. Two individuals were excluded from the analyses due to call-rates < 50%. Twenty-seven SNP genotypes failed or showed < 90% call-rate, and two SNPs were not in Hardy-Weinberg equilibrium, and were therefore excluded (S3 Table). Altogether, a total of 88 SNP genotype data were used for genetic association analyses on 253 individuals with eAO data and on 178 individuals with mAO data.

### Association analysis

T–test and Mann-Whitney *U* test (SPSS Ver.17.0, SPSS Inc) were used to compare the CAGexp and AO mean and median, respectively, of Southern European (Portugal, Spain and Italy) and the rest of the populations (called Northern Europeans).

Population stratification was examined with F-statistics according to the unbiased fixation index (F_ST_) proposed by Weir and Cockerham (1984) [57] using the FSTATv2.9.3 software [58]. The standard deviations of F-statistics and the confidence intervals were calculated with bootstrapping (100,000 permutations) over loci.

The correlation between CAGexp and the logarithm (log) of eAO or mAO, and the contribution of CAGexp to the variability of each AO were estimated by correlation analysis and simple linear regression analysis (SPSS), respectively. The association between log AO and each SNP was estimated by multiple linear regression analysis (SPSS) according to dominant, recessive and additive model based on the minor allele, and corrected using Bonferroni procedure. In all association analyses, log AO was used as dependent variable and CAGexp as independent variable. *LD* information from the 253 patients was obtained using Haploview v4.0 [59].

### Reverse transcription quantitative PCR (RT-qPCR) analysis

Total RNA was obtained from peripheral blood mononuclear cells of patients from 5 hospitals of the Basque Country using TRIzol reagent (Life Technologies), following the manufacturer’s instructions. The RNA was treated with DNase, purified using RNeasy kit (Qiagen), quantified with NanoDrop ND-1000 and examined for RNA integrity (RIN>7) with 2100 Bioanalyzer (Agilent Technologies).

*E2F2* gene expression was analyzed with both SYBR Green-based and Taqman-based assays (Life Technologies). The cDNA was synthesized using 2 μg (for SYBR Green-based assays) or 500ng (for Taqman-based assays) of total RNA using High-capacity cDNA Reverse transcription kit (Life Technologies). For SYBR Green-based assays, forward (5´ACG TGC TGG AAG GCA TCC 3´) and reverse (5´GCT CCG TGT TCA TCA GCT CC 3´) primers, located in exons 3 and 4 of *E2F2*, respectively, were used. For Taqman-based assays, Hs00918089_m1 probe, which hybridizes with the 3–4 exon boundary of the *E2F2* gene, was used. The reference genes for normalization were selected according to their reported stability in leukocytes [60]. For SYBR Green assays, *GAPDH*, *HPRT1*, *UBC* and *YWHAZ* genes were tested (with Hs00266705_g1, Hs99999909_m1, Hs00824723_m1 and Hs01122447_g1 Taqman probes, respectively); for Taqman assays *B2M*, *RPLP0*, *UBC* and *YWHAZ* genes were tested (with Hs00984230_m1, Hs0299885_s1, Hs 01871556_s1 and Hs03044281_g1 Taqman probes, respectively). PCR reactions were run in triplicate, using 25ng/μl of cDNA and 900 nM of primers (in SYBR Green-based analyses) and 9 ng/μl of cDNA and 250nM of Taqman probes (in Taqman-based analyses), in 20 μl of final volume. Reactions were carried out on an ABI Prism 7900HT Fast Real-Time PCR System Unit (Life Technologies) with standard cycling conditions. Serial cDNA dilutions were performed to calculate standard curves in order to determine the PCR efficiency for each gene. Results were analyzed with the Sequence Detection System (SDS) Software v2.4 (Life Technologies) to obtain the Cq (quantification cycle) values for each sample. Samples with >0.5 standard deviation were excluded.

The geNorm algorithm included in DataAssist v2.0 software (http://www.lifetechnologies.com/us/en/home/technical-resources/software-downloads/dataassist-software.html) was used to estimate the stability of genes for normalization. cDNA quantity was normalized relative to *UBC* and *YWHAZ* reference genes in SYBR Green assays and to *B2M* and *YWHAZ* reference genes in Taqman assays. The comparisons in gene expression levels between groups were carried out with Relative Expression Software (REST) [61] for SYBR Green data analyses and with DataAssist software for Taqman data analyses.

DNA samples from the Basque Country were genotyped for rs2742976 *E2F2* with the same methodology as for the samples collected from REGISTRY. *E2F2* mRNA levels were compared among the different rs2742976 *E2F2* genotypes.

## Results

### Absence of geographical stratification in the analyzed patient sample

The eAO of the EHDN sample analyzed in this work ranged from 21 to 73 years. Similarly, the mAO ranged from 21 to 70 years. Mean and median CAGexp values were very similar in both eAO and mAO (mean values of 44.11±2.91 and 44.30±3.11, respectively, and a median value of 44 CAGs in both AOs).

Given the diverse origin of the HD patients tested in this study, several statistical analyses were applied to examine possible genetic divergences between Southern European and Northern European individuals, which could lead to false positive associations [30]. No significant differences were detected when the mean and the median of CAGexp, eAO or mAO were compared (Tables 1 and 2). Similarly, the eAO and mAO distributions across CAGexp alleles did not show different AO patterns between Southern and Northern European populations (P = 0.958 (eAO) and P = 0.945 (mAO) in Kolmogorov-Smirnov test) (S1 and S2 Figs, respectively). Finally, stratification analysis using the SNP genotype information for all analyzed loci reflected low and no significant level of genetic differentiation between the two groups (F_ST_ index = 0.001±0.001; P = 0.5). Therefore, subsequent analyses were carried out without correcting for the geographical origin of the patients.

**Table 1:** CAGexp and eAO comparisons between samples from Southern and Northern European populations.

Origin of samples	N	Mean CAGexp	Median CAGexp	Mean eAO	Median eAO
Southern European	63	44.36±2.82	43	43.16±11.19	42
Northern European	190	44.03±2.95	44	43.07±11.24	42.5
Total	253	44.11±2.91	44	43.09±11.21	42
P value in Southern/Northern comparison	-	0.371	0.290	0.997	0.991

**Table 2:** CAGexp and mAO comparisons between samples from Southern and Northern European populations.

Origin of samples	N	Mean CAGexp	Median CAGexp	Mean mAO	Median mAO
Southern European	42	44.45±3.11	43.5	42.86±11.22	41
Northern European	136	44.25±3.12	44	44.09±11.65	43.5
Total	178	44.30±3.11	44	43.80±11.53	43
P value in Southern/Northern comparison	-	0.714	0.655	0.545	0.535

### Correlations between HD age of onset and genetic variation in candidate genes

Consistent with previous studies, CAGexp was significantly and negatively correlated with eAO (P<0.0001, R = -0.758) and mAO (P<0.0001, R = -0.824). In our sample, CAGexp accounted for 57.3% of eAO variability and 67.6% of mAO variability (Tables 3 and 4).

**Table 3:** Multiple linear regression analysis between SNP genotypes and eAO.

Model	Minor allele	Genetic model	Adjusted R^2^	Uncorrected P-value	Genotype	N	Mean CAGexp±SD	Mean eAO±SD
*HTT* CAGexp (1)	־	־	0.573	<0.0001	־	253	44.11±2.91	43.09±11.21
(1) *+ E2F2* rs2742976	T	ADD	0.583	0.001	TT	36	44.17±3.00	44.44±12.03
					GT	99	44.18±3.10	44.44±10.98
		DOM	0.589	<0.001	GG	113	43.97±2.75	41.70±11.10
					GT+TT	135	44.18±3.06	44.44±11.22
(1) *+ LINC01559* rs12423809	C	REC	0.575	0.048	CC	40	44.07±3.32	45.35±11.10
					AC+AA	208	44.07±2.85	42.78±11.26
(1) *+ LINC01559* rs10845757	T	DOM	0.577	0.025	CC	91	43.76±3.08	45.52±11.47
					CT+TT	155	44.26±2.82	41.75±10.86
(1) *+ GRIN2B* rs10744030	A	REC	0.580	0.033	AA	30	45.17±3.17	42.40±10.44
					AG+GG	220	43.96±2.87	43.22±11.40
(1) *+ GRIN2B-ATF7IP* rs7966469	T	REC	0.580	0.022	TT	17	43.65±3.26	40.59±11.75
					CT+CC	232	44.13±2.90	43.34±11.25
(1) *+ ATF7IP* rs11055896	C	ADD	0.576	0.034	GG	102	44.52±3.10	42.37±10.73
					CG	116	43.70±2.67	44.57±11.49
		REC	0.580	0.009	CC	29	44.03±3.10	40.07±11.10
					CG+GG	218	44.08±2.90	43.54±11.17
(1) *+ ATF7IP* rs3213764	G	REC	0.582	0.005	GG	61	43.80±3.16	46.49±11.96
					AG+AA	187	44.16±2.84	42.18±10.82
(1) *+ GRIN2A* rs8049651	T	REC	0.581	0.020	CT+CC	234	44.17±2.94	43.23±11.26
		ADD	0.587	0.003	TT	16	43.25±2.67	41.50±11.72
					CT	99	44.54±2.91	41.05±11.06
		DOM	0.583	0.010	CC	135	43.89±2.95	44.84±11.17
					CT+TT	115	44.36±2.90	41.11±11.10

Only models with uncorrected P-value <0.05 are shown. The minor allele based dominant (DOM), recessive (REC) and additive (ADD) genetic models were tested for their association with eAO by linear regression analysis. The dominant model of *E2F2* rs2742976 achieved a Bonferroni corrected P-value = 0.016.

**Table 4:** Multiple linear regression analysis between SNP genotypes and mAO.

Model	Minor allele	Genetic model	Adjusted R^2^	Uncorrected P-value	Genotype	N	Mean CAGexp±SD	Mean eAO±SD
*HTT* CAGexp (1)	־	־	0.676	<0.0001	־	178	44.30±3.11	43.80±11.53
(1) *+ E2F2* rs2742976	T	ADD	0.680	0.014	TT	21	44.14±3.20	45.95±12.50
					GT	71	44.45±3.33	44.62±11.70
		DOM	0.682	0.008	GG	83	44.14±2.91	42.90±11.09
					GT+TT	92	43.48±2.92	44.92±11.83
(1) *+ GRIK2* rs2782901	C	REC	0.685	0.018	CC	25	44.28±2.90	41.52±12.92
					CT+TT	151	44.30±3.17	44.22±11.36
(1) *+ HIP1* rs2240133	T	ADD	0.680	0.022	TT	20	44.35±2.60	41.60±12.30
					CT	73	44.12±2.76	43.85±11.44
		DOM	0.678	0.043	CC	76	44.68±3.57	43.82±11.49
					CT+TT	93	44.17±2.71	43.36±11.60
(1) *+ LINC01559* rs10845757	T	ADD	0.679	0.048	TT	29	44.31±2.69	42.44±9.44
					CT	75	44.55±3.21	42.17±12.10
		DOM	0.682	0.019	CC	70	43.97±3.21	46.20±11.37
					CT+TT	104	44.48±3.06	42.25±11.38
(1) *+ GRIN2B* rs10744030	A	REC	0.682	0.044	AA	23	44.87±3.48	44.56±11.45
					AG+GG	153	44.22±3.07	43.72±11.65
(1) *+ GRIN2B* rs4764011	G	REC	0.681	0.036	GG	34	44.03±3.70	42.85±12.63
					AG+AA	134	44.44±3.02	43.78±11.29
(1) + *GRIN2A* rs8049651	T	ADD	0.686	0.015	TT	9	43.89±2.15	42.00±11.54
					CT	73	44.71±3.10	41.58±11.42
		DOM	0.685	0.020	CC	94	44.02±3.22	45.77±11.50
					CT+TT	82	44.62±3.01	41.62±11.37

Only models with uncorrected P-value <0.05 are shown. The minor allele based dominant (DOM), recessive (REC) and additive (ADD) genetic models were tested for their association with mAO by linear regression analysis. The SNPs do not achieved P <0.05 values with Bonferroni correction.

The association analysis carried out using genotype data of 88 single nucleotide polymorphisms, CAGexp and HD age of onset, revealed eight association signals (uncorrected P-value <0.05) with eAO (Table 3) and seven with mAO (Table 4). Four of the association signals overlapped in eAO and mAO: rs2742976 in the *E2F2* gene, rs10845757 in the *LINC01559* gene, rs10744030 in the *GRIN2B* gene, and rs8049651 in the *GRIN2A* gene. In contrast, SNPs located in the *ATF71P* gene (rs11055896 and rs3213764), in the region between *ATF7IP* and *GRIN2B* gene promoter regions (rs7966469) and in *LINC01559* (rs12423809), only exhibited suggestive association with eAO, whereas SNPs mapping the *GRIK2* (rs2782901), the *HIP1* (rs2240133) and the *GRIN2B* (rs4764011) genes showed significant association only with mAO. With the exception of rs3213764 within *ATF7IP*, which is an exonic missense SNP, and rs8049651 within *GRIN2A*, which is a synonymous SNP, all other AO-associated SNPs in our study lie within non-coding regions of the genome.

Of note, six SNPs that correlated with eAO and three that correlated with mAO reside within *ATF7IP*, *GRIN2B* and *LINC01559* genes, all of which are located in the same genomic region (12p13.1), but not in *LD* (with the exception of rs12423809 and rs10845757, both in *LINC01559* gene, with a D’ value of 0.93 in our sample).

Remarkably, the observed *E2F2* association signal was significant after multiple test correction (Bonferroni corrected P-value < 0.05) and explained as much as 5.1% of the eAO variability not explained by CAGexp. More specifically, the T allele of the rs2742976 SNP of the *E2F2* gene significantly associated with a 3 year AO delay.

### *E2F2* gene expression analysis relative to the rs2742976 genotype in HD patients

Interestingly, rs2742976 is located within a putative STATx transcription factor-binding site [62] in the promoter region (-289 G>T) of the *E2F2* gene, suggesting that this polymorphism may affect *E2F2* gene expression. Therefore, we tested if there was a correlation between the rs2742976 genotype and *E2F2* gene expression in an independent HD patient sample (N = 35) collected in the Basque Country. From each individual, DNA and RNA samples were extracted from peripheral blood mononuclear cells. DNA samples were used to establish the *E2F2* rs2742976 genotype, and total RNA to quantify the relative *E2F2* mRNA expression. To ensure the robustness of the results, two methods were used (Taqman-based and SYBR Green-based assays) to measure the steady-state *E2F2* mRNA expression in HD individuals.

**Fig 1:**
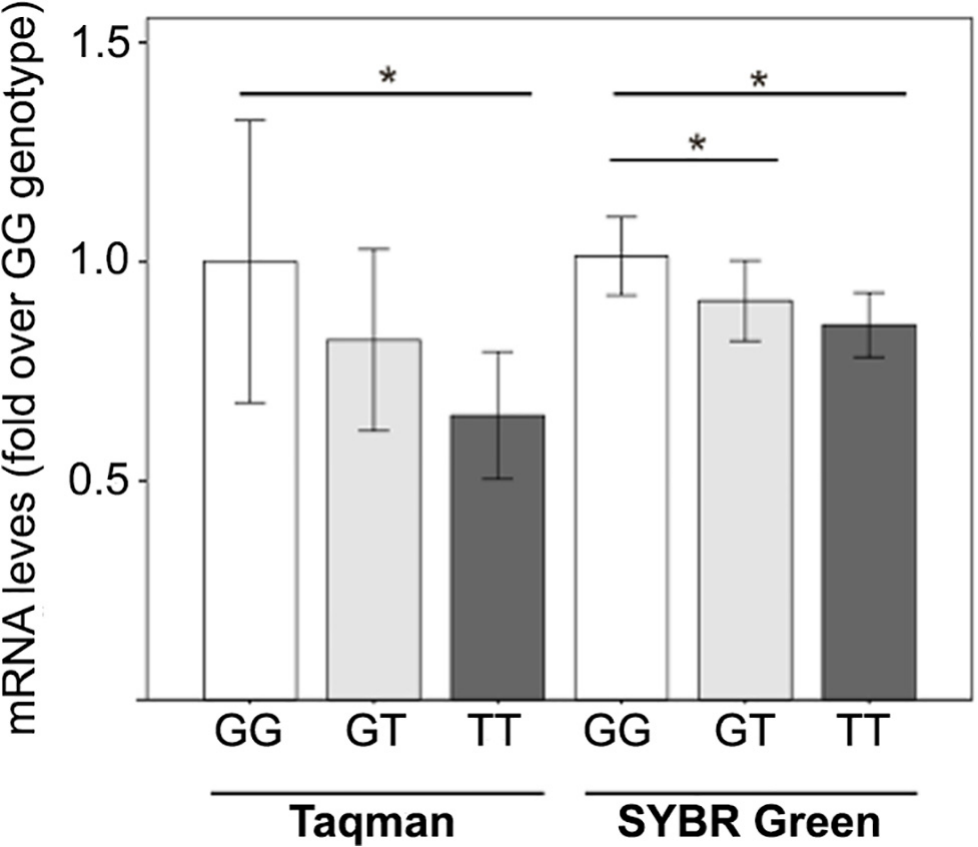
RT-qPCR analysis of *E2F2* gene expression in HD patients, according to *E2F2* rs2742976 genotype. Two methods were used. In Taqman assay, the expression of *E2F2* gene was analyzed in 31 samples (N _TT_ = 4; N _GT_ = 12, N _GG_ = 15) with Hs00918089_m1 Taqman probe; the expression values were normalized respect to expression of *B2M* and *YWHAZ* reference genes. In SYBR Green assay, the *E2F2* gene expression was estimated in 31 samples (N_TT_ = 5; N_GT_ = 14, N_GG_ = 12); the expression values were normalized to expression of *UBC* and *YWHAZ* reference genes. Results are expressed as fold over respective GG individuals. Asterisk denotes statistically significant differences (P<0.05) between GG and any other group, according to DataAssist software analysis (T-test) or REST software analysis (Pair Wise Reallocation Randomization test).

Interestingly, a significant correlation between *E2F2* rs2742976 genotype and *E2F2* gene expression was observed (Fig 1). Specifically, individuals with TT genotype showed significantly lower *E2F2* mRNA expression relative to individuals with GG genotype (P = 0.020 and P = 0.046 in Taqman-based assay and SYBR Green-based assays, respectively). In addition, a significantly lower *E2F2* expression was detected in samples with GT genotype relative to samples with GG genotype in SYBR Green-based assays (P = 0.044). Altogether, these results suggest that the presence of the T allele in the *E2F2* rs2742976 promoter SNP may account for a lower *E2F2* gene expression.

## Discussion

Age of onset in HD is inversely correlated with the CAG repeat length in the mutated *HTT* allele. However, other genetic factors are thought to play a role in this complex character. In an attempt to extract as much information as possible from our sample, we have carried out association analyses considering two AO definitions, mAO and eAO. Testing eAO has the advantage of encompassing all the HD phenotypes (motor, cognitive and psychiatric), which involves all the patients available in the sample; analyzing mAO, although it results in a smaller sample size, is considered more reproducible due to its specific nature and reliability of this criterion for determining AO [4,63].

In our study, the detected mAO variability explained by CAGexp (67.6%) lies within the range of previously observed values for motor onset age in Europeans (31–73%) [9,16], and is comparable to that reported by Ramos and collaborators (65.3%), who considered the same CAG repeat range as in our study [30]. Regarding eAO, to our knowledge no other reports have examined eAO variability within the CAG range considered in our work, and no direct comparisons can be made. However, the eAO variability explained by CAGexp obtained by us (57.3%) is well within the range of 49% to 73% detected in other general studies [6,64].

Among the detected AO-associated signals, the association between the *E2F2* gene and the age of onset is particularly compelling (Bonferroni corrected P-value <0.05). In our study, individuals with the *E2F2* rs2742976 T allele showed on average a 3-year delay in eAO. The relationship between *E2F2* gene and HD has not been previously assessed. *E2F2* encodes a transcription factor that regulates the cell cycle, and is known to play a critical role in lymphocyte quiescence [65] and in neuronal terminal differentiation [36], through the regulation of target gene expression. Interestingly, SNP rs2742976 is located within a putative STATx transcription factor-binding site in the *E2F2* promoter region [62], and allele differences in this SNP could potentially modulate the expression of the *E2F2* gene itself. Indeed, HD patients with *E2F2* rs2742976 T allele showed significantly lower *E2F2* mRNA expression levels in lymphocytes (P-value <0.05). This change in expression level seems to be dependent on T allele dose, given that the heterozygote (GT) shows intermediate expression levels relative to the two homozygous genotypes. Thus, the detected association between the T allele in *E2F2* rs2742976 and both a delay in eAO and lower *E2F2* gene expression level in lymphocytes hints to a potential involvement of *E2F2* in the pathogenesis of HD, a possibility that warrants further study. Moreover, although HD pathology is thought to involve mainly brain-associated defects, our observations support the view that studies on gene expression profiling in blood cells and other peripheral tissues could help identify biomarkers for HD disease progression [66–68] and provide clues to HD pathology.

None of the other suggestive eAO-association signals were significant after multiple test correction, although some of them may be worth examining further. Genetic variants within the *GRIN2A* and *GRIN2B* genes encoding the NR2A and NR2B subunits of the N-methyl-D-Aspartic acid (NMDA) receptors [51], have been associated previously with HD age of onset [16–18], although the molecular mechanism involved in these associations remains to be elucidated [18]. This is the case of the polymorphisms rs2650427 [18] and rs1969060 [16,18,69] within the *GRIN2A* gene, and the polymorphisms rs1806201 [16,18] and rs890 [16] within the *GRIN2B* gene. Two of those SNPs (*GRIN2A* rs1969060 and *GRIN2B* rs1806201) did not associate with AO in our study, but other genetic variants within the *GRIN2A* and the *GRIN2B* genes showed uncorrected P-values < 0.05: rs8049651 in *GRIN2A*, and rs10744030, rs4764011 and rs7966469 in *GRIN2B*. Of these, only rs4764011 had been previously analyzed, although no association to mAO had been detected [17]. Intriguingly, four additional suggestive signals that were not in *LD* were detected in the vicinity of the *GRIN2B* gene, encompassing the genes *ATF7IP* and *LINC01559*. These three genes extend over 2 Mb in 12p13.1, a genomic region previously related with intellectual disability [70]. Most associated SNPs mapping *GRIN2A* and the 12p13.1 region are located in non-coding regions, which raises the possibility that they may affect chromatin organization [71].

The detected mAO-association signals were not significant after Bonferroni correction (probably due to the smaller size of this sample). However, suggestive signals specific for the mAO analysis may be worth following in a bigger sample. In this regard, a non-coding polymorphism mapping the *HIP1* gene (rs2240133) was found associated specifically with mAO. *HIP1* encodes an HTT-interacting protein known to be involved in apoptosis [72,73], and mutant *HIP1* expression produces HTT aggregation and subsequent cell death [74]. The relationship between *HIP1* polymorphisms and HD AO has not been reported before. Similarly, genetic variation within the *GRIK2* gene (rs2782901) suggestively associated with mAO but no eAO. *GRIK2* encodes the GluR6 subunit of the kainate glutamate receptors, which are involved in synaptic plasticity [75]. Mutations in the *GRIK2* gene have been repeatedly associated with HD AO [10,14,15].

In summary, our work, which should be considered a hypothesis-generating study, has explored the contribution of genetic variation in several candidate genes as eAO and mAO modifiers. The observed statistically significant and suggestive association signals should be followed-up to improve our knowledge of HD pathogenesis. Importantly, the highly significant *E2F2* signal should be further studied given that the presence of the T allele at *E2F2* rs2742976 associates with HD age of onset and with *E2F2* gene expression levels. We speculate that lower levels of *E2F2* gene expression in HD symptomatic patients could be associated with a delay in AO.

## Supporting Information

S1 Appendix Contributor information. Members of the European Huntington’s Disease Network (EHDN) that have collaborated in collecting biological samples and clinical data.
(PDF)

S1 Fig Box plot representing the variance of eAO per CAGexp in samples from Southern and Northern European populations. No differences were observed in eAO distribution across CAGexp alleles between both groups.
(TIF)

S2 Fig Box plot representing the variance of mAO per CAGexp in samples from Southern and Northern European populations. No differences were observed in mAO distribution across CAGexp alleles between both groups.
(TIF)

S1 Table EHDN’s dataset general information. Age: patient’s age at the time of blood collection; Sex: M (male) and F (female); Ethnicity: 1 = Caucasian; 2 = American-Black; 3 = Asian-West. Country: country of origin. CAGexp: CAG number repeat in expanded allele; eAO: earliest onset, age of the earliest unequivocal symptoms of Huntington's onset; mAO: motor onset, age of the first motor symptoms §: samples excluded from the analysis; * samples with eAO information excluded from the analysis.-: No data.
(PDF)

S2 Table General information and expression analysis (RT-qPCR analysis) of the HD patients from Basque Hospitals. Age: patient’s age at the time of blood collection; Sex: M (male) and F (female); CAGexp: CAG number repeat in expanded allele; Genotypes in *E2F2* rs2742976 and Cq values in RT-qPCR analysis for the *E2F2* and reference genes analyzed using SYBR Green and Taqman based assays are showed. NA: Not Analyzed.
(PDF)

S3 Table SNPs genotyped in this study. For each SNP, the genomic region (cytoband) and the closest gene is defined as well as the SNP type, chromosome location (according to genome assembly GRCh38), minor and major allele, HapMap CEU minor allele frequency (MAF), genotyping quality control (QC) notes and references if they have been previously studied in HD. * Indicates tag-SNPs. HWE: Hardy-Weinberg Equilibrium.
(PDF)

S4 Table Genotypes in the SNPs analyzed in each DNA sample from EHDN’s collection. (PDF)
