# Supplementary material for: Exploring Genetic Factors Involved in Huntington Disease Age of Onset: E2F2 as a New Potential Modifier Gene
Source: PLoS One. 2015 Jul 6;10(7):e0131573. doi: 10.1371/journal.pone.0131573 (PMC4493078; doi:10.1371/journal.pone.0131573)
Supplement: S1 Appendix — Members of the European Huntington’s Disease Network (EHDN) that have collaborated in collecting biological samples and clinical data. (PDF) [file pone.0131573.s001.pdf]

S1 Appendix. Contributor information.
Members of the European Huntington's Disease Network (EHDN) that have collaborated in collecting biological samples and clinical data.

Language coordinators:

RM Bonelli, **LKH Graz, Abteilung für Psychiatrie, Graz, Austria.** C Verellen-Dumoulin, **Institut de Pathologie et de Génétique, Charleroi, Belgium.** W Vandenberghe, **University Ziekenhuis Gasthuisberg, Leuven, Belgium.** P Sasinková, **Rada SPHCH, Náchod, Czech Republic.** T Uhrov, **Charles University in Prague and General University Hospital in Prague, Prague, Czech Republic.** JE Nielsen, **Hukommelsesklinikken, Rigshospitalet, Copenhagen, Denmark.** AC Bachoud-Lévi; A Rialland, **Centre Hospitalier Universitaire Henri-Mondor, Créteil, France.** H Padieu, **Groupe Hospitalier Chenevier-Mondor, Créteil, France.** M Päivärinta; R Bos, **University of Turku and Turku University Hospital, Turku, Finland.** K Barth; D Ecker; C Held; GB Landwehrmeyer; M Orth, **Ulm University, Ulm, Germany.** T Illmann, **University of Rostock, Ulm, Germany.** I Biunno, **Institute of Genetics and Biomedical Research-National Research Council, Milan, Italy.** D Monza, **Fondazione IRCCS Istituto Neurologico Carlo Besta, Milan, Italy.** AR Bentivoglio, **Istituto di Neurobiologia e Medicina Molecolare CNR/ Istituto di Neurologia, Dipartimento di Neuroscienze/ CNR Istituto di Scienze e Tecnologie della Cognizione, Rome, Italy.** RAC Roos; M-N Witjes-Ané, **Leiden University Medical Centre (LUMC), Leiden, Netherlands.** A Heiberg; M van Walsem, **Rikshospitalet, Department of Medical Genetics, Oslo, Norway.** S Pro Koivisto; N Røren (formerly Heinonen), **Oslo University Hospital, Ulleval, Oslo, Norway.** D Zielonka, **Medical University of Poznań, Poznan, Poland.** J Zaremba, **Institute of Psychiatry and Neurology Department of Genetics, Warsaw, Poland**. JJ Ferreira; T Mestre, **Hospital de Santa Maria, Lisbon, Portugal.** A Rojo Sebastián, **Hospital Universitario Mútua de Terrassa, Barcelona, Spain.** Maria Dolores Martinez-Jaurrieta, **Complejo Hospitalario de Navarra, Pamplona, Spain**. A Martínez Descals, **Madrid-Fundación Jiménez Díaz, Madrid, Spain.** M Bascuñana Garde; P Trigo Cubillo, **Hospital Ramón y Cajal, Madrid, Spain.** J Wahlström, **Sahlgrenska University Hospital, Göteborg, Sweden.** Jean-Marc Burgunder, **Neurologische Klinik des Inselspitals, Bern, Switzerland.** SB Dunnett; OJ Handley; J Townhill (formerly Naji), **Schools of Medicine and Biosciences, Cardiff University, Cardiff, UK.** M Laurá; SJ Tabrizi, **UCL Institute of Neurology and National Hospital for Neurology and Neurosurgery, London, UK.** J Levey, **University of Massachusetts Medical School, Worcester, USA.**

Participating Investigators:

AUSTRIA
Graz (LKH Graz, Abteilung für Psychiatrie): Raphael M. Bonelli; Brigitte Herranhof; Anna Holl; Hans-Peter Kapfhammer; Michael Koppitz; Markus Magnet; Daniela Otti; Annamaria Painold; Karin Reisinger
Innsbruck (Universitätsklinik für Neurologie): Florian Brugger; Caroline Hepperger; Anna Hotter; Philipp Mahlknecht; Michael Nocker; Klaus Seppi; Gregor Wenning

BELGIUM
Charleroi (Institut de Pathologie et de Génétique (IPG)): Pascale Ribaï; Christine Verellen-Dumoulin

CZECH REPUBLIC
Prague (Centrum extrapyramidových onemocnění): JiříKlempíř; Martin Kucharík; Jan Roth

DENMARK
Copenhagen (Hukommelsesklinikken, Rigshospitalet): Lis Hasholt; Lena E. Hjermind; Oda Jakobsen; Jorgen Nielsen; Anne Nørremølle; Sven Asger Sørensen; Jette Stokholm

FINLAND
Tampere (Terveystalo Healthcare Service Centre); Maire Santala.
Turku-Suvituuli (Rehabilitation Centre Suvituuli): Heli Hiivola; Kirsti Martikainen; Katri Tuuha

GERMANY
Aachen (Universitätsklinikum Aachen, Neurologische Klinik): Christoph Michael Kosinski; Daniela Probst; Christian Sass; Johannes Schiefer; Christiane Schlangen; Cornelius J. Werner
Bochum (Huntington-Zentrum (NRW) Bochum im St. Josef-Hospital): Jürgen Andrich; Rainer Hoffmann; Christian Prehn; Carsten Saft; Stephan Salmen; Katrin Straßburger
Dinslaken (Reha Zentrum in Dinslaken im Gesundheitszentrums Lang): Herwig Lange
Dresden (Universitätsklinikum Carl Gustav Carus an der Technischen Universität Dresden, Klinik und Poliklinik für Neurologie): Matthias Löhle; Alexander Storch; Anett Wolz; Martin Wolz
Freiburg (Universitätsklinik Freiburg, Neurologie): Johann Lambeck; Birgit Zucker
Hamburg (Universitätsklinikum Hamburg-Eppendorf, Klinik und Poliklinik für Neurologie): Alexander Münchau; Lars Stubbe; Simone Zittel
Hannover (Neurologische Klinik mit Klinischer Neurophysiologie, Medizinische Hochschule Hannover): Gabriele Diercks; Dirk Dressler; Heike Gorzolla; Christoph Schrader; Pawel Tacik
Heiligenhafen (Psychatrium Heiligenhafen): Walburgis Heinicke
Münster (Universitätsklinikum Münster, Klinik und Poliklinik für Neurologie): Stefan Bohlen; Herwig Lange; Ralf Reilmann
Taufkirchen (Isar-Amper-Klinikum - Klinik Taufkirchen (Vils)): Antonie Beister; Matthias Dose; Kathrin Hammer; Gabriele Leythaeuser; Ralf Marquard; Tina Raab; Caroline Schrenk; Michele Schuierer; Alexandra Wiedemann
Ulm (Universitätsklinikum Ulm, Neurologie): Daniel Ecker; Carolin Eschenbach; Bernhard Landwehrmeyer; Franziska Lezius; Solverigh Nepper; Michael Orth; Sigurd Süßmuth; Sonja Trautmann

ITALY
Bari (Dipartimento di Scienze Neurologiche e Psichiatriche UNIVERSITA' DI BARI): Claudia Cormio; Olimpia Difruscolo; Marina de Tommaso; Vittorio Sciruicchio; Claudia Serpino

Genova (Dipartimento di Neuroscienze, Oftalmologia e Genetica (DiNOG) Università di Genova): Giovanni Abbruzzese; Emilio Di Maria; Monica Bandettini di Poggio; Giovanna Ferrandes; Paola Mandich; Roberta Marchese
Milan (Fondazione IRCCS Istituto Neurologico Carlo Besta, Milan): Alberto Albanese; Stefano Di Donato; Caterina Mariotti; Paola Soliveri; Cinzia Gellera; Daniela Monza; Chiara Tomasello; Lorenzo Nanetti.
Naples (Azienda Ospedaliera Universitaria Federico II - Dipartimento di Scienze Neurologiche): Di Maio Luigi; Giuseppe De Michele; Carlo Rinaldi; Cinzia Russo; Elena Salvatore; Tecla Tucci
Pozzilli (Neurogenetics Unit – IRCCS Neuromed): Ferdinando Squitieri; Tiziana Martino; Sara Orobello; Silvia Alberti; Francesca De Gregorio; Valentina Codella; Nunzia De Nicola; Vittorio Maglione.
Rome (Istituto di Neurobiologia e Medicina Molecolare CNR/ Istituto di Neurologia, Dipartimento di Neuroscienze/ CNR Istituto di Scienze e Tecnologie della Cognizione): Anna Rita Bentivoglio; Alfonso Fasano; Marina Frontali; Arianna Guidubaldi; Tamara Ialongo; Gioia Jacopini; Giovanna Loria; Carla Piano; Silvia Romano; Francesco Soleti; Maria Spadaro; Paola Zinzi

NETHERLANDS
Enschede (Medisch Spectrum Twente): Monique S.E. van Hout; Jeroen P.P. van Vugt; A. Marit de Weert
Leiden (Leiden University Medical Centre (LUMC)): Reineke Bos; Eve M. Dumas; Caroline K. Jurgens; Simon J. A. van den Bogaard; Raymund A.C. Roos; Marie-Noëlle Witjes-Ané
Nijmegen (Universitair Medisch Centrum St. Radboud, Neurology): Berry Kremer; C.C.P. Verstappen

NORWAY
Oslo-RH (Rikshospitalet, Dept. of Medical Genetics): Arvid Heiberg; Marleen R van Walsem
Trondheim (St. Olavs Hospital): Inga Bjørnevoll; Sigrid Botne Sando

POLAND
Gdansk (Specialistic Hospital, Gdansk Zaspa): Jaroslaw Slawek; Witold Soltan; Emilia Sitek.
Katowice (Silesian Medical University Katowice): Magdalena Boczarska-Jedynak; Barbara Jasinska-Myga; Gregorz Opala.
Krakow (Krakowska Akademia Neurologii): Andrzej Szczudlik; Monika Rudzińska; Magdalena Wójcik, Krzysztof Banaszkiewicz; Malgorzata Krawczyk.
Poznan (Medical University of Poznań): Daniel Zielonka; Jerzy Marcinkowski; Anna Ciesielska; Justyna Sempołowicz; Anna Bryl; Aneta Klimberg.
Warsaw-MU (Medical University of Warsaw, Neurology): Piotr Janik; Anna Kalbarczyk; Hubert Kwiecinski; Zygmunt Jamrozik.
Warsaw-IPiN (Institute of Psychiatry and Neurology Dep. of Genetics, Dep. of Neurology): Grzegorz Witkowski; Danuta Ryglewicz; Jakub Antczak; Maria Rakowicz; Katarzyna Jachinska; Elzbieta Zdzienicka; Przemyslaw Richter; Jacek Zaremba.

PORTUGAL

Lisbon-Santa Maria (Neurological Clinical Research Unit, Institute of Molecular Medicine): Miguel Coelho; Joaquim J Ferreira; Tiago Mestre; Mário M Rosa; Anabela Valadas.
Porto-São João (Hospital São João E.P.E.): Miguel Gago; Carolina Garrett; Maria Rosalia Guerra

SPAIN
Burgos (Servicio de Neurología Hospital General Yagüe): Esther Cubo; Natividad Mariscal; Jesús Sánchez
Madrid RYC (Hospital Ramón y Cajal, Neurología): Mónica Bascuñana; Patricia Trigo Cubillo; Marta Fatàas; José Luis López Moreno; Guillermo García Ribas; Christine Schwarz, Justo García de Yébenes
Madrid FJD: (Madrid-Fundación Jiménez Díaz): María José Saiz Artiga; Asunción Martínez-Descals; Pedro J García Ruíz; Vicenta Sánchez
Barcelona (Hospital Universitario Mútua de Terrassa): Ana Rojo Sebastian; Miquel Aguilar Barber; Dolors Badenes Guia; Laura Casas Hernan; Gemma Tome Carruesco; Esther Suarez San Martin; Judit López Catena
Barcelona-Bellvitge (Hospital Universitari de Bellvitge): Jordi Bas; Matilde Calopa; Núria Busquets
Barcelona-Merced (Hospital Mare de Deu de La Merced): Marina Dalmau Elorza; Cristóbal Díez-Aja López; Santiago Durán-Sindreu Terol; Misericordia Floriach Robert; Belén Garzón Ruíz; Ana González Casado; Isabel Haro Martínez; Celia Mareca Viladrich; Regina Pons i Càrdenas; Elvira Roca; Joan Roig Llesoy; Jesús Miguel Ruiz Idiago; Mar Ruíz Vergara; Socorro Soriano García; Antonio Villa Riballo

SWEDEN
Umeå (Norrlands Universitet Sjukhus, Department of Neurology): Ghada Loutfi; Eva-Lena

U.K.
Birmingham (The Barberry Centre, Dept of Psychiatry): Jenny de Souza; Hugh Rickards; Jan Wright
Cambridge (Cambridge Centre for Brain Repair, Forvie Site): Roger A. Barker; Kate Fisher; Anna Olivia Goyder Goodman; Susan Hill; Ann Kershaw; Sarah Mason; Nicole Paterson; Lucy Raymond
Cardiff (Schools of Medicine and Biosciences, Cardiff University): Jonathan Bisson; Monica Busse; Catherine Clenaghan; Ruth Fullam; Stephen Dunnett; Olivia Handley; Alis Hughes; Sarah Hunt; Lesley Jones; Una Jones; Hanan Khalil; Sara Minster; Michael Owen; Kathleen Price; Jenny Townhill; Anne Rosser
Fife (Scottish Huntington's Association Whyteman's Brae Hospital): Peter Brockie; Jillian Foster; Nicola Johns; Jean Rother; Gareth Thomas; Shona Yates
Leeds (Chapel Allerton Hospital, Department of Clinical Genetics): Carol Chu; Emma Hobson; Stuart Jamieson; Jean Toscano; Sue Wild; Pam Yardumian
London (The National Hospital for Neurology and Neurosurgery): Thomasin Andrews; Stefania Bruno; Elvina Chu; Karen Doherty; Nayana Lahiri; Marianne Novak; Aakta Patel; Sarah Tabrizi; Rachel Taylor; Thomas Warner; Edward Wild
Manchester (Genetic Medicine, University of Manchester, Manchester Academic Health Sciences Centre and Central Manchester University Hospitals NHS Foundation Trust): Natalie Arran; David Craufurd; Ruth Fullam; Liz Howard; Susan

Huson; Lucy Partington-Jones; Nichola Verstraelen (formerly Ritchie); Julie Snowden; Andrea Sollom; Cheryl Stopford; Jennifer Thompson; Leann Westmoreland
Oxford (Oxford Radcliffe Hospitals NHS Trust): Andrea H Nemeth; Gill Siuda
Sheffield (The Royal Hallamshire Hospital): Oliver Bandmann; Alyson Bradbury; Kay Fillingham; Isabella Foustanos; Katherine Tidswell; Oliver Quarrell
