# Supplementary material for: Exploring Genetic Factors Involved in Huntington Disease Age of Onset: E2F2 as a New Potential Modifier Gene
Source: PLoS One. 2015 Jul 6;10(7):e0131573. doi: 10.1371/journal.pone.0131573 (PMC4493078; doi:10.1371/journal.pone.0131573)
Supplement: S1 Table — Age: patient’s age at the time of blood collection; Sex: M (male) and F (female); Ethnicity: 1 = Caucasian; 2 = American-Black; 3 = Asian-West. Country: country of origin. CAGexp: CAG number repeat in expanded allele; eAO: earliest onset, age of the earliest unequivocal symptoms of Huntington's onset; mAO: motor onset, age of the first motor symptoms §: samples excluded from the analysis; * samples with eAO information excluded from the analysis.-: No data. (PDF) [file pone.0131573.s004.pdf]

S1 Table. EHDN's dataset general information.

Age: patient's age at the time of blood collection; Sex: M (male) and F (female); Ethnicity: 1= Caucasian; 2=American-Black; 3=Asian-West. Country: country of origin. CAGexp: CAG number repeat in expanded allele; eAO: earliest onset, age of the earliest unequivocal symptoms of Huntington's onset; mAO: motor onset, age of the first motor symptoms §: samples excluded from the analysis; * samples with eAO information excluded from the analysis. -: No data.

Sample	Age	Sex	Ethnicity	Country	CAGexp	eAO	mAO
1	46	M	1	Germany	45	30	–
2	56	M	1	Austria	44	47	–
3	54	M	1	Germany	43	50	50
4	42	M	1	Germany	47	36	36
5	30	M	1	Germany	52	28	28
6	63	M	1	UK	41	57	57
7§	45	F	1	–	49	20	–
8	55	F	1	Italy	43	48	48
9	32	M	1	Italy	47	24	28
10	70	F	1	Netherlands	40	63	–
11	67	M	1	Finland	41	58	58
12	51	M	1	Germany	42	47	–
13§	60	F	1	Spain	43	51	54
14	45	M	1	Spain	48	30	–
15	50	F	1	Germany	43	29	49
16	52	F	1	Spain	44	42	32
17	44	M	1	Germany	44	41	–
18	59	F	1	Italy	42	59	59
19	69	M	1	UK	41	61	61
20	59	M	1	Netherlands	42	56	56
21§	35	F	1	Germany	51	28	30
22§	26	M	1	Spain	50	19	22
23	40	M	1	Portugal	45	34	34
24	62	F	1	UK	41	60	60
25	48	F	1	Austria	43	47	47
26	43	M	1	Spain	45	33	–
27	47	M	1	Netherlands	44	35	–
28	68	F	1	Spain	42	48	–
29	44	F	1	Norway	46	32	32
30	46	M	1	UK	45	38	38
31	43	M	1	UK	47	34	36
32	45	F	1	Netherlands	46	27	27
33	38	F	1	Norway	47	26	–
34	77	M	1	Spain	41	73	–
35	35	F	1	Germany	49	28	28
36	55	F	1	Germany	45	41	41
37	69	F	1	Austria	41	67	67
38	32	M	1	Norway	47	24	24
39	45	M	1	Spain	43	39	43

40	51	F	1	Italy	48	41	−
41	42	M	1	Netherlands	44	41	−
42	47	F	1	Netherlands	44	36	42
43	68	M	1	Germany	40	57	57
44	54	F	1	Netherlands	41	47	53
45	38	M	1	UK	46	30	30
46	53	F	1	Spain	46	30	−
47	40	M	1	Italy	46	32	32
48	57	F	1	Spain	47	35	35
49	60	M	1	Spain	43	50	50
50	54	M	1	Norway	43	43	43
51	48	M	1	Spain	45	40	−
52§	26	M	1	UK	55	22	−
53	33	M	1	Germany	51	29	29
54§	32	F	1	Spain	49	16	−
55	46	M	1	UK	47	44	44
56	35	F	1	Netherlands	42	32	−
57	45	F	1	UK	47	35	35
58	63	M	1	UK	43	59	59
59	56	F	1	Germany	43	49	48
60	57	M	1	Netherlands	42	51	51
61	28	F	1	Spain	50	28	28
62	72	F	1	Portugal	43	56	55
63§	44	F	1	Netherlands	39	39	−
64§	61	F	1	Norway	39	55	58
65	39	M	1	Norway	44	24	24
66	44	F	1	Italy	45	41	41
67	51	F	1	Norway	44	43	−
68	52	F	1	Germany	44	39	39
69	55	F	1	UK	42	46	46
70	38	F	1	Italy	44	30	30
71	61	M	1	Germany	40	57	57
72	46	M	1	Germany	46	35	−
73§	36	M	1	Germany	48	30	32
74	46	F	1	Germany	44	45	−
75	70	M	1	Netherlands	40	61	−
76	57	M	1	Germany	43	49	49
77*	67	M	1	UK	41	64	61
78	59	F	1	Netherlands	42	51	51
79	62	F	1	UK	42	49	54
80	42	M	1	Netherlands	44	39	−
81	38	M	1	Spain	43	27	41
82	54	M	1	Finland	41	50	−
83	42	F	1	UK	45	33	36
84	40	M	1	Italy	45	36	−
85	77	F	1	Spain	40	65	65
86	42	M	1	Austria	45	37	37
87	73	M	1	Germany	40	63	63
88	48	F	1	Poland	42	31	31

89	57	F	1	Germany	44	48	−
90[§]	71	F	2	UK	41	64	−
91	54	M	1	Netherlands	42	49	−
92	56	M	1	Poland	41	38	−
93	62	F	1	Denmark	40	59	59
94	33	F	1	Poland	40	30	−
95[§]	24	F	1	Poland	63	23	−
96	57	F	1	Poland	42	39	−
97[§]	18	M	1	Poland	67	17	17
98[§]	30	M	1	Poland	54	19	28
99	51	M	1	Italy	43	45	−
100[§]	33	M	1	Poland	51	23	23
101	42	M	1	Italy	48	26	26
102	52	M	1	Italy	43	46	46
103	40	F	1	Italy	47	35	35
104	57	F	1	Germany	43	50	−
105	43	F	1	Denmark	49	34	35
106	58	F	1	Portugal	42	46	−
107[§]	19	M	1	Poland	60	19	23
108	38	M	1	Germany	50	35	36
109	56	M	1	Spain	45	47	47
110	76	F	1	Netherlands	41	55	55
111	63	F	1	Italy	46	52	−
112	60	F	1	UK	43	45	47
113	61	M	1	Germany	42	44	44
114	63	F	1	Italy	43	55	−
115	50	M	1	UK	45	44	−
116	55	F	1	Netherlands	41	52	52
117	47	F	1	Poland	45	30	−
118	27	M	1	Poland	51	25	26
119	56	M	1	UK	41	51	−
120[§]	25	F	1	Poland	58	13	−
121[§]	63	M	1	Denmark	37	54	−
122	33	M	1	UK	49	25	25
123	61	M	1	Denmark	42	46	−
124	47	F	1	Poland	44	37	−
125	37	M	1	Germany	46	34	34
126[§]	46	F	3	Germany	47	43	43
127	77	F	1	UK	41	68	68
128	32	F	1	Netherlands	51	27	28
129[§]	73	M	1	Poland	39	36	71
130	52	F	1	Italy	47	43	−
131[§]	50	F	1	Germany	42	43	49
132	47	F	1	Spain	47	39	39
133	46	F	1	Denmark	46	40	−
134	73	M	1	UK	41	58	−
135	67	F	1	UK	43	56	56
136	39	F	1	Poland	48	31	31
137	65	F	1	Portugal	41	50	57

138	57	F	1	Spain	42	49	49
139	57	M	1	Italy	43	51	−
140	40	F	1	Germany	47	39	39
141	49	M	1	Denmark	43	45	45
142	66	M	1	Italy	42	54	−
143	41	F	1	UK	47	32	32
144	54	M	1	Netherlands	42	51	51
145	48	F	1	Poland	45	44	−
146	53	F	1	Portugal	43	49	50
147	56	M	1	Denmark	44	41	41
148	63	M	1	UK	41	52	52
149	34	M	1	Germany	40	29	−
150	60	F	1	Germany	40	49	49
151	48	M	1	Germany	43	45	45
152	64	M	1	UK	42	44	44
153	41	M	1	Poland	49	29	−
154	67	F	1	Denmark	42	64	63
155	59	F	1	Italy	42	44	−
156	42	M	1	UK	46	35	35
157	35	M	1	Germany	47	32	−
158	34	F	1	Poland	50	21	21
159	39	F	1	Norway	46	35	34
160§	52	F	1	Poland	40	48	48
161	56	M	1	Germany	42	50	−
162	48	F	1	Germany	43	46	46
163	36	M	1	UK	52	25	27
164	40	M	1	Netherlands	46	37	37
165	66	F	1	Italy	42	50	−
166	40	F	1	Netherlands	46	37	37
167§	25	M	1	Spain	65	22	−
168	34	F	1	Germany	46	33	−
169	29	M	1	Poland	41	27	−
170*	63	M	1	UK	40	58	52
171	64	M	1	Belgium	41	49	−
172	36	F	1	UK	46	34	34
173	27	M	1	Italy	52	21	22
174	40	F	1	Germany	45	31	31
175§	33	F	1	Italy	58	31	33
176	47	M	1	Germany	46	34	38
177	41	M	1	Germany	47	28	28
178	58	M	1	Italy	42	56	56
179	43	F	1	Germany	49	38	−
180	50	F	1	Netherlands	45	43	49
181	50	M	1	Germany	45	43	−
182	44	M	1	Germany	47	38	38
183	73	M	1	Italy	42	55	−
184	46	M	1	UK	46	42	41
185	54	M	1	Austria	44	41	41
186	59	F	1	Finland	44	50	−
187	48	M	1	Germany	43	45	47

188	52	F	1	Germany	45	33	47
189	61	M	1	Denmark	42	57	57
190	43	F	1	Belgium	44	40	−
191	58	F	1	Germany	43	52	53
192	54	M	1	Germany	41	48	48
193	63	F	1	UK	43	57	57
194	29	M	1	Italy	52	27	27
195	52	F	1	Poland	42	45	−
196	31	F	1	Poland	51	28	28
197	52	M	1	Germany	42	45	46
198	59	F	1	Finland	41	50	−
199	82	M	1	Norway	40	70	70
200	59	F	1	Sweden	40	48	55
201	45	M	1	Poland	44	37	−
202	45	M	1	Germany	44	41	41
203	72	M	1	Belgium	43	54	54
204	67	F	1	Poland	42	60	60
205	45	M	1	Germany	45	42	43
206	36	M	1	Germany	45	34	35
207	49	F	1	Italy	45	38	41
208	52	F	1	Germany	40	42	42
209	76	M	1	Italy	42	55	55
210[§]	24	F	1	Germany	68	14	15
211	65	M	1	UK	45	58	60
212	69	M	1	UK	40	64	66
213	36	M	1	Denmark	50	32	32
214[§]	54	M	1	UK	38	50	47
215	25	M	1	Germany	52	23	24
216	62	M	1	Austria	42	52	−
217	47	F	1	Netherlands	45	40	40
218	48	F	1	Poland	45	22	22
219	66	F	1	Germany	42	60	60
220	43	F	1	Germany	44	40	43
221	47	F	1	Italy	40	46	46
222[§]	21	F	1	Germany	70	12	12
223	61	M	1	Italy	41	60	60
224	47	M	1	Italy	45	35	−
225	61	F	1	Denmark	41	60	60
226[§]	68	F	1	Netherlands	38	50	−
227	70	F	1	UK	40	60	64
228	74	M	1	Italy	40	68	68
229	49	F	1	Italy	44	41	41
230	54	F	1	Poland	44	46	−
231	62	F	1	Italy	43	53	53
232	55	F	1	Germany	44	47	47
233	54	F	1	Czech republic	43	49	52
234[§]	47	F	1	Denmark	44	20	43
235	39	F	1	Germany	44	32	32
236	44	F	1	Germany	46	37	38

237	31	M	1	Poland	48	31	31
238	68	M	1	UK	43	54	54
239	47	M	1	Germany	44	37	37
240	31	F	1	Poland	50	21	35
241	46	M	1	Netherlands	45	42	42
242	58	F	1	Germany	41	56	56
243	70	M	1	Poland	42	66	−
244	42	F	1	Portugal	43	37	39
245	72	M	1	Poland	41	62	62
246	34	M	1	Italy	51	32	32
247	38	F	1	Germany	51	30	30
248	44	M	1	Germany	44	40	−
249	62	M	1	Belgium	42	58	58
250	58	F	1	Italy	44	47	47
251	42	F	1	Germany	43	41	−
252	34	M	1	Poland	46	32	32
253	43	M	1	Italy	47	32	−
254	43	M	1	Germany	46	32	32
255	31	M	1	Poland	51	30	30
256	61	F	1	Italy	42	52	53
257	57	M	1	Italy	43	56	−
258	63	M	1	Italy	43	41	−
259	64	M	1	UK	41	61	61
260	63	M	1	Austria	44	52	52
261	34	M	1	Italy	48	30	31
262	49	F	1	Germany	44	47	47
263	62	F	1	Czech republic	42	53	53
264	51	F	1	UK	44	48	48
265	59	F	1	UK	41	52	−
266	39	F	1	Netherlands	43	34	42
267	44	M	1	Spain	44	38	38
268	44	F	1	Netherlands	48	37	37
269	75	M	1	Finland	40	69	−
270	30	F	1	UK	50	28	−
271	57	F	1	Germany	43	37	37
272	64	F	1	Germany	42	56	56
273	53	M	1	Netherlands	41	42	42
274	52	M	1	Poland	43	46	−
275	57	F	1	Denmark	42	56	−
276	38	M	1	Poland	48	33	−
277	52	F	1	Poland	44	40	40
278	64	M	1	Poland	41	37	−
279	44	M	1	Italy	43	39	39
280	61	M	1	Germany	42	52	−
281	61	M	1	UK	42	44	44
282*	63	M	1	Spain	42	53	45
283	45	F	1	Poland	44	37	37
284	42	M	1	Italy	48	37	37
