# Supplementary material for: Exploring Genetic Factors Involved in Huntington Disease Age of Onset: E2F2 as a New Potential Modifier Gene
Source: PLoS One. 2015 Jul 6;10(7):e0131573. doi: 10.1371/journal.pone.0131573 (PMC4493078; doi:10.1371/journal.pone.0131573)
Supplement: S2 Table — Age: patient’s age at the time of blood collection; Sex: M (male) and F (female); CAGexp: CAG number repeat in expanded allele; Genotypes in E2F2 rs2742976 and Cq values in RT-qPCR analysis for the E2F2 and reference genes analyzed using SYBR Green and Taqman based assays are showed. NA: Not Analyzed. (PDF) [file pone.0131573.s005.pdf]

S2 Table. General information and expression analysis (RT-qPCR analysis) of the HD patients from Basque Hospitals. Age: patient's age at the time of blood collection; Sex: M (male) and F (female); CAGexp: CAG number repeat in expanded allele; Genotypes in *E2F2* rs2742976 and Cq values in RT-qPCR analysis for the *E2F2* and reference genes analyzed using SYBR Green and Taqman based assays are showed. NA: Not Analyzed.

Sample	Age	Sex	CAGexp	rs2742976 genotype	SYBR Green (Cq values)			Taqman (Cq values)		
					UBC	YWHAZ	E2F2	B2M	YWHAZ	E2F2
1	49	M	41	GT	21.24	21.66	25.32	21.84	23.90	32.55
2	34	M	42	GG	21.84	21.86	25.98	22.05	23.97	33.67
3	75	M	42	GG	NA	NA	NA	21.30	23.42	32.50
4	73	M	41	GG	26.46	26.37	29.05	22.21	23.88	32.95
5	45	M	41	GT	26.78	27.53	30.60	21.28	23.33	32.43
6	42	F	47	GG	NA	NA	NA	22.60	25.26	32.28
7	55	F	46	TT	21.79	21.64	27.07	22.29	24.23	33.13
8	55	F	47	TT	24.21	24.62	28.70	22.35	24.77	33.86
9	28	F	51	GG	NA	NA	NA	22.70	24.99	33.19
10	51	F	46	GG	25.36	25.77	28.26	22.42	24.06	32.75
11	33	M	48	GT	22.49	21.69	26.48	21.26	23.42	32.70
12	65	M	38	GT	21.25	21.56	26.48	22.21	24.16	33.36
13	60	M	36	GG	21.90	21.95	26.48	21.97	23.79	32.68
14	65	M	40	GT	30.37	29.60	35.73	23.29	25.26	34.26
15	77	F	39	TT	23.78	23.37	26.66	NA	NA	NA
16	80	F	39	GT	21.11	21.54	25.41	22.36	24.82	33.02
17	69	F	39	GT	22.75	22.32	26.96	22.37	24.39	32.35
18	51	M	44	GG	26.04	26.58	29.16	21.90	24.05	31.60
19	50	M	44	GG	27.73	27.90	30.61	22.50	24.69	31.47
20	50	M	44	GG	24.55	24.56	25.71	22.84	24.99	33.34
21	75	M	39	GT	24.40	23.60	27.01	21.61	24.21	31.23
22	41	F	45	GT	23.27	23.23	27.53	23.24	25.11	34.03
23	53	M	42	GG	25.30	24.29	28.10	23.50	25.12	33.02
24	83	M	39	GG	22.33	22.60	25.74	21.61	23.88	32.13
25	53	M	44	TT	28.36	28.79	33.23	21.52	23.91	32.33
26	61	F	41	GG	22.66	22.86	27.26	22.13	23.98	30.90
27	46	F	49	TT	27.54	27.84	33.66	22.12	24.14	32.92
28	50	F	42	GG	24.21	23.37	26.87	20.89	23.25	31.92
29	51	F	42	GT	22.77	22.31	26.39	21.48	23.54	32.34
30	48	M	43	GT	24.02	24.40	28.89	23.60	25.44	33.60
31	49	M	43	GT	23.82	22.79	27.60	NA	NA	NA
32	43	F	43	GG	24.70	24.22	29.86	NA	NA	NA
33	46	M	44	GT	26.25	26.32	29.72	21.50	23.33	31.07
34	41	M	46	GG	NA	NA	NA	21.20	23.47	31.78
35	43	F	43	GT	22.41	21.83	26.53	NA	NA	NA
