# Supplementary material for: Exploring Genetic Factors Involved in Huntington Disease Age of Onset: E2F2 as a New Potential Modifier Gene
Source: PLoS One. 2015 Jul 6;10(7):e0131573. doi: 10.1371/journal.pone.0131573 (PMC4493078; doi:10.1371/journal.pone.0131573)
Supplement: S3 Table — For each SNP, the genomic region (cytoband) and the closest gene is defined as well as the SNP type, chromosome location (according to genome assembly GRCh38), minor and major allele, HapMap CEU minor allele frequency (MAF), genotyping quality control (QC) notes and references if they have been previously studied in HD. * Indicates tag-SNPs. HWE: Hardy-Weinberg Equilibrium. (PDF) [file pone.0131573.s006.pdf]

S3 Table. SNPs genotyped in this study.

For each SNP, the genomic region (cytoband) and the closest gene is defined as well as the SNP type, chromosome location (according to genome assembly GRCh38), minor and major allele, HapMap CEU minor allele frequency (MAF), genotyping quality control (QC) notes and references if they have been previously studied in HD. * Indicates tag-SNPs. HWE: Hardy-Weinberg Equilibrium.

Cytoband	Gene	SNP ID	SNP type	Chromosome location	Minor/ major alleles	MAF (HapMap CEU)	Genotyping QC	Previously studied in HD
1p36.3	*DFFB*	rs6670527	Intronic*	3787749	C/G	0.433	Accepted	No
1p36.3	*DFFB*	rs7367066	nearGene-3*	3803755	T/C	0.257	Accepted	No
1p36	*E2F2*	rs2075993	UTR-3*	23836364	A/G	0.473	Removed (call rate <90%)	No
1p36	*E2F2*	rs2075995	exonic,missense*	23847464	C/A	0.483	Removed (call rate <90%)	No
1p36	*E2F2*	rs2742976	nearGene-5*	23858002	T/G	0.385	Accepted	No
2q33-q34	*CASP8*	rs1861270	Intronic*	202126615	A/G	0.358	Accepted	No
2q33-q34	*CASP8*	rs10931936	Intronic*	202143928	T/C	0.323	Accepted	No
2q33-q34	*CASP8*	rs13113	UTR-3	202152162	A/T	0.340	Removed (call rate <90%)	No
4p15.1	*PPARGC1*	rs2970882	nearGene-3*	23788341	C/T	0.339	Removed (call rate <90%)	No
4p15.1	*PPARGC1*	rs3755863	exonic, synonymous*	23815522	T/C	0.412	Accepted	Che *et al* ., 2011; Weydt *et al* ., 2009
4p15.1	*PPARGC1*	rs8192678	exonic,missense*	23815662	T/C	0.350	Accepted	Che *et al* ., 2011; Taherzadeh-Fard *et al.* , 2009; Weydt *et al* ., 2009
4p15.1	*PPARGC1*	rs6448228	Intronic*	23862142	G/A	0.481	Accepted	No
4p15.1	*PPARGC1*	rs2970871	Intronic*	23890582	A/G	0.434	Accepted	No
4p15.1	*CASP6*	rs5030606	nearGene-3*	110608967	A/G	0.307	Removed (HWE P<0.001)	No
4p15.1	*CASP6*	rs3181191	Intronic*	110612537	G/A	0.288	Accepted	No
4p15.1	*CASP6*	rs2301717	Intronic*	110619534	T/G	0.389	Genotyping failure	No
4p15.1	*CASP6*	rs5030539	Intronic*	110621820	A/G	0.393	Accepted	No
4p15.1	*CASP6*	rs3181187	Intronic*	110622910	A/G	0.389	Accepted	No
5q31	*TCERG1*	rs1991800	nearGene-5*	145753001	A/G	0.207	Accepted	No
5q31	*TCERG1*	rs2082407	nearGene-5*	145768952	T/C	0.425	Accepted	No
5q31	*TCERG1*	rs11747475	nearGene-5*	145784922	G/A	0.292	Accepted	No
5q31	*TCERG1*	rs11743333	Intronic*	145838440	G/A	0.367	Accepted	No
5q31	*TCERG1*	rs10068201	Intronic*	145849426	G/A	0.268	Accepted	No
5q31	*TCERG1*	rs962591	Intronic*	145879313	C/A	0.217	Removed (call rate <90%)	No
5q31	*TCERG1*	rs2241697	Intronic*	145887899	G/A	0.317	Accepted	No
5q31	*TCERG1*	rs3822506	UTR-3	145890455	T/C	0.106	Accepted	No
6q16.3	*GRIK2*	rs2518344	nearGene-5*	101775146	G/A	0.492	Genotyping failure	No
6q16.3	*GRIK2*	rs6930752	nearGene-5*	101839863	A/C	0.354	Accepted	No
6q16.3	*GRIK2*	rs3213607	exonic, synonymous	102483356	A/C	0.058	Accepted	No
6q16.3	*GRIK2*	rs2852612	Intronic*	102497857	C/A	0.425	Accepted	No
6q16.3	*GRIK2*	rs2852619	Intronic*	102513175	G/A	0.308	Accepted	No
6q16.3	*GRIK2*	rs2782901	nearGene-3*	102530252	C/T	0.389	Accepted	No
6q16.3	*GRIK2*	rs1232241	nearGene-3*	102595588	C/T	0.535	Accepted	No
6q16.3	*GRIK2*	rs3849205	nearGene-3*	102601177	T/C	0.420	Accepted	No
6q16.3	*SGK1*	rs9402571	nearGene-3	134488960	G/T	0.173	Accepted	No
6q16.3	*SGK1*	rs1057293	exonic,missense	134493397	A/G	0.093	Removed (call rate <90%)	No
6q16.3	*SGK1*	rs1743966	Intronic	134493947	G/A	0.181	Accepted	No
7q11.23	*HIP1*	rs1167801	exonic, synonymous	75176300	C/T	0.183	Accepted	No
7q11.23	*HIP1*	rs6962352	Intronic*	75189471	G/A	0.385	Removed (call rate <90%)	No
7q11.23	*HIP1*	rs794356	Intronic*	75196531	A/G	0.458	Removed (call rate <90%)	No
7q11.23	*HIP1*	rs237238	exonic, synonymous	75211414	G/A	0.068	Accepted	No
7q11.23	*HIP1*	rs237236	Intronic*	75212812	A/G	0.301	Accepted	No
7q11.23	*HIP1*	rs807874	Intronic	75222070	C/T	0.199	Removed (call rate <90%)	No
7q11.23	*HIP1*	rs2705788	Intronic*	75269163	A/T	0.500	Accepted	No
7q11.23	*HIP1*	rs2240133	Intronic*	75317218	T/C	0.383	Accepted	No

7q11.23	*HIP1*	rs2240134	Intronic*	75317281	T/C	0.308	Accepted	No
7q11.23	*HIP1*	rs6957776	Intronic*	75328671	C/G	0.438	Accepted	No
7q11.23	*HIP1*	rs12533075	Intronic*	75360839	G/A	0.314	Accepted	No
7q11.23	*HIP1*	rs4385416	nearGene-5*	75369213	G/A	0.350	Accepted	No
7q11.23	*HIP1*	rs7457874	nearGene-5*	75372875	A/G	0.250	Accepted	No
7q11.23	*HIP1*	rs6945301	nearGene-5*	75377439	G/A	0.487	Accepted	No
7q11.23	*HIP1*	rs4620231	nearGene-5*	75386669	C/A	0.212	Accepted	No
7q11.23	*HIP1*	rs9649625	nearGene-5*	75387937	A/C	0.323	Accepted	No
7q11.23	*HIP1*	rs12538253	nearGene-5*	75388031	C/T	0.302	Accepted	No
7q11.23	*HIP1*	rs10275942	nearGene-5*	75389831	C/T	0.281	Removed (call rate <90%)	No
7q11.23	*CCL26*	rs11465353	nearGene-3*	75398649	C/A	0.203	Removed (call rate <90%)	No
7q11.23	*CCL26*	rs2868166	Intronic*	75406913	A/C	0.414	Accepted	No
7q11.23	*CDK5*	rs9278	UTR-3	150750980	T/C	0.160	Removed (call rate <90%)	No
7q11.23	*CDK5*	rs2069459	Intronic*	150751590	A/C	0.388	Removed (call rate <90%)	No
7q11.23	*CDK5*	rs2069443	nearGene-5*	150755173	G/T	0.248	Accepted	No
10q21.3	*SIRT1*	rs17712705	nearGene-3*	69623271	A/G	0.237	Accepted	No
10q21.3	*SIRT1*	rs12778366	nearGene-5	69643079	C/T	0.128	Accepted	No
10q21.3	*SIRT1*	rs10997866	Intronic	69658651	A/G	0.246	Removed (call rate <90%)	No
10q21.3	*SIRT1*	rs10997870	Intronic*	69668014	G/T	0.288	Removed (call rate <90%)	No
10q21.3	*SIRT1*	rs7091896	Intronic*	69673245	C/G	0.242	Accepted	No
10q21.3	*SIRT1*	rs10997875	nearGene-3*	69679824	T/C	0.270	Accepted	No
11p13	*BDNF*	rs7124442	UTR-3*	27677041	C/T	0.327	Accepted	No
11p13	*BDNF*	rs6265	exonic,missense	27679916	A/G	0.195	Accepted	Alberch *et al* ., 2005; Metzger *et al* ., 2006
11p13	*BDNF*	rs11030121	Intronic*	27736207	T/C	0.358	Removed (call rate <90%)	No
12p13.1	*LINC01559*	rs1457624	nearGene-3*	13512740	A/C	0.417	Accepted	No
12p13.1	*LINC01559*	rs17821405	exonic,missense*	13526328	T/G	0.271	Removed (call rate <90%)	No
12p13.1	*LINC01559*	rs12423809	UTR-5*	13529594	C/A	0.342	Accepted	No
12p13.1	*LINC01559*	rs10845757	nearGene-5*	13539196	T/C	0.447	Accepted	No
12p13.1	*GRIN2B*	rs10845763	nearGene-3*	13560429	A/C	0.441	Accepted	No
12p13.1	*GRIN2B*	rs10744030	nearGene-3*	13577962	A/G	0.408	Accepted	No
12p13.1	*GRIN2B*	rs12814951	nearGene-3*	13700576	T/G	0.408	Accepted	No
12p13.1	*GRIN2B*	rs1806191	exonic, synonymous*	13716638	G/A	0.429	Accepted	Arning *et al* ., 2007
12p13.1	*GRIN2B*	rs1806201	exonic, synonymous*	13717508	A/G	0.212	Accepted	Arning *et al* ., 2005, 2007; Andresen *et al* ., 2007b; Saft *et al* ., 2011
12p13.1	*GRIN2B*	rs4764011	Intronic*	13720422	G/A	0.394	Accepted	Arning *et al* ., 2007
12p13.1	*GRIN2B*	rs1805539	Intronic*	13770190	C/G	0.358	Accepted	No
12p13.1	*GRIN2B*	rs2300235	Intronic*	13810845	G/A	0.442	Accepted	No
12p13.1	*GRIN2B*	rs2284406	Intronic	13825416	C/T	0.296	Genotyping failure	No
12p13.1	*GRIN2B*	rs2268115	Intronic*	13869725	G/T	0.411	Accepted	No
12p13.1	*GRIN2B*	rs2216128	Intronic*	13883014	G/A	0.230	Accepted	No
12p13.1	*GRIN2B*	rs220557	Intronic*	13947780	C/A	0.358	Accepted	No
12p13.1	*GRIN2B*	rs220567	Intronic*	13953403	C/G	0.372	Removed (call rate <90%)	No
12p13.1	*GRIN2B*	rs10845847	Intronic*	14020737	A/C	0.433	Removed (HWE P<0.001)	No
12p13.1	*GRIN2B*	rs12828473	Intronic*	14105728	A/G	0.457	Accepted	No
12p13.1	*GRIN2B*	rs10772722	nearGene-5*	14161665	T/G	0.442	Accepted	No
12p13.1	*GRIN2B-ATF7IP*	rs7966469	nearGene-5*	14289567	T/C	0.226	Accepted	No
12p13.1	*GRIN2B-ATF7IP*	rs7310659	nearGene-5*	14309749	A/G	0.305	Accepted	No
12p13.1	*GRIN2B-ATF7IP*	rs10845905	nearGene-5*	14342618	G/A	0.400	Accepted	No
12p13.1	*ATF7IP*	rs10845923	nearGene-5*	14368479	T/C	0.217	Accepted	No
12p13.1	*ATF7IP*	rs4341624	nearGene-5*	14371928	G/A	0.328	Accepted	No
12p13.1	*ATF7IP*	rs4764074	nearGene-5*	14428118	A/G	0.372	Accepted	No
12p13.1	*ATF7IP*	rs11055896	nearGene-5*	14433765	C/G	0.384	Accepted	No
12p13.1	*ATF7IP*	rs10845943	nearGene-5*	14437186	C/G	0.221	Accepted	No
12p13.1	*ATF7IP*	rs10744055	nearGene-5*	14459357	C/A	0.491	Removed (call rate <90%)	No
12p13.1	*ATF7IP*	rs10845987	Intronic*	14520241	T/C	0.491	Removed (call rate <90%)	No
12p13.1	*ATF7IP*	rs2231909	exonic,missense*	14577892	T/A	0.333	Accepted	No
12p13.1	*ATF7IP*	rs3213764	exonic,missense*	14587301	A/G	0.460	Accepted	No
12p13.1	*ATF7IP*	rs11055989	Intronic*	14622002	G/A	0.332	Accepted	No

12q13.1	*SP1*	rs7131938	nearGene-5*	53772927	T/C	0.212	Accepted	No
12q13.1	*SP1*	rs3741651	exonic, synonymous	53777171	G/A	0.168	Accepted	No
12q13.1	*SP1*	rs2694847	Intronic	53785028	G/A	0.175	Accepted	No
12q13.1	*SP1*	rs10876450	nearGene-3	53811034	C/T	0.168	Accepted	No
16p13.2	*GRIN2A*	rs8049651	exonic synonymous*	9943666	T/C	0.301	Accepted	No
16p13.2	*GRIN2A*	rs1969060	Intronic	10117137	G/A	0.167	Accepted	Arning *et al* ., 2005, 2007; Andresen *et al* ., 2007b; Saft *et al* ., 2011
16p13.2	*GRIN2A*	rs8049174	nearGene-5*	10293528	C/T	0.385	Accepted	No
16p13.2	*GRIN2A*	rs8044807	nearGene-5*	10358063	C/G	0.174	Accepted	No
16p13.2	*GRIN2A*	rs11646268	nearGene-5*	10498892	T/C	0.252	Accepted	No
17q21.2-q21.3	*HAP1*	rs7213337	UTR-3	39879428	T/C	0.106	Removed (call rate <90%)	No
17q21.2-q21.3	*HAP1*	rs11867808	Intronic*	39883672	A/G	0.372	Removed (call rate <90%)	No
17q21.2-q21.3	*HAP1*	rs35612698	exonic,missense	39884065	A/C	0.442	Accepted	No
17q21.2-q21.3	*HAP1*	rs4796693	exonic,missense	39884583	G/A	0.195	Removed (call rate <90%)	No
20q11.2	*E2F1*	rs3213183	nearGene-3*	32262962	A/G	0.292	Accepted	No
20q11.2	*E2F1*	rs3213142	nearGene-5	32274280	G/C	0.034	Removed (call rate <90%)	No
