# Supplementary material for: Exploring Genetic Factors Involved in Huntington Disease Age of Onset: E2F2 as a New Potential Modifier Gene
Source: PLoS One. 2015 Jul 6;10(7):e0131573. doi: 10.1371/journal.pone.0131573 (PMC4493078; doi:10.1371/journal.pone.0131573)
Supplement: S4 Table — (PDF) [file pone.0131573.s007.pdf]

S4 Table. Genotypes in the SNPs analyzed in each DNA sample from EHDN's collection.

GENE	DFFB	DFFB	E2F2	E2F2	E2F2	CASP8	CASP8	CASP8	PPARGC1	PPARGC1	PPARGC1	PPARGC1	PPARGC1
Sample/SNP	rs6670527	rs7367066	rs2075993	rs2075995	rs2742976	rs1861270	rs10931936	rs13113	rs2970882	rs3755863	rs8192678	rs6448228	rs2970871
1	C G	T T	A A	C C	G G	C C	C C	A A	C T	G G	G G	A G	G G
2	C G	C C	A G	A C	G G	C T	C T	A T	C T	G G	G G	A A	A A
3	C G	C T	A G	A C	G T	C T	C T	T T	T T	0 0	A A	A G	A G
4	G G	C C	A A	C C	G G	C T	C T	A T	C T	A G	A G	A A	A G
5	G G	C C	A G	A C	G T	C C	C C	A A	C T	A G	A G	G G	A A
6	C G	C T	A G	A C	G G	C T	C T	T T	C C	G G	G G	G G	A G
7	C G	C T	A G	A C	G G	C T	C T	T T	C T	A G	A G	A G	A A
8	C C	C T	A A	C C	G G	C T	C T	A T	T T	A A	A A	A A	0 0
9	C G	C T	A A	C C	G G	C T	C T	T T	C T	A G	A G	A G	A G
10	C G	T T	A A	C C	G T	C C	C C	A A	C T	A G	A G	A G	A G
11	C G	C C	G G	A A	T T	C C	C C	A A	T T	A A	A A	A G	A G
12	G G	0 0	0 0	C C	G G	C C	C C	A A	C T	G G	G G	A G	A A
13	C G	C C	A G	A A	G G	C C	C C	T T	C T	G G	G G	A G	A G
14	C G	C C	A G	A C	T T	C C	C C	A T	C T	A G	G G	A G	A G
15	C C	T T	A G	A C	G G	C C	C C	A A	T T	A G	A G	G G	A G
16	C G	C C	A G	A C	G T	C C	C C	A T	C T	A G	A G	A G	A G
17	C G	C T	A G	A C	G T	C C	C C	A A	C T	A G	A G	A G	G G
18	C G	C T	A G	A C	G G	C T	C T	T T	T T	G G	G G	A G	G G
19	G G	C C	G G	A A	T T	C C	C C	T T	T T	A A	A G	A A	A A
20	C C	C C	A G	A C	G G	T T	T T	T T	C T	A G	A G	A G	G G
21	G G	C C	A G	A C	G G	C C	C C	T T	C T	G G	G G	A G	G G
22	C G	C T	A A	C C	G T	C T	C T	A T	C T	G G	G G	A G	G G
23	C G	C C	A A	C C	G G	C C	C C	A A	C T	A G	G G	G G	G G
24	C G	C T	A G	A C	G G	C C	C C	A T	T T	G G	G G	A G	A G
25	C C	T T	A G	A C	T T	C T	C T	A T	C T	A G	A G	A G	G G
26	C G	C T	A A	C C	T T	C T	C T	T T	C T	A G	A G	A G	A A
27	G G	C C	G G	A A	G T	C C	C C	A T	T T	A A	A A	A G	G G
28	C G	C T	A G	A C	G T	C T	C T	A T	C T	G G	G G	G G	A G
29	C C	C C	A G	A C	G G	T T	T T	T T	C T	A G	A G	G G	G G
30	C G	C C	G G	A A	G G	C C	C C	A A	T T	A G	A G	A A	A G
31	G G	C C	G G	A A	G G	C C	C C	A T	T T	A G	A G	A A	A G
32	C C	C T	A G	A C	G G	C T	C T	T T	C T	A G	A G	A G	G G
33	C G	C C	G G	A A	G T	C C	C C	A T	T T	A A	A A	A A	A G
34	G G	C C	A A	C C	G G	C T	C T	A T	C T	A G	A G	A A	G G
35	C G	C C	A G	A C	G G	C C	C C	A A	C C	G G	G G	G G	A A

36	G G	C C	G G	A A	G T	C C	C C	A T	T T	0 0	A A	A A	A A
37	0 0	0 0	A G	A C	T T	C C	C C	A T	C T	A G	A G	A A	0 0
38	G G	C C	A G	A C	G G	C C	C C	T T	T T	A A	A A	A G	G G
39	0 0	0 0	A A	C C	G T	C C	C C	A A	T T	A G	A G	A G	0 0
40	C G	C T	G G	A A	G T	C T	C T	T T	T T	A A	A G	A G	G G
41	0 0	0 0	G G	A A	G T	C C	C C	A T	T T	A A	A A	A A	0 0
42	C G	C T	A A	C C	G T	C C	C C	T T	C T	G G	G G	A G	A G
43	0 0	0 0	A G	A C	G T	C C	C C	A A	C T	G G	G G	A A	0 0
44	G G	C T	A G	A C	G G	C T	C T	A T	T T	A G	G G	A G	A G
45	C C	0 0	G G	A A	G T	C C	C C	A T	C T	G G	G G	A A	0 0
46	C G	C C	G G	A A	T T	C C	C C	A A	T T	A G	A G	A G	G G
47	0 0	0 0	G G	A A	G G	C C	C C	T T	T T	A G	A G	A G	G G
48	C G	C C	A G	A C	G G	C C	C C	A T	T T	A A	A A	A A	A G
49	G G	C C	A G	A C	G G	T T	T T	T T	T T	A A	A A	G G	G G
50	0 0	0 0	A G	A C	G T	C C	C C	A A	T T	A G	A G	A A	0 0
51	G G	C C	G G	A A	G G	C C	C C	A A	C T	A G	A G	A G	A G
52	0 0	0 0	A G	A C	G G	C T	C T	A T	T T	0 0	A G	A A	0 0
53	C G	C T	G G	A A	G T	C C	C C	A A	C T	G G	G G	G G	A A
54	0 0	0 0	A G	A C	G T	T T	T T	T T	C T	A G	A G	A G	0 0
55	C C	C T	A A	C C	G T	C C	C C	T T	C C	G G	G G	A G	A G
56	C C	0 0	G G	A A	G G	C T	C T	A T	C T	A G	A G	A A	0 0
57	G G	C C	A A	C C	G T	C T	T T	T T	T T	A A	A A	A A	G G
58	G G	C C	0 0	A C	G T	C C	C C	A A	C C	G G	G G	A G	A A
59	C G	C T	G G	A A	G T	C C	C C	A A	T T	G G	G G	A G	A G
60	C G	C C	0 0	A C	G G	C C	C C	A A	C T	G G	G G	A A	G G
61	C G	C T	A G	A C	G T	C T	C T	A T	T T	A A	A A	A A	G G
62	0 0	0 0	A A	C C	G G	C C	C C	A A	C T	A G	A G	A G	G G
63	G G	C C	A G	A C	G G	C C	C C	A T	C C	G G	G G	G G	A A
64	C C	C C	A A	C C	T T	C C	0 0	A T	C T	0 0	G G	0 0	A G
65	C G	C T	A A	C C	G G	C C	C C	A A	T T	A A	A A	A A	A G
66	G G	C T	A A	C C	G G	C C	0 0	A T	T T	0 0	A G	A G	G G
67	C G	C C	G G	A C	G G	T T	T T	T T	C T	A G	A G	A G	A G
68	C G	C C	G G	A A	G T	C C	C C	A T	T T	A G	A G	A G	G G
69	C G	C T	G G	A A	G T	C C	C T	T T	C T	G G	G G	A A	A A
70	C C	T T	A G	A C	G T	C C	C C	0 0	C T	0 0	0 0	A A	A A
71	G G	C C	A A	C C	G T	C C	C C	A T	C T	A G	A G	A G	G G
72	G G	C C	G G	A A	G G	C C	C C	T T	C T	A G	A G	A G	A G
73	C G	C T	A G	A C	G G	C C	C C	A A	T T	A G	A G	A G	A A
74	C G	C C	A G	A C	T T	C T	C T	A T	T T	A G	A G	A G	A G
75	C G	C T	A G	A C	G T	C T	C T	A T	C T	A G	A G	G G	G G
76	C G	C C	A G	A C	G T	C T	C T	T T	C T	A G	A G	A G	A A
77	G G	C C	A G	A A	G T	C C	C C	A T	T T	A G	A G	A G	A G

78	C G	C C	G G	A A	G T	T T	T T	T T	T T	A G	A G	A G	A G
79	G G	C C	A A	C C	G T	C C	C C	A T	T T	A G	A G	A A	A G
80	C G	C T	G G	A A	G G	C T	C T	A T	C T	G G	G G	A G	A G
81	C G	C T	G G	A A	G T	C C	C C	A T	T T	A A	A A	A G	A G
82	C G	C C	A G	A C	G T	C T	T T	T T	C C	G G	G G	A A	A G
83	C C	C T	A G	A C	G G	C C	C C	A T	T T	A G	A G	A G	G G
84	G G	C C	A A	C C	G G	T T	T T	T T	C T	A G	A G	A G	G G
85	C G	C T	A G	A C	G T	C C	C C	A A	C T	G G	G G	A G	G G
86	G G	C C	G G	A A	G T	C C	C C	A T	C C	G G	G G	A G	A A
87	G G	C C	A A	C C	G T	C T	C T	T T	T T	G G	G G	A G	A G
88	C C	C C	A G	A C	G G	C C	C C	A A	C T	A G	A G	A G	A G
89	0 0	C T	A G	A C	0 0	0 0	0 0	0 0	0 0	0 0	0 0	0 0	A A
90	G G	C C	A A	C C	G G	C T	C T	A T	C T	A G	G G	A A	A G
91	G G	C C	A A	C C	G G	C C	C C	A T	T T	A G	A G	A G	A G
92	G G	C C	G G	A A	G T	T T	T T	T T	C C	G G	G G	A G	A A
93	G G	C C	A G	A C	G G	C T	C T	A T	T T	A G	A G	A A	A A
94	C G	C T	G G	A A	G G	C C	C C	A A	C C	G G	G G	A A	A A
95	0 0	T T	G G	A A	G G	C T	C T	T T	C T	A G	A G	A A	A G
96	G G	C C	A A	C C	G G	C T	C T	A T	T T	A A	A G	A G	G G
97	C G	C T	A G	A C	G G	C C	C C	A T	C T	G G	G G	A G	A G
98	C G	C T	A G	A C	G T	C C	C C	A T	C T	G G	G G	A A	A G
99	C C	T T	A A	C C	G T	C C	C C	A T	C T	A G	A G	A A	A G
100	0 0	0 0	0 0	A C	0 0	C C	C C	A A	C T	G G	G G	A G	0 0
101	G G	C C	A A	C C	G G	C T	C T	T T	C T	G G	G G	A A	A G
102	C G	C C	A A	C C	G G	T T	T T	T T	C T	G G	G G	A G	G G
103	C G	C T	A G	C C	G G	C C	C C	A T	T T	A A	A A	A A	A G
104	C G	C C	G G	A A	G T	C C	C C	T T	C C	G G	G G	A G	A G
105	C G	C C	A G	A C	G T	C C	C C	A A	C T	A G	A G	A G	G G
106	G G	C C	A A	C C	G G	C C	C C	A A	T T	A A	A A	A A	A A
107	C G	C C	G G	A A	G G	C C	C C	0 0	C T	A G	A G	A A	A A
108	C G	C T	G G	A A	G T	C C	C C	A T	C T	A G	A G	A G	G G
109	C C	T T	A G	A C	G T	C T	C T	T T	C C	G G	G G	A G	G G
110	G G	C C	A G	A C	T T	C C	C C	A T	C T	A G	A G	A G	G G
111	G G	C C	G G	A A	T T	C C	C C	A T	C T	A G	A G	A G	G G
112	C G	C T	A G	0 0	G G	C T	C T	A T	C T	G G	G G	A A	A G
113	C G	C C	A G	A C	G G	C T	C T	A T	C T	A G	A G	A A	A G
114	G G	C C	G G	A A	G G	C T	C T	T T	C T	G G	G G	A G	A G
115	G G	C C	G G	A A	G T	C C	C C	A T	T T	G G	G G	A G	A G
116	C G	C T	A G	A C	G T	C T	T T	T T	T T	A G	A G	A A	A A
117	C G	C T	A G	A C	G G	C C	C C	A A	C C	G G	G G	A A	A G
118	G G	C C	A G	A C	G T	C C	C C	A T	C C	G G	G G	G G	A G
119	C G	C T	G G	A A	T T	C C	C C	A T	C C	G G	G G	A A	A A

120	G G	C C	A G	C C	G T	C T	C T	A T	C T	A G	A G	A G	A G
121	C G	C T	G G	A A	T T	C C	C C	A T	C T	G G	G G	A G	G G
122	G G	C C	G G	A A	G T	C C	C C	A T	C T	G G	G G	A G	A G
123	G G	C C	G G	A A	T T	C C	C C	A A	T T	G G	G G	A G	A G
124	G G	C C	A G	A C	G T	C C	C T	T T	C T	A G	A G	A G	G G
125	0 0	C C	A G	A C	T T	C C	C C	A T	C T	G G	G G	A A	0 0
126	C G	C T	A G	A C	G T	C T	C T	T T	T T	G G	G G	A G	A G
127	C G	C T	A A	C C	G G	C C	C C	A T	T T	A A	A A	A A	A G
128	G G	C C	A G	A C	G G	C C	C C	A A	C C	G G	G G	A A	A A
129	0 0	C T	A G	A C	G T	C C	C C	T T	0 0	0 0	A A	A G	0 0
130	C G	C T	A G	A C	G G	C T	C T	A T	C T	A G	G G	A G	A G
131	0 0	C T	A A	C C	G G	C C	C C	A A	T T	A A	A A	A G	0 0
132	C G	C T	A G	A C	G G	C C	C C	A T	C T	A G	A G	A G	A G
133	C C	T T	0 0	0 0	0 0	C T	0 0	A T	0 0	0 0	A G	G G	A A
134	C C	T T	A G	A C	G T	C C	C C	A A	C T	G G	G G	G G	A G
135	0 0	C T	G G	A A	G T	C T	C T	A T	T T	A A	A G	A G	0 0
136	C C	C T	A G	C C	G G	C T	C T	A T	T T	A G	A G	A A	A G
137	C C	C T	A G	A A	G T	C C	C C	A A	C T	A G	A G	A A	A G
138	C G	C C	G G	A A	T T	C C	C C	T T	C T	A G	A G	A A	A G
139	G G	C C	A G	A C	G T	C C	C C	A T	C T	A G	A G	A A	A G
140	C G	C T	A G	A C	T T	C T	0 0	A T	C T	G G	G G	G G	A A
141	G G	C C	G G	A A	G G	C T	C T	T T	C T	A G	A G	A G	A G
142	C G	C T	G G	A A	G G	C C	C C	A T	T T	A A	A A	A G	A G
143	C G	C C	G G	A A	T T	C T	C T	A T	C T	G G	G G	G G	G G
144	C C	C T	A G	A C	G T	C T	C T	T T	C T	G G	G G	A A	A A
145	G G	C C	G G	A A	G T	C T	C T	A T	C T	G G	G G	A G	A G
146	C C	C T	G G	A A	G G	C T	C T	A T	T T	G G	G G	A G	G G
147	C C	C T	0 0	0 0	0 0	C C	C C	T T	T T	A G	A G	A A	A G
148	C C	T T	G G	A A	G T	C C	C C	T T	C T	G G	G G	A G	A G
149	G G	C C	A G	A C	G G	C T	C T	A T	T T	G G	G G	A G	A G
150	0 0	C T	A G	A C	G G	C T	C T	A T	C T	A G	A G	A G	0 0
151	G G	C C	G G	A A	T T	C T	C T	T T	C T	G G	G G	G G	A A
152	C G	C T	A G	A C	G G	C C	C C	A A	C T	A G	A G	A G	A G
153	C G	T T	A G	A C	T T	C T	C T	A T	C C	G G	G G	A G	A G
154	G G	C C	G G	A A	G G	C T	0 0	T T	0 0	0 0	G G	A G	G G
155	C G	C T	A A	C C	G G	C T	C T	T T	T T	A G	G G	A G	G G
156	C G	C T	G G	A A	G G	T T	T T	T T	C C	G G	G G	A A	A A
157	C C	C C	A G	A C	G T	C C	C C	A A	C T	A G	G G	G G	G G
158	C C	C C	G G	A A	0 0	C C	C C	A T	0 0	0 0	A G	A A	A G
159	C C	C T	A A	C C	G G	C T	C T	A T	C T	A A	A A	A G	G G
160	0 0	0 0	A A	C C	G G	C T	0 0	A T	0 0	0 0	G G	A A	0 0
161	G G	C C	A G	A C	G G	C C	C C	A A	T T	A G	G G	G G	G G

162	C G	C T	A A	C C	G G	C C	C C	A T	C T	G G	G G	A A	A G
163	C C	C T	A G	A C	G T	T T	T T	T T	C T	G G	G G	A A	A A
164	C G	C T	A G	A C	G T	C T	C T	A T	C C	G G	G G	A G	A G
165	C G	C T	A G	A C	T T	C C	C C	A A	C T	A G	A G	A G	A G
166	C C	T T	G G	A A	T T	C C	C C	A T	C T	G G	G G	A G	G G
167	G G	C T	A G	A C	G T	C C	C C	A T	C T	A G	A G	A G	G G
168	G G	C C	A G	A C	G T	C T	C T	A T	C C	A G	A G	A A	A G
169	G G	C C	A G	A C	G G	C C	C C	T T	C T	G G	G G	A G	A G
170	C G	C T	G G	A C	G G	C C	C C	A T	T T	A G	A G	A G	A A
171	C G	C T	A G	A C	G T	T T	T T	T T	C T	A G	A G	A G	A G
172	C C	T T	A G	A C	G G	0 0	0 0	0 0	0 0	0 0	0 0	0 0	0 0
173	G G	C C	A A	C C	G G	C C	C C	A T	C C	G G	G G	A G	G G
174	C G	C T	A G	A C	G T	C C	C C	A A	C T	G G	G G	A G	A G
175	C G	C C	A A	C C	G G	C T	C T	T T	C C	G G	G G	A G	A G
176	G G	C C	A G	A C	G G	C T	C T	T T	C T	A A	A A	A A	A G
177	G G	C C	0 0	0 0	G T	C C	C C	0 0	0 0	G G	G G	A A	A A
178	C G	C T	0 0	0 0	G T	C C	C T	0 0	0 0	A A	A A	A A	G G
179	G G	C C	0 0	0 0	G T	C T	C T	0 0	0 0	0 0	G G	A G	A G
180	G G	C C	0 0	0 0	G T	C C	C C	0 0	0 0	G G	G G	A G	A A
181	G G	C T	0 0	0 0	G G	C T	C T	0 0	0 0	0 0	0 0	G G	A G
182	C G	C T	0 0	0 0	G T	C T	C T	0 0	0 0	A G	A A	G G	A G
183	G G	C T	0 0	0 0	G T	C C	C C	0 0	0 0	A A	A A	A A	A G
184	G G	C C	0 0	0 0	G T	C T	C T	0 0	0 0	A G	0 0	A G	G G
185	C G	C T	0 0	0 0	0 0	C C	C C	0 0	0 0	0 0	0 0	A A	A A
186	0 0	C C	0 0	0 0	G T	C C	C C	0 0	0 0	G G	0 0	A G	A G
187	C G	C T	0 0	0 0	G G	C C	C C	0 0	0 0	0 0	A A	A A	A G
188	G G	C T	0 0	0 0	G G	C C	C C	0 0	0 0	0 0	0 0	A G	A A
189	C G	C C	0 0	0 0	G G	C T	C T	0 0	0 0	0 0	G G	A A	G G
190	G G	C C	0 0	0 0	G G	C T	C T	0 0	0 0	A G	A G	A A	A G
191	C G	C T	0 0	0 0	G G	C C	C C	0 0	0 0	G G	G G	G G	A G
192	G G	C C	0 0	0 0	G G	C T	C T	0 0	0 0	A G	G G	G G	A G
193	C G	C T	0 0	0 0	T T	C C	C C	0 0	0 0	A G	A G	G G	A G
194	C G	T T	0 0	0 0	G G	C T	C T	0 0	0 0	A A	A A	G G	A G
195	G G	C C	0 0	0 0	G T	T T	T T	0 0	0 0	G G	G G	A G	A G
196	G G	C C	0 0	0 0	T T	C C	C T	0 0	0 0	G G	G G	A A	A A
197	G G	C C	0 0	0 0	G T	C T	C T	0 0	0 0	G G	0 0	A G	A G
198	G G	C C	0 0	0 0	G T	C T	C T	0 0	0 0	A A	A A	A G	A G
199	C C	T T	0 0	0 0	G T	C T	C T	0 0	0 0	0 0	0 0	A A	G G
200	C G	C C	0 0	0 0	G G	C C	C C	0 0	0 0	A G	A A	A G	G G
201	G G	C C	0 0	0 0	T T	C C	C C	0 0	0 0	A A	A G	A G	G G
202	C G	C T	0 0	0 0	T T	C C	C C	0 0	0 0	G G	G G	A A	A G
203	C G	C C	0 0	0 0	G G	C C	C C	0 0	0 0	G G	0 0	A G	G G

204	C C	C T	0 0	0 0	G G	C C	C C	0 0	0 0	G G	G G	A A	0 0
205	C G	C T	0 0	0 0	G G	C C	C C	0 0	0 0	A G	A A	A G	G G
206	C G	C C	0 0	0 0	G G	C C	C C	0 0	0 0	A A	A A	A G	A G
207	G G	C C	0 0	0 0	T T	C T	C T	0 0	0 0	A G	A A	G G	A G
208	0 0	C C	0 0	0 0	G G	C C	C C	0 0	0 0	A G	A A	A G	0 0
209	G G	C C	0 0	0 0	G G	C C	C C	0 0	0 0	A G	A A	A G	A A
210	0 0	C C	0 0	0 0	G G	C C	C C	0 0	0 0	G G	0 0	A G	A G
211	C G	C T	0 0	0 0	G T	C C	C C	0 0	0 0	A A	A A	A A	A A
212	C G	C C	0 0	0 0	G T	C C	C C	0 0	0 0	A A	A A	A G	G G
213	G G	C C	0 0	0 0	G T	C C	C C	0 0	0 0	A A	A A	A G	A G
214	C C	T T	0 0	0 0	T T	C C	C C	0 0	0 0	A G	A G	A G	A G
215	C G	C T	0 0	0 0	T T	C T	C T	0 0	0 0	A G	A G	A A	A A
216	C G	C T	0 0	0 0	G T	C C	C C	0 0	0 0	A A	A G	A G	G G
217	C G	C T	0 0	0 0	T T	T T	T T	0 0	0 0	A A	A G	A A	A A
218	C G	C C	0 0	0 0	G G	C T	C T	0 0	0 0	G G	G G	A G	A G
219	G G	C C	0 0	0 0	T T	C T	C T	0 0	0 0	A G	A G	A G	A A
220	G G	C C	0 0	0 0	T T	C C	C C	0 0	0 0	G G	G G	G G	G G
221	C C	C T	0 0	0 0	G G	C C	C C	0 0	0 0	A G	0 0	A G	G G
222	G G	C C	0 0	0 0	G T	C T	C T	0 0	0 0	A G	A A	A G	A A
223	G G	C C	0 0	0 0	G G	C T	C T	0 0	0 0	A G	A G	A G	A A
224	G G	C C	0 0	0 0	G G	C C	C C	0 0	0 0	0 0	G G	A A	G G
225	C G	C T	0 0	0 0	G G	C C	C T	0 0	0 0	A A	A A	A A	A A
226	C G	C C	0 0	0 0	T T	T T	T T	0 0	0 0	G G	G G	G G	G G
227	C C	C T	0 0	0 0	G T	C T	C T	0 0	0 0	A G	A G	A A	G G
228	G G	C C	0 0	0 0	T T	C C	C C	0 0	0 0	A G	A G	A G	G G
229	C G	C T	0 0	0 0	G T	C C	C C	0 0	0 0	A G	A G	G G	A G
230	C G	C T	0 0	0 0	T T	C C	C C	0 0	0 0	A A	A A	A A	0 0
231	C G	C T	0 0	0 0	G T	T T	T T	0 0	0 0	A G	A G	A G	A G
232	0 0	C C	0 0	0 0	G T	C T	C T	0 0	0 0	A G	0 0	G G	A G
233	C G	C T	0 0	0 0	G T	C C	C C	0 0	0 0	A A	A A	A G	A G
234	C C	C T	0 0	0 0	G G	C T	T T	0 0	0 0	G G	0 0	A G	A A
235	C G	C C	0 0	0 0	G G	C C	C C	0 0	0 0	A G	A G	A G	A G
236	C G	C T	0 0	0 0	G T	C C	C C	0 0	0 0	A G	A G	G G	G G
237	C G	C T	0 0	0 0	G T	T T	T T	0 0	0 0	G G	G G	0 0	G G
238	C G	C C	0 0	0 0	G T	C T	C T	0 0	0 0	A G	A G	A A	A A
239	C C	C C	0 0	0 0	G G	C T	C T	0 0	0 0	A G	A G	A G	A G
240	C C	T T	0 0	0 0	G G	C C	C C	0 0	0 0	A G	A G	A G	A G
241	G G	C T	0 0	0 0	G G	C T	C T	0 0	0 0	A G	A A	A G	A A
242	G G	C T	0 0	0 0	G G	C C	C T	0 0	0 0	A G	A A	A G	A G
243	G G	C C	0 0	0 0	G T	C T	C T	0 0	0 0	A G	A G	A G	A G
244	G G	C C	0 0	0 0	T T	C C	C C	0 0	0 0	G G	0 0	A G	A G
245	G G	C C	0 0	0 0	G T	C C	C C	0 0	0 0	A G	A G	A G	G G

246	G G	C C	0 0	0 0	G G	C T	C T	0 0	0 0	A A	A A	A G	A G
247	C G	C C	0 0	0 0	G T	C C	C T	0 0	0 0	A A	A A	A A	G G
248	G G	C C	0 0	0 0	G T	C C	C C	0 0	0 0	A G	0 0	A A	A G
249	C G	C T	0 0	0 0	G G	C C	C C	0 0	0 0	0 0	A G	A A	G G
250	C G	C T	0 0	0 0	G G	C C	C T	0 0	0 0	A G	G G	A A	A G
251	C G	C T	0 0	0 0	T T	C C	C C	0 0	0 0	A G	A G	A A	A G
252	C G	C C	0 0	0 0	G T	C C	C C	0 0	0 0	G G	G G	A G	A A
253	C G	C C	0 0	0 0	G G	T T	T T	0 0	0 0	G G	G G	A G	A G
254	C C	C C	0 0	0 0	G G	C C	C C	0 0	0 0	A G	A G	A G	A G
255	G G	C C	0 0	0 0	G T	C T	C T	0 0	0 0	A G	A A	A A	A G
256	C G	C C	0 0	0 0	G T	C C	C C	0 0	0 0	A G	A A	A A	G G
257	C C	C T	0 0	0 0	G T	C T	C T	0 0	0 0	A G	0 0	A G	A A
258	C C	C C	0 0	0 0	G G	C T	C T	0 0	0 0	A G	A G	A G	G G
259	C G	C T	0 0	0 0	G T	C C	C C	0 0	0 0	A G	A G	A G	G G
260	C G	C T	0 0	0 0	G T	C C	C C	0 0	0 0	G G	G G	G G	A G
261	G G	C C	0 0	0 0	G G	0 0	0 0	0 0	0 0	0 0	0 0	0 0	A G
262	C G	C T	0 0	0 0	G G	C T	C T	0 0	0 0	A A	A A	A A	G G
263	C G	C T	0 0	0 0	G T	C T	C T	0 0	0 0	G G	G G	A A	A A
264	G G	C C	0 0	0 0	G G	C C	C C	0 0	0 0	G G	G G	G G	A G
265	C G	C C	0 0	C C	T T	C T	C T	T T	C T	G G	G G	A A	A G
266	G G	C C	0 0	C C	G G	C C	C C	A T	T T	A A	A G	A G	G G
267	C G	C C	0 0	A A	G G	T T	T T	T T	T T	A A	A A	A A	G G
268	C G	C C	0 0	A A	G T	T T	T T	T T	C T	G G	G G	A A	A G
269	C C	C T	0 0	A A	T T	C C	C C	A T	C T	G G	G G	A G	A G
270	C C	C T	0 0	A C	G G	C T	C T	A T	C T	A G	A G	G G	A G
271	G G	C C	0 0	C C	G T	C T	C T	T T	C C	G G	G G	G G	A A
272	C G	C T	0 0	A C	G G	C T	C T	A T	T T	A G	G G	G G	A G
273	C G	C C	0 0	A C	G G	C C	C C	A T	C C	G G	G G	G G	A A
274	G G	C C	0 0	A A	G T	C T	C T	A T	T T	A G	A G	A G	A G
275	G G	C C	0 0	C C	G G	C T	C T	0 0	C T	0 0	A G	A G	A G
276	G G	C C	0 0	A A	T T	0 0	0 0	0 0	0 0	0 0	0 0	0 0	A G
277	G G	C T	0 0	A C	G T	C T	C T	T T	C T	G G	G G	A G	A G
278	C C	T T	0 0	A A	G G	C C	C C	A A	T T	G G	G G	A A	G G
279	G G	C C	0 0	A A	G G	C C	C C	A T	C T	A G	A G	A G	G G
280	C G	C C	0 0	C C	G G	C T	C T	A T	T T	G G	G G	A G	A G
281	C G	C C	0 0	A C	G G	C T	C T	T T	C T	A G	A G	A G	G G
282	G G	C C	0 0	A A	G T	C C	C C	T T	C T	A G	A G	A A	A G
283	G G	C T	0 0	C C	G T	C C	C C	A T	C T	G G	G G	A G	A G
284	G G	C C	0 0	A C	G G	C C	C C	A A	T T	A G	A G	G G	G G

GENE	CASP6	CASP6	CASP6	CASP6	CASP6	TCERG1	TCERG1	TCERG1	TCERG1	TCERG1	TCERG1	TCERG1	TCERG1
SNP/Sample	rs5030606	rs3181191	rs2301717	rs5030539	rs3181187	rs1991800	rs2082407	rs11747475	rs11743333	rs10068201	rs962591	rs2241697	rs3822506
1	C T	T T	0 0	C C	G G	C C	T T	A G	A G	A G	A C	A G	C C
2	T T	T T	0 0	C T	A G	C C	C C	A A	A A	A A	A A	G G	C C
3	T T	T T	0 0	C T	A G	C C	C C	A A	A G	A G	A C	A G	C T
4	C T	T T	0 0	C C	G G	C C	C T	A A	A A	A A	A A	G G	C C
5	C C	C C	0 0	C T	A G	C C	C C	A A	A A	A A	A A	G G	C C
6	C C	C T	0 0	C T	A G	C C	C C	A A	A A	A A	A A	G G	C C
7	C T	C T	0 0	C T	A G	0 0	C C	A A	A G	A G	A C	A G	C C
8	T T	T T	0 0	C C	G G	0 0	C T	A A	A G	A G	A C	A G	C C
9	T T	T T	0 0	C C	G G	C C	C C	A A	A G	A G	A C	A G	C C
10	C T	T T	0 0	C C	A G	C C	C C	A A	A G	A A	A A	A G	C C
11	T T	T T	0 0	C C	G G	C T	C T	A G	A G	A G	A C	A G	C T
12	T T	T T	0 0	C T	A G	C C	C C	A A	A A	A A	0 0	G G	C C
13	T T	T T	0 0	C C	G G	C T	C T	A A	A A	A A	A A	G G	C C
14	C T	T T	0 0	C C	G G	C C	C C	A A	A G	A G	A C	A G	C T
15	C T	C T	0 0	C T	A G	C C	C C	A A	A A	A A	A A	G G	C C
16	C C	C C	0 0	T T	A A	C T	T T	A G	A A	A A	A A	G G	C C
17	T T	T T	0 0	C C	G G	T T	T T	A G	A G	A G	A C	A G	C C
18	C T	T T	0 0	C T	A G	C C	C C	A A	A G	A A	A A	A G	C C
19	T T	T T	0 0	C C	G G	0 0	C T	A G	A G	A G	A C	A G	C C
20	T T	T T	0 0	C C	G G	0 0	T T	G G	G G	G G	C C	A A	C C
21	T T	T T	0 0	C C	G G	C T	C T	A G	G G	G G	C C	A A	C T
22	C T	C T	0 0	C T	A G	C C	C T	A A	A A	A A	A A	G G	C C
23	T T	T T	0 0	C T	A G	C C	C C	A A	A G	A G	A C	A G	C T
24	C T	T T	0 0	C C	G G	C T	C T	A G	A A	A A	A A	G G	C C
25	C T	C T	0 0	C T	A G	C T	C T	A G	A G	A G	A C	A G	C T
26	C T	C T	0 0	C T	A G	C C	C C	A A	A G	A G	A C	A G	C T
27	C C	C T	0 0	C T	A G	C C	C T	A G	G G	A G	A C	A A	C T
28	T T	T T	0 0	C C	G G	C T	C T	A G	A A	A A	A A	G G	C C
29	C C	C C	0 0	T T	A A	C C	C T	A G	A G	A A	A A	A G	C C
30	T T	T T	0 0	C C	G G	C C	C T	A G	A G	A G	A C	A G	C T

31	C T	C T	0 0	C T	A G	0 0	C T	A G	A G	A G	A C	A G	C C
32	T T	T T	0 0	C C	G G	0 0	C C	A A	A G	A G	A C	A G	C T
33	C T	C T	0 0	C T	A G	C C	C C	A A	G G	A G	A C	A A	C T
34	C C	C C	0 0	C T	A G	C C	C C	A A	A A	A A	A A	G G	C C
35	C C	C C	0 0	T T	A A	C C	C C	A A	A A	A A	A A	G G	C C
36	C T	C T	0 0	C T	A G	C C	C C	A A	G G	G G	C C	A A	C T
37	C T	C T	0 0	C T	A G	C C	C C	A A	A G	0 0	0 0	A G	C T
38	C C	C C	0 0	T T	A A	C C	C T	A G	A A	A A	A A	G G	C C
39	C C	C T	0 0	C T	A G	0 0	C T	0 0	A A	0 0	0 0	G G	C C
40	T T	T T	0 0	C C	G G	C C	C C	A A	A A	A A	A A	G G	C C
41	C T	C T	0 0	C T	A G	0 0	C C	0 0	A A	0 0	0 0	G G	C C
42	T T	T T	0 0	C C	G G	C T	T T	A G	A G	A A	A A	A G	C C
43	C T	C T	0 0	C T	A G	0 0	C C	0 0	A A	0 0	0 0	G G	C C
44	C C	C C	0 0	T T	A A	C C	T T	G G	A A	A A	A A	G G	C C
45	C T	C T	0 0	C T	A G	C C	C T	A G	A G	A A	A A	A G	C C
46	T T	T T	0 0	C C	G G	C C	C C	A A	A A	A A	A A	G G	C C
47	C T	C T	0 0	C T	A G	C C	C C	A A	A G	A A	A A	G G	C C
48	C C	C C	0 0	C T	A G	C C	T T	A G	A G	A G	A C	A G	C T
49	T T	T T	0 0	C C	G G	T T	T T	A A	G G	G G	C C	A A	C C
50	T T	T T	0 0	C C	G G	0 0	C C	0 0	G G	0 0	0 0	A A	C T
51	T T	T T	0 0	C C	G G	C C	C C	A A	A A	A A	A A	G G	C C
52	T T	T T	0 0	C C	G G	0 0	C C	0 0	G G	0 0	0 0	A A	C T
53	C T	T T	0 0	C C	G G	C C	C C	A A	A G	A A	A A	A G	C C
54	C T	C T	0 0	C T	A G	C C	C C	A A	A G	0 0	0 0	A G	C C
55	C C	C C	0 0	T T	A A	0 0	T T	G G	A G	A G	A C	A G	C C
56	C T	C T	0 0	C T	A G	0 0	T T	A A	A A	0 0	A A	G G	C C
57	C T	C T	0 0	C T	A G	C T	C T	A A	A G	A A	A A	A G	C C
58	T T	T T	0 0	C C	G G	C T	T T	A A	A A	A A	A A	G G	C C
59	C C	C T	0 0	C T	A G	C C	C T	A G	A A	A A	A A	G G	C C
60	C T	C T	0 0	C T	A G	C T	C T	A G	A A	A A	A A	G G	C C
61	C T	C T	0 0	T T	A A	C C	C T	A G	A G	A G	A C	A G	C C
62	T T	T T	0 0	C T	A G	C C	C C	A A	A G	0 0	0 0	A G	C T
63	C T	C T	0 0	C T	A G	T T	T T	G G	G G	A G	A C	A A	C C

64	T T	T T	0 0	0 0	0 0	C C	C C	A A	A G	A G	A C	0 0	C T
65	C T	T T	0 0	C C	G G	C C	C C	A A	A G	A G	A C	A G	C C
66	T T	T T	0 0	C T	G G	C C	C C	A A	G G	G G	0 0	0 0	C C
67	T T	T T	0 0	C C	G G	0 0	C C	A A	A G	A G	A C	A G	C C
68	C T	C T	0 0	C T	A G	0 0	C C	A A	A G	A G	A C	A G	C T
69	C C	C C	0 0	T T	A A	C T	C T	A G	A G	A G	A C	A G	C C
70	T T	T T	0 0	C C	G G	C C	C C	A A	A G	A G	A C	A G	C C
71	C T	C T	0 0	C T	A G	C C	T T	G G	A G	A G	A C	A G	C C
72	C T	C T	0 0	C T	A G	C T	T T	G G	A G	A G	A C	A G	C C
73	C T	C T	0 0	T T	A A	C C	C T	A A	A A	A A	A A	G G	C C
74	C T	C T	0 0	C T	A G	C T	C T	A G	A G	A G	A C	A G	C C
75	C T	C T	0 0	C T	A G	C T	C T	A G	A A	A A	A A	G G	C C
76	C C	C T	0 0	C T	A G	C C	C C	A A	A G	A G	A C	A G	C C
77	C T	C T	0 0	C T	A G	C C	C C	A A	A A	A A	A A	G G	C C
78	C C	C C	0 0	T T	A A	C T	C T	A A	A A	A A	A A	G G	C C
79	C T	C T	0 0	C T	A G	0 0	C C	A A	A A	A A	A A	G G	C C
80	T T	T T	0 0	C T	A G	0 0	C C	A A	A A	A A	A A	G G	C C
81	C C	C C	0 0	T T	A A	C T	C T	A A	A A	A A	A A	G G	C C
82	T T	T T	0 0	C C	G G	C C	C C	A A	A G	A G	A C	A G	C C
83	C C	C C	0 0	T T	A A	C T	C T	A G	G G	G G	C C	A A	C T
84	C T	C T	0 0	C T	A G	C C	C C	A A	G G	G G	C C	A A	C T
85	C T	C T	0 0	C T	A G	C C	C C	A A	A G	A G	A C	A G	C C
86	C C	C C	0 0	T T	A A	C C	C C	A A	A G	A G	A C	A G	C C
87	C T	C T	0 0	C T	A G	C C	C C	A A	A A	A A	A A	G G	C C
88	TT	TT	0 0	CC	GG	0 0	CC	AA	AG	AG	AC	AG	CT
89	0 0	C T	0 0	0 0	0 0	C C	0 0	A A	0 0	A A	A A	0 0	C T
90	C C	T T	0 0	C C	G G	C C	C T	G G	A G	A G	A C	A G	C T
91	C T	C T	0 0	C T	A G	C C	C C	A A	A G	A G	A C	A G	C C
92	C T	C T	0 0	T T	A A	C C	C C	A A	A G	A G	A C	A G	C T
93	C T	C T	0 0	C T	A G	C C	C C	A A	A A	A A	A A	G G	C C
94	T T	T T	0 0	C T	A G	C C	C C	A A	A G	A G	A C	A G	C T
95	C T	C T	0 0	C T	A G	C C	C C	A A	A A	A A	A A	G G	C C
96	C T	T T	0 0	C C	G G	C C	C C	A A	A G	A A	A A	A G	C C

97	C T	T T	0 0	C C	G G	C C	C C	A A	A G	A G	A C	A G	C T
98	C T	T T	0 0	C C	G G	C C	C C	A A	A A	A A	A A	G G	C C
99	T T	T T	0 0	C C	G G	C C	C C	A A	A A	A A	A A	G G	C C
100	C T	0 0	0 0	C C	G G	0 0	0 0	0 0	A A	0 0	0 0	G G	C C
101	T T	T T	0 0	C T	A G	C C	C C	A A	A G	A G	A C	A G	C T
102	T T	T T	0 0	C C	G G	C C	C C	A A	A A	A A	A A	G G	C C
103	C T	C T	0 0	C T	A G	C C	0 0	A G	A G	A A	A A	G G	C C
104	T T	T T	0 0	C C	G G	C C	C C	A A	A G	A G	A C	A G	C C
105	C C	C T	0 0	C T	A G	C C	C T	A G	A A	A A	A A	G G	C C
106	T T	T T	0 0	C C	G G	C C	C T	A A	G G	A G	A C	A G	C C
107	C T	C T	0 0	C T	A G	C C	C C	A A	A G	A G	A C	A G	C C
108	C C	C C	0 0	T T	A A	C T	C T	A A	A A	A A	A A	G G	C C
109	C C	C T	0 0	C T	A G	C C	C C	A A	A A	A A	A A	G G	C C
110	C T	C T	0 0	C T	A G	C T	C T	A G	G G	A G	A C	A A	C T
111	C T	C T	0 0	C T	A G	C C	C C	A A	A A	A A	A A	G G	C C
112	C T	C T	0 0	C T	A G	C C	C C	A A	A G	A A	A A	A G	C C
113	T T	T T	0 0	C C	G G	C C	C C	A A	A G	A G	A C	A G	C C
114	C C	C C	0 0	T T	A A	C C	C T	A G	A G	A G	A C	A G	C C
115	T T	T T	0 0	C C	G G	C C	C T	A G	G G	A G	A C	A A	C C
116	C T	T T	0 0	C C	G G	C C	C C	A A	A A	A A	A A	G G	C C
117	C C	C T	0 0	C T	A G	C C	C C	A A	A G	A G	A C	A G	C C
118	T T	T T	0 0	C C	G G	C C	C C	A A	A G	A G	A C	A G	C C
119	C T	C T	0 0	C T	A G	C C	C C	A A	A G	A G	A C	A G	C T
120	C T	C T	0 0	C T	A G	C C	C C	A A	A A	A A	A A	G G	C C
121	C C	C C	0 0	T T	A A	C C	C C	A A	A G	A A	A A	A G	C C
122	C C	C C	0 0	T T	A A	C T	C T	A G	A A	A A	A A	G G	C C
123	C T	C T	0 0	C T	A G	C T	C T	A G	A A	A A	A A	G G	C C
124	C T	C T	0 0	C T	A G	C C	C C	A A	A A	A A	A A	G G	C C
125	C C	T T	0 0	C C	G G	C C	C T	A G	A A	0 0	A A	G G	C C
126	C C	C T	0 0	0 0	0 0	C T	C T	A G	A A	A A	A A	G G	C C
127	C T	C T	0 0	C T	A G	C T	C T	A A	A A	A A	A A	G G	C C
128	C C	C C	0 0	C T	A G	T T	T T	G G	G G	A G	A C	A A	C C
129	C T	C T	0 0	0 0	0 0	C C	C C	A A	0 0	0 0	A A	G G	C C

130	C C	C C	0 0	C T	A G	C C	C C	A A	A G	A G	A C	A G	C T
131	C C	C C	0 0	T T	A A	C C	C C	A A	A A	0 0	A A	G G	C C
132	T T	T T	0 0	C C	G G	C T	C T	A G	A G	A G	A C	A G	C C
133	C T	0 0	0 0	0 0	0 0	C T	0 0	A A	0 0	A A	A A	G G	0 0
134	C C	C C	0 0	T T	A A	C C	C C	A A	A G	A G	A C	A G	C C
135	C T	C T	0 0	C T	A G	C C	C T	A G	A G	0 0	0 0	A G	C T
136	T T	T T	0 0	C C	G G	C C	C T	A A	A A	A A	A A	G G	C C
137	T T	T T	0 0	C C	G G	C C	C T	A G	A A	A A	A A	G G	C C
138	C T	C T	0 0	C T	A G	C C	C T	A G	A G	A A	A A	A G	C C
139	C C	C C	0 0	T T	A A	C C	C C	A A	A G	A G	A C	A G	C C
140	T T	T T	0 0	T T	A A	C T	C T	A G	A G	A G	A C	0 0	C C
141	T T	T T	0 0	C C	G G	C C	C C	A A	A A	A A	A A	G G	C C
142	C C	C T	0 0	C T	A G	C C	C C	A A	G G	G G	C C	A A	T T
143	C T	C T	0 0	C T	A G	C T	C T	A G	A G	A G	A C	A G	C C
144	C C	C C	0 0	C T	A G	C C	C C	A A	A A	A A	A A	G G	C C
145	C T	T T	0 0	C C	G G	C C	C C	A A	A A	A A	A A	G G	C C
146	C T	C T	0 0	C T	A G	C C	C C	A A	A A	A A	A A	G G	C C
147	C T	0 0	0 0	C T	A G	C C	0 0	A A	G G	A G	A C	A A	0 0
148	C T	T T	0 0	C C	G G	C C	C C	A A	A A	A A	A A	G G	C C
149	T T	T T	0 0	C C	G G	C C	C T	A A	A A	A A	A A	G G	C C
150	T T	T T	0 0	C T	A G	C C	C C	A A	A A	0 0	A A	G G	C C
151	T T	T T	0 0	C C	G G	C C	C C	A A	A A	A A	A A	G G	C C
152	C C	C C	0 0	T T	A A	C C	C C	A A	A G	A G	A C	A G	C T
153	C T	T T	0 0	C C	G G	C C	C C	A A	A G	A G	A C	A G	C C
154	C T	C T	0 0	0 0	0 0	C T	C T	A G	0 0	A A	A A	G G	C C
155	C C	C C	0 0	T T	A A	C C	C C	A A	A G	A G	A C	A G	C C
156	C T	C T	0 0	C T	A G	C C	C C	A A	A G	A G	A C	A G	C T
157	C T	T T	0 0	C C	G G	C C	C C	A A	A A	A A	A A	G G	C C
158	C C	C C	0 0	0 0	A A	C C	C C	A A	0 0	0 0	A A	G G	C C
159	C T	C T	0 0	C T	A G	C C	C C	A A	A A	A A	A A	G G	C C
160	T T	T T	0 0	0 0	0 0	0 0	C C	0 0	0 0	0 0	0 0	G G	C C
161	T T	T T	0 0	C C	G G	C C	C T	A A	A A	A A	A A	G G	C C
162	C T	C T	0 0	C T	A G	C T	C T	A G	A A	A A	A A	G G	C C

163	C C	T T	0 0	C C	G G	C C	C T	A G	A A	A A	A A	G G	C C
164	C C	C C	0 0	T T	A A	C C	C T	A A	A A	A A	A A	G G	C C
165	T T	T T	0 0	C C	G G	C T	T T	G G	A G	A G	A C	A G	C C
166	C T	T T	0 0	C C	G G	C C	C C	A A	G G	A A	A A	A A	C C
167	C T	C T	0 0	C T	A G	C C	C C	A A	A A	A A	A A	G G	C C
168	C T	C T	0 0	C T	A G	C T	C T	A G	A A	A A	A A	G G	C C
169	T T	T T	0 0	C C	G G	C C	C C	A A	A A	A A	A A	G G	C C
170	C C	C C	0 0	C C	G G	C C	C C	A A	A A	A A	A A	G G	C C
171	C T	C T	0 0	T T	A A	C T	C T	A G	G G	A G	A C	A A	C T
172	0 0	T T	0 0	0 0	0 0	C C	C C	A A	0 0	A G	A C	0 0	C T
173	T T	T T	0 0	C C	G G	C C	C T	A G	A G	A A	A A	A G	C C
174	C T	C T	0 0	C T	A G	C C	C T	A G	A A	A A	A A	G G	C C
175	C C	C C	0 0	T T	A A	C C	C T	A G	A A	A A	A A	G G	C C
176	T T	T T	0 0	C C	G G	C T	C T	A G	A A	A A	A A	G G	C C
177	C T	C T	0 0	C T	A G	C C	C C	A A	A G	A G	0 0	A G	0 0
178	T T	T T	0 0	C C	G G	C C	C C	A A	A G	A G	0 0	A G	0 0
179	C C	C C	0 0	T T	A A	C C	C T	A G	G G	0 0	0 0	A A	0 0
180	C T	C T	0 0	C T	A G	C T	C C	A G	A A	A A	0 0	G G	0 0
181	C T	C T	0 0	C T	A G	C C	C C	A A	A G	A A	0 0	A G	0 0
182	T T	T T	0 0	C C	G G	C C	C C	A A	A A	A A	0 0	G G	0 0
183	C C	C C	0 0	T T	A A	C C	C C	A A	A G	A G	0 0	A G	0 0
184	C T	C T	0 0	C C	G G	C C	T T	G G	G G	G G	0 0	A A	0 0
185	T T	0 0	0 0	C C	G G	C T	0 0	A G	A G	A G	0 0	A G	0 0
186	T T	T T	0 0	C T	A G	C C	C C	A A	A A	A A	0 0	G G	C C
187	C T	T T	0 0	C C	G G	C C	C C	A A	A A	0 0	0 0	G G	0 0
188	C T	C T	0 0	C T	A G	C C	C C	A A	A G	A G	0 0	A G	C T
189	T T	T T	0 0	C C	G G	C C	C C	A G	A G	A G	0 0	A G	0 0
190	T T	T T	0 0	C C	G G	C T	C C	A A	A A	A A	0 0	G G	C C
191	C C	C C	0 0	T T	A A	C C	C C	A A	A G	A A	0 0	A G	0 0
192	T T	T T	0 0	C C	G G	C C	C C	A A	A A	A A	0 0	G G	0 0
193	C T	C T	0 0	C T	A G	C C	C C	A A	A A	A A	0 0	G G	0 0
194	C T	T T	0 0	C C	G G	C T	C T	A A	A A	A A	0 0	G G	C C
195	T T	T T	0 0	C C	G G	C C	C C	A A	A G	A G	0 0	A G	C T

196	C T	C T	0 0	T T	A A	C C	C C	A A	A G	A G	0 0	A G	C T
197	C C	C T	0 0	C C	G G	C C	C C	A A	A A	A A	0 0	G G	C C
198	C C	C C	0 0	0 0	A A	C C	C C	A A	A G	A A	0 0	A G	C C
199	C C	C T	0 0	C T	A G	C T	C T	A G	A A	A A	0 0	G G	C C
200	T T	T T	0 0	C C	G G	C C	C C	A A	A G	A G	0 0	A G	C T
201	T T	T T	0 0	C C	G G	C C	C C	A A	A G	A G	0 0	A G	C T
202	C C	C C	0 0	T T	A A	C C	C T	A G	A G	A G	0 0	A G	C C
203	T T	T T	0 0	C C	G G	C C	C C	A A	A A	A A	0 0	G G	0 0
204	C T	C T	0 0	C T	A G	C C	C C	A A	A G	A G	0 0	A G	C T
205	C T	T T	0 0	C C	G G	C C	C C	A A	A G	A G	0 0	A G	C T
206	T T	T T	0 0	C C	G G	C T	C T	A G	A G	A G	0 0	A G	C C
207	C T	C T	0 0	C T	A G	C C	C C	A A	A A	A A	0 0	G G	C C
208	T T	T T	0 0	C C	G G	C T	C T	A A	A A	A A	0 0	G G	C C
209	T T	T T	0 0	C C	G G	C T	C T	A A	A A	A A	0 0	G G	0 0
210	T T	T T	0 0	C C	G G	C T	C T	A A	A A	A A	0 0	G G	C C
211	T T	T T	0 0	C T	A G	C T	C T	A G	G G	G G	0 0	A A	C T
212	T T	T T	0 0	C T	A G	C T	T T	A G	A G	A G	0 0	A G	C C
213	C T	C T	0 0	C T	A G	C T	C T	A A	A A	A A	0 0	G G	C C
214	T T	T T	0 0	C C	G G	C T	C T	A G	A G	A G	0 0	A G	C C
215	T T	T T	0 0	C C	G G	C C	C C	A A	A A	A A	0 0	G G	C C
216	C T	C T	0 0	C T	A G	C C	C C	A A	A G	A G	0 0	A G	C C
217	T T	T T	0 0	T T	A A	C C	C T	A A	A G	A G	0 0	A G	C T
218	T T	T T	0 0	C T	A G	C T	C T	A G	A G	A G	0 0	A G	C T
219	C C	C T	0 0	C T	A G	C C	C C	A A	A A	A A	0 0	G G	C C
220	T T	T T	0 0	C C	G G	C C	C C	A A	A A	A A	0 0	G G	C C
221	T T	T T	0 0	C C	G G	C C	C T	A G	A A	A A	0 0	G G	C C
222	C T	C T	0 0	C T	A G	C T	C T	A A	A A	A A	0 0	G G	C C
223	C T	C T	0 0	T T	A A	C C	C T	A G	A G	A G	0 0	A G	C C
224	0 0	C T	0 0	0 0	A G	C C	C C	A A	A A	A A	0 0	G G	C C
225	T T	T T	0 0	C C	G G	C C	C C	A A	A G	A G	0 0	A G	C T
226	T T	T T	0 0	C C	G G	T T	T T	A G	A G	A G	0 0	A G	C C
227	C T	C T	0 0	C T	A G	C C	C C	A A	A A	A A	0 0	G G	C C
228	T T	T T	0 0	C C	G G	C T	C T	A G	G G	G G	0 0	A A	C C

229	C T	C T	0 0	C T	G G	C C	C C	A A	A G	A G	0 0	A G	C T
230	T T	T T	0 0	C C	G G	C C	C C	A A	A G	A G	0 0	0 0	C T
231	C C	C T	0 0	C C	G G	C T	C T	A A	A G	A G	0 0	A G	C C
232	C C	C T	0 0	C T	A G	C C	0 0	A A	A G	A G	0 0	A G	C T
233	C C	C C	0 0	T T	A A	C C	C C	A A	A A	A A	0 0	G G	C C
234	T T	T T	0 0	C T	G G	C C	C T	A G	A A	A A	0 0	G G	C C
235	C T	T T	0 0	C C	G G	C C	C C	A A	A A	A A	0 0	G G	C C
236	T T	T T	0 0	C C	G G	C C	C C	A A	A A	A A	0 0	G G	C C
237	T T	T T	0 0	C T	A G	C C	C C	A A	A A	A A	0 0	G G	C C
238	C T	C T	0 0	C C	G G	C T	C T	A G	A G	A G	0 0	A G	C T
239	T T	T T	0 0	C C	G G	C T	T T	G G	A A	A A	0 0	G G	C C
240	C T	C T	0 0	C T	A G	C T	C T	A G	A A	A A	0 0	G G	C C
241	C T	T T	0 0	C C	G G	C C	C C	A A	A A	A A	0 0	G G	C C
242	T T	T T	0 0	C C	G G	C C	C C	A A	A G	A G	0 0	A G	C C
243	T T	T T	0 0	C C	G G	C C	C C	A A	A A	A A	0 0	G G	C C
244	C T	C T	0 0	C T	A G	C C	C C	A A	A A	A A	0 0	G G	C C
245	C T	C T	0 0	C T	A G	C C	C C	A A	A A	A A	0 0	G G	C C
246	C C	C T	0 0	C T	A G	C C	C C	A A	A A	A A	0 0	G G	C C
247	T T	T T	0 0	C T	A G	C C	C T	A G	A G	A A	0 0	A G	C C
248	C T	C T	0 0	T T	A A	C C	C C	A A	A G	A G	0 0	A G	C C
249	T T	T T	0 0	C C	G G	C C	C C	A A	A G	A G	0 0	A G	C T
250	C T	C T	0 0	C T	A G	C C	C C	A A	A A	A A	0 0	G G	C C
251	C C	C C	0 0	T T	A A	C C	C C	A A	A A	A A	0 0	G G	C C
252	C C	C T	0 0	C T	A G	C C	C C	A A	A G	A G	0 0	A G	C T
253	C C	C C	0 0	C T	A G	C C	C C	A A	A A	A A	0 0	G G	C C
254	C C	C T	0 0	C T	A G	C C	C C	A A	A G	A G	0 0	A G	C C
255	C C	C C	0 0	T T	A A	C C	C C	A A	A G	A G	0 0	A G	C T
256	C T	T T	0 0	C C	G G	C C	C C	A A	A A	A A	0 0	G G	C C
257	T T	T T	0 0	C T	A G	C T	C T	A G	A G	A A	0 0	A G	C C
258	C C	C C	0 0	T T	A A	C C	0 0	A A	A G	A G	0 0	A G	C C
259	T T	T T	0 0	C C	G G	T T	T T	G G	G G	G G	0 0	A A	C C
260	C T	C T	0 0	C T	A G	C T	C T	A G	A G	A G	0 0	A G	C C
261	0 0	C T	0 0	0 0	0 0	C T	C T	A G	0 0	A G	0 0	0 0	C C

262	C C	C C	0 0	T T	A A	C C	C C	A A	A A	A A	0 0	G G	C C
263	C T	C T	0 0	C T	A G	C C	C T	A G	A G	A G	0 0	A G	C T
264	C T	C T	0 0	C T	A G	C C	C T	A A	A A	A A	0 0	G G	C C
265	C T	C T	0 0	T T	A A	C C	C C	A A	A G	A G	A C	A G	C T
266	T T	T T	0 0	C T	A G	C C	C C	A A	A A	A A	A A	G G	C C
267	C T	C T	0 0	C T	A G	C C	C C	A A	A A	A A	A A	G G	C C
268	T T	T T	0 0	C C	G G	C T	C T	A G	A G	A G	A C	A G	C C
269	C C	C C	0 0	C T	A G	C T	T T	A G	A A	A A	A A	G G	C C
270	T T	T T	0 0	C T	A G	C C	C C	A A	A G	A A	A A	A G	C C
271	T T	T T	0 0	C T	A G	C T	C T	A G	A G	A G	A C	A G	C C
272	C T	C T	0 0	C T	A G	C C	C C	A A	A A	A A	A A	G G	C C
273	T T	T T	0 0	C C	G G	C C	C T	A G	A A	A A	A A	G G	C C
274	C C	C C	0 0	T T	A A	C T	C T	A G	A G	A A	A A	A G	C C
275	C C	C C	0 0	T T	A A	C C	C T	A G	A G	A G	A C	A G	C T
276	0 0	C C	0 0	0 0	0 0	C C	C C	A A	0 0	A A	A A	0 0	C C
277	C C	T T	0 0	C C	G G	C C	C C	A A	A A	A A	A A	G G	C C
278	T T	T T	0 0	C T	A G	C C	C C	A A	A G	A A	A A	A G	C C
279	C C	C C	0 0	T T	A A	C C	C C	A A	G G	A A	A A	A A	C C
280	C T	C T	0 0	C T	A G	C C	C T	A G	A G	A G	A C	A G	C C
281	T T	T T	0 0	C C	G G	C T	C T	A G	A A	A A	A A	G G	C C
282	T T	T T	0 0	C C	G G	C C	C C	A A	A A	A A	A A	G G	C C
283	C C	C T	0 0	C T	A G	C C	C C	A A	A A	A A	A A	G G	C C
284	T T	T T	0 0	C C	G G	C C	C T	A G	A G	A G	A C	A G	C C

GENE	GRIK2	GRIK2	GRIK2	GRIK2	GRIK2	GRIK2	GRIK2	GRIK2	SGK1	SGK1	SGK1	HIP1	HIP1
SNP/Sample	rs2518344	rs6930752	rs3213607	rs2852612	rs2852619	rs2782901	rs1232241	rs3849205	rs9402571	rs1057293	rs1743966	rs1167801	rs6962352
1	0 0	A A	C C	0 0	G G	T T	T T	C C	T T	C C	T T	T T	0 0
2	0 0	A C	C C	A C	A A	C T	C T	C T	T T	C T	C T	T T	0 0
3	0 0	C C	C C	A C	A A	T T	C T	C C	T T	C C	T T	T T	0 0
4	0 0	A C	A C	A A	A A	C T	C C	C T	G T	C C	C T	T T	0 0
5	0 0	C C	C C	A C	A G	C T	C T	C T	T T	C C	T T	C T	0 0
6	0 0	A C	A C	A A	A A	C T	C T	C C	T T	C C	T T	T T	0 0
7	0 0	A A	C C	0 0	A G	T T	T T	C T	T T	C C	T T	T T	0 0
8	0 0	C C	C C	0 0	A A	C T	C T	C T	G T	C C	T T	T T	0 0
9	0 0	C C	C C	A A	A A	C C	C C	C C	G T	C C	T T	T T	0 0
10	0 0	C C	C C	A C	A G	T T	C C	C T	G G	C C	T T	T T	0 0
11	0 0	A C	C C	0 0	G G	T T	C T	C T	G T	C C	T T	C C	0 0
12	0 0	C C	A C	0 0	A A	C T	T T	C C	G T	0 0	T T	C T	0 0
13	0 0	A C	C C	A A	A A	C T	C C	C T	G T	C C	C T	T T	0 0
14	0 0	A C	C C	A C	A G	C T	C T	C T	T T	C C	T T	T T	0 0
15	0 0	C C	C C	A A	A A	C C	C C	C C	T T	C C	C T	T T	0 0
16	0 0	A C	C C	A C	A A	T T	T T	C T	G T	C C	0 0	T T	0 0
17	0 0	A C	C C	A C	A G	C T	C T	C T	T T	C C	T T	C C	0 0
18	0 0	A C	C C	A C	A A	C T	C T	T T	T T	C C	C T	T T	0 0
19	0 0	A C	C C	A C	A G	C T	C T	C C	T T	C C	T T	C T	0 0
20	0 0	A C	C C	A A	A A	T T	C T	C T	T T	C T	C T	T T	0 0
21	0 0	A C	C C	A C	A G	C T	C T	C C	T T	C C	T T	T T	0 0
22	0 0	A C	C C	A C	A A	T T	T T	C C	G T	C C	T T	T T	0 0
23	0 0	C C	C C	A A	A A	C T	C T	C T	G T	C C	T T	C T	0 0
24	0 0	A C	C C	A C	A G	T T	C T	C T	G T	C C	T T	T T	0 0
25	0 0	C C	C C	C C	A G	T T	T T	C T	T T	C T	C T	T T	0 0
26	0 0	A C	C C	A C	A G	C T	C T	C C	T T	C C	T T	T T	0 0
27	0 0	C C	C C	A C	A G	C T	T T	C C	T T	C T	C T	T T	0 0
28	0 0	C C	C C	A C	A G	C T	C T	C T	G T	C C	C T	T T	0 0
29	0 0	C C	C C	A C	A G	C T	C T	C T	T T	C T	C T	T T	0 0
30	0 0	A C	C C	A C	A G	C T	C T	C C	T T	C T	C T	T T	0 0

31	0 0	A C	A C	A A	A A	C T	T T	C C	G T	C C	T T	T T	0 0
32	0 0	C C	C C	A C	A G	C T	C T	C C	T T	C C	T T	C T	0 0
33	0 0	A A	C C	A C	A G	C T	C T	C T	T T	C C	T T	T T	0 0
34	0 0	A C	C C	A C	A G	C T	C T	C C	T T	C T	C T	T T	0 0
35	0 0	A C	A C	A C	A A	T T	C T	T T	T T	C C	C T	C T	0 0
36	0 0	C C	C C	A A	A A	C T	C C	C T	T T	C T	C T	C T	0 0
37	0 0	C C	C C	0 0	0 0	T T	T T	0 0	G T	0 0	T T	T T	0 0
38	0 0	A C	C C	A A	A A	C C	C C	C C	T T	C C	T T	T T	0 0
39	0 0	A C	C C	0 0	0 0	C C	0 0	0 0	0 0	0 0	0 0	T T	0 0
40	0 0	A A	C C	A C	A G	T T	C T	C T	T T	C T	C C	T T	G G
41	0 0	C C	C C	0 0	0 0	C T	0 0	0 0	0 0	0 0	0 0	T T	0 0
42	0 0	A A	C C	A C	A G	C T	C T	C T	T T	C C	C T	T T	0 0
43	0 0	A C	C C	0 0	0 0	C T	0 0	0 0	0 0	0 0	0 0	C T	0 0
44	0 0	A A	C C	A C	A G	C T	C T	C C	T T	C T	C T	T T	0 0
45	0 0	A A	C C	0 0	A G	T T	T T	0 0	G T	0 0	T T	T T	0 0
46	0 0	C C	C C	C C	A G	T T	T T	C T	T T	C C	T T	C T	0 0
47	0 0	A C	C C	0 0	A G	C T	C T	0 0	T T	0 0	C T	T T	0 0
48	0 0	C C	C C	C C	G G	T T	T T	C T	T T	C C	T T	T T	0 0
49	0 0	C C	C C	A A	A A	C T	C T	C C	T T	C T	C T	T T	0 0
50	0 0	C C	C C	0 0	0 0	C T	0 0	0 0	0 0	0 0	0 0	T T	0 0
51	0 0	C C	C C	A C	A G	C C	C C	C T	T T	C T	C C	T T	0 0
52	0 0	A C	C C	0 0	0 0	C T	0 0	0 0	0 0	0 0	0 0	T T	0 0
53	0 0	C C	C C	A C	A G	C T	C T	C C	T T	C C	T T	C T	0 0
54	0 0	C C	C C	A A	A A	C C	C C	0 0	G T	0 0	0 0	T T	0 0
55	0 0	C C	A C	A A	A A	C T	C T	C C	T T	C C	T T	T T	0 0
56	0 0	A C	C C	0 0	A G	C T	C T	0 0	T T	0 0	0 0	T T	0 0
57	0 0	C C	C C	A A	A A	C T	C C	T T	T T	C C	T T	T T	0 0
58	0 0	C C	C C	A A	A A	C C	C C	C T	T T	C T	C T	T T	0 0
59	0 0	A A	C C	A A	A A	C C	C C	C T	T T	C T	C T	T T	0 0
60	0 0	C C	C C	C C	A G	T T	T T	C T	T T	C C	C T	T T	0 0
61	0 0	A C	C C	A C	A G	C T	T T	C C	T T	C C	C T	T T	0 0
62	0 0	A C	C C	0 0	A G	C T	T T	0 0	G T	0 0	T T	T T	0 0
63	0 0	A C	A C	A A	A A	C T	C C	C T	T T	C C	C T	C T	0 0

64	0 0	A A	A C	C C	G G	T T	T T	C C	T T	C C	T T	T T	0 0
65	0 0	C C	C C	A A	A A	C C	C C	C T	G T	C C	T T	C T	0 0
66	0 0	A A	C C	0 0	0 0	T T	C T	0 0	T T	0 0	C T	T T	0 0
67	0 0	C C	C C	A C	G G	T T	T T	C C	T T	C C	T T	T T	0 0
68	0 0	A A	C C	A A	A A	C T	C C	C T	T T	C C	T T	C T	0 0
69	0 0	C C	C C	A C	A G	C T	C T	T T	G T	C C	T T	T T	0 0
70	0 0	C C	C C	A A	A A	C C	C T	C T	T T	C C	T T	T T	0 0
71	0 0	A C	C C	C C	A G	T T	T T	C T	T T	C T	C T	T T	0 0
72	0 0	A A	C C	A A	A A	C C	C T	C T	T T	C C	T T	T T	0 0
73	0 0	A C	C C	C C	G G	T T	T T	C C	T T	C C	C T	C C	0 0
74	0 0	C C	C C	A C	A G	C T	T T	C C	T T	C C	T T	C C	0 0
75	0 0	A A	C C	A C	A G	C T	C T	C T	G T	C C	T T	T T	0 0
76	0 0	C C	A C	A A	A A	C T	C T	C C	G T	C C	T T	T T	0 0
77	0 0	C C	C C	C C	A G	T T	T T	C T	G T	C C	T T	C T	0 0
78	0 0	A C	C C	A C	A G	C T	C T	C T	T T	C C	C T	T T	0 0
79	0 0	A C	C C	C C	A G	T T	T T	C T	G T	C C	T T	T T	0 0
80	0 0	A C	C C	A C	G G	T T	T T	C C	G T	C C	T T	C T	0 0
81	0 0	A C	C C	A C	A G	C T	C T	C T	G T	C C	T T	T T	0 0
82	0 0	A C	C C	C C	G G	T T	T T	C C	G T	C C	T T	T T	0 0
83	0 0	C C	A C	A C	A G	C T	C T	C C	G G	C C	T T	T T	0 0
84	0 0	C C	C C	A C	A G	T T	C T	C T	T T	C C	C T	T T	0 0
85	0 0	A A	A C	A A	A A	C T	C T	C T	G T	C C	C T	C T	0 0
86	0 0	C C	C C	C C	A G	T T	T T	C C	T T	C C	T T	T T	0 0
87	0 0	A C	C C	A A	A A	C T	C C	C T	G T	C C	T T	T T	0 0
88	0 0	AC	CC	AC	AG	TT	CT	CT	TT	CC	TT	TT	0 0
89	0 0	0 0	0 0	A A	A A	C T	0 0	C C	G T	C C	T T	C T	A G
90	0 0	A A	C C	A A	A A	T T	C C	C T	T T	C C	T T	T T	A A
91	0 0	C C	C C	A A	A A	C C	C T	T T	G T	C C	T T	C T	A G
92	0 0	C C	C C	A C	A A	T T	C T	C T	T T	C C	T T	T T	A A
93	0 0	C C	C C	A C	A A	C T	C T	C T	G T	C C	T T	T T	G G
94	0 0	A C	C C	A C	A G	C T	C T	C T	G G	C C	T T	T T	A A
95	0 0	A C	A C	A C	A G	T T	C T	C T	T T	C T	C T	C T	G G
96	0 0	A C	C C	A C	A G	C T	C T	C C	G T	C C	T T	C T	A A

97	0 0	C C	C C	A C	A A	C T	C T	T T	G T	C C	T T	C T	A G
98	0 0	A C	C C	A A	A A	T T	C T	C C	T T	C C	C T	T T	A A
99	0 0	C C	C C	C C	G G	T T	T T	C C	T T	C C	T T	T T	A A
100	0 0	A C	C C	0 0	0 0	T T	0 0	0 0	0 0	0 0	0 0	0 0	0 0
101	0 0	A C	C C	C C	G G	T T	T T	C C	T T	C T	C T	T T	A G
102	0 0	A C	C C	C C	G G	T T	T T	C C	T T	C T	C C	T T	A G
103	0 0	A C	0 0	A C	A A	T T	C T	T T	G T	C C	T T	T T	A A
104	0 0	C C	C C	A C	G G	T T	T T	C C	T T	C C	T T	T T	A G
105	0 0	C C	C C	A C	A G	C T	C T	C T	G T	C T	C T	T T	A A
106	0 0	A C	C C	A C	A G	C T	C T	T T	T T	C T	C T	T T	A A
107	0 0	A C	C C	C C	A G	T T	T T	C C	G G	C C	C T	C T	A G
108	0 0	C C	C C	A C	A A	C T	C T	C T	T T	C C	T T	T T	A G
109	0 0	A C	C C	A A	A A	C T	C C	C C	G T	C C	T T	T T	A G
110	0 0	C C	C C	C C	G G	T T	T T	C C	G T	C T	C T	T T	A A
111	0 0	A C	C C	C C	A A	C T	C T	T T	G T	C C	C T	T T	G G
112	0 0	C C	C C	A A	A A	C C	C C	C T	T T	C C	C T	C T	A G
113	0 0	A C	C C	A A	A G	T T	T T	C C	G T	C C	T T	T T	A A
114	0 0	C C	C C	A C	A G	C T	C T	C T	G T	C C	T T	T T	A G
115	0 0	A C	C C	A C	A G	C T	C T	C T	G T	C C	T T	T T	A G
116	0 0	A A	C C	A A	A A	C C	C C	C T	G T	C C	T T	T T	G G
117	0 0	A A	C C	A A	A A	C T	C C	C C	T T	C C	T T	C T	A G
118	0 0	A C	C C	A A	A A	C C	C C	C T	T T	C C	C T	T T	A G
119	0 0	A C	C C	A C	A A	C T	C T	C T	T T	C C	T T	T T	A G
120	0 0	A C	C C	A C	A G	C T	C T	C T	T T	C T	C T	T T	A G
121	0 0	A C	C C	A C	A G	T T	C T	C T	T T	C C	T T	T T	A G
122	0 0	A A	C C	A C	A A	T T	C T	C T	T T	C T	C C	T T	A A
123	0 0	A C	C C	C C	A G	T T	T T	C C	G T	C C	T T	T T	G G
124	0 0	A C	C C	A C	A A	C T	C T	T T	G T	C C	C T	T T	A G
125	0 0	A C	C C	0 0	G G	T T	T T	0 0	T T	0 0	T T	T T	A A
126	0 0	A C	C C	A A	A A	C C	C C	T T	G T	C C	T T	T T	A G
127	0 0	A C	C C	A A	A A	C C	C C	C T	T T	C T	C T	C C	G G
128	0 0	C C	C C	A C	A G	C T	C T	C T	T T	C C	T T	T T	A A
129	0 0	C C	A C	0 0	A G	T T	C T	0 0	G T	0 0	T T	T T	0 0

130	0 0	C C	C C	A C	A G	C T	C T	C T	G G	C C	T T	C T	A G
131	0 0	A C	C C	C C	A G	T T	T T	C C	T T	0 0	T T	C T	G G
132	0 0	A C	C C	C C	G G	T T	T T	C C	G T	C C	T T	T T	A G
133	0 0	0 0	0 0	A A	A G	0 0	C T	C T	G G	C C	T T	0 0	A G
134	0 0	A C	C C	A A	A A	C C	C C	T T	T T	C C	T T	T T	A A
135	0 0	A C	C C	A A	A A	C C	C T	0 0	T T	0 0	C T	C T	0 0
136	0 0	C C	C C	A C	A G	C T	C T	C T	T T	C C	T T	C T	G G
137	0 0	A A	C C	C C	A A	T T	T T	T T	T T	C C	T T	T T	A G
138	0 0	C C	C C	A C	A G	C T	C T	C T	T T	C T	C T	T T	G G
139	0 0	C C	C C	A A	A A	T T	C T	C C	G T	C C	C T	T T	A A
140	0 0	A C	C C	A C	G G	T T	T T	C T	T T	C C	T T	T T	A G
141	0 0	A C	C C	C C	A A	T T	T T	C T	T T	C T	C T	T T	A A
142	0 0	C C	C C	A C	G G	T T	T T	C C	G T	C C	T T	T T	A A
143	0 0	C C	C C	A C	A G	C T	C T	C T	T T	C C	T T	T T	A A
144	0 0	C C	C C	A A	A A	C T	C C	T T	T T	C T	C T	T T	A G
145	0 0	A C	A C	A C	A G	T T	C T	C T	G T	C T	C T	T T	A A
146	0 0	C C	A C	A A	A A	C T	C C	C T	G T	C T	C T	T T	A A
147	0 0	0 0	0 0	A A	A A	0 0	C T	T T	T T	C C	C T	0 0	A G
148	0 0	A C	C C	C C	G G	T T	T T	C C	T T	C C	C T	T T	A A
149	0 0	A C	A C	A C	A G	T T	T T	C C	T T	C C	T T	T T	A A
150	0 0	A C	C C	A C	A A	C T	0 0	T T	T T	0 0	0 0	T T	A A
151	0 0	A C	C C	A C	A G	C T	C T	C C	G T	C T	C T	T T	A G
152	0 0	C C	A C	A A	A A	C T	C C	T T	T T	C T	C T	C C	G G
153	0 0	A C	C C	A C	A G	C T	C T	C C	T T	C T	C T	T T	G G
154	0 0	A C	C C	A C	A G	C T	C T	C C	T T	C C	T T	C T	A G
155	0 0	A C	C C	A C	A G	C T	C T	C T	G G	C C	T T	T T	A A
156	0 0	C C	C C	A A	A A	C T	C T	C T	T T	C T	C C	T T	A G
157	0 0	A C	C C	A C	A A	T T	T T	C T	T T	C C	T T	C T	G G
158	0 0	0 0	C C	A C	A G	C T	0 0	0 0	T T	0 0	T T	T T	A G
159	0 0	A C	A C	A A	A A	C C	C C	T T	G T	C C	C T	C T	A G
160	0 0	A C	C C	0 0	0 0	C T	0 0	0 0	0 0	0 0	0 0	C T	0 0
161	0 0	A C	C C	C C	A G	T T	T T	C C	T T	C C	T T	T T	A A
162	0 0	C C	C C	A C	A G	T T	C T	C T	T T	C C	T T	C T	A G

163	0 0	A C	C C	A C	A A	C T	C T	C T	T T	C T	C T	T T	A A
164	0 0	A C	C C	A A	A A	T T	C T	C T	T T	C C	T T	C T	A G
165	0 0	A C	C C	C C	A G	T T	C T	C T	T T	C C	T T	C T	G G
166	0 0	A C	A C	A A	A A	T T	C C	C T	G T	C C	T T	T T	A A
167	0 0	A A	C C	A A	A A	C C	C C	C T	G T	C C	T T	T T	A G
168	0 0	C C	C C	A A	A A	C C	C C	C T	T T	C C	T T	T T	A G
169	0 0	A C	C C	A A	A A	T T	C T	C C	G T	C C	T T	C T	A G
170	0 0	C C	C C	A C	A G	C T	C T	C T	T T	C T	C C	C T	G G
171	0 0	C C	C C	A C	A A	C T	C T	C T	G G	C C	T T	C T	A G
172	0 0	C C	C C	A C	A G	T T	C C	T T	G T	C C	T T	C T	A G
173	0 0	C C	C C	A A	A A	C T	C C	T T	T T	C T	C T	T T	A G
174	0 0	C C	A C	A C	A G	C T	T T	C C	T T	C C	T T	T T	A A
175	0 0	C C	C C	C C	A A	T T	T T	T T	T T	C C	T T	T T	A A
176	0 0	A C	C C	A C	A A	C T	C T	T T	T T	C T	C C	C T	G G
177	0 0	A C	C C	A C	A A	C T	C T	C C	T T	0 0	C T	T T	0 0
178	0 0	C C	A C	A C	A A	C T	T T	C C	G T	0 0	T T	C T	0 0
179	0 0	A A	C C	A C	G G	T T	T T	C C	G T	0 0	T T	T T	0 0
180	0 0	C C	C C	A C	A A	T T	C T	C T	G T	0 0	T T	T T	0 0
181	0 0	A A	C C	A C	A G	C T	C T	C C	T T	0 0	C T	C T	0 0
182	0 0	C C	C C	A A	A A	C C	C C	C T	T T	0 0	T T	T T	0 0
183	0 0	A C	C C	A A	A A	C C	C C	C T	T T	0 0	T T	C T	0 0
184	0 0	A A	C C	A C	A G	C T	0 0	C T	T T	0 0	T T	T T	0 0
185	0 0	0 0	0 0	A C	A G	0 0	0 0	C C	G T	0 0	T T	0 0	0 0
186	0 0	C C	C C	A C	A A	C T	C T	T T	T T	0 0	T T	T T	0 0
187	0 0	A C	C C	A A	A A	C T	C C	T T	G T	0 0	T T	T T	0 0
188	0 0	A A	C C	A C	A G	T T	C T	C T	G T	0 0	T T	0 0	0 0
189	0 0	C C	C C	A A	A G	C T	C T	C C	T T	0 0	T T	T T	0 0
190	0 0	A A	C C	A A	A G	C T	C T	C T	T T	0 0	T T	T T	0 0
191	0 0	A C	A C	A A	A A	C T	C C	T T	G G	0 0	T T	T T	0 0
192	0 0	A C	A C	A C	A G	C T	C T	C C	T T	0 0	T T	T T	0 0
193	0 0	C C	C C	A C	A A	C T	C T	C C	T T	0 0	C T	T T	0 0
194	0 0	A C	C C	A A	A A	C C	C C	T T	T T	0 0	C T	T T	0 0
195	0 0	C C	C C	A A	A A	C T	C C	T T	T T	0 0	T T	T T	0 0

196	0 0	A C	A C	A A	A A	C T	C T	C C	G T	0 0	T T	T T	0 0
197	0 0	A C	C C	A C	A G	C T	C T	C T	G G	0 0	T T	C T	0 0
198	0 0	A C	C C	C C	A G	T T	C T	C T	T T	0 0	C T	T T	0 0
199	0 0	A C	C C	A A	A A	C T	C C	T T	G T	0 0	T T	T T	0 0
200	0 0	A C	C C	A C	A G	C T	C T	C C	G T	0 0	T T	T T	0 0
201	0 0	C C	C C	A C	A A	C T	C T	C T	T T	0 0	C T	C T	0 0
202	0 0	A C	C C	A C	A G	T T	C T	C T	T T	0 0	T T	T T	0 0
203	0 0	A C	C C	C C	G G	T T	C T	C T	T T	0 0	T T	T T	0 0
204	0 0	A A	C C	A C	A G	C T	C T	C T	T T	0 0	C T	T T	0 0
205	0 0	C C	C C	A C	A G	C T	C T	C T	T T	0 0	T T	C C	0 0
206	0 0	C C	C C	A C	A G	T T	C T	C T	T T	0 0	C T	C T	0 0
207	0 0	A A	C C	C C	A G	T T	T T	C T	G T	0 0	C T	T T	0 0
208	0 0	A C	C C	A C	A G	C T	T T	C C	T T	0 0	T T	T T	0 0
209	0 0	A C	C C	A C	A G	C T	T T	C C	T T	0 0	T T	T T	0 0
210	0 0	C C	C C	A C	A A	C T	C T	C T	G T	0 0	T T	T T	0 0
211	0 0	A A	C C	A C	A G	C T	C T	C C	G T	0 0	T T	T T	0 0
212	0 0	C C	C C	A C	A G	T T	C T	C C	G T	0 0	T T	T T	0 0
213	0 0	A C	C C	A C	A G	C T	C T	T T	T T	0 0	C T	T T	0 0
214	0 0	C C	C C	C C	A G	T T	T T	C C	T T	0 0	C T	T T	0 0
215	0 0	C C	C C	A C	A G	C T	C T	C T	T T	0 0	C T	T T	0 0
216	0 0	A A	C C	A C	A A	C T	C T	T T	T T	0 0	C T	T T	0 0
217	0 0	C C	C C	A A	A G	C T	C T	C T	T T	0 0	C T	T T	0 0
218	0 0	C C	C C	A A	A A	C C	C C	C T	T T	0 0	T T	T T	0 0
219	0 0	A C	A C	A C	A G	C T	C T	C C	T T	0 0	T T	T T	0 0
220	0 0	A A	C C	C C	A G	T T	T T	C T	T T	0 0	C T	C T	0 0
221	0 0	C C	C C	A A	A A	C C	C C	T T	T T	0 0	C T	C T	0 0
222	0 0	C C	C C	A C	A G	C T	C T	C T	G T	0 0	T T	T T	0 0
223	0 0	A C	C C	A C	A G	C T	C T	C C	T T	0 0	C T	T T	0 0
224	0 0	C C	C C	C C	G G	T T	C T	T T	T T	0 0	T T	T T	0 0
225	0 0	C C	C C	A C	A G	C T	C T	T T	T T	0 0	C T	T T	0 0
226	0 0	C C	C C	A A	A A	C C	C C	C C	G T	0 0	C T	T T	0 0
227	0 0	C C	C C	A C	A G	T T	C T	C T	G T	0 0	T T	T T	0 0
228	0 0	A A	A A	A A	A A	T T	T T	C C	T T	0 0	C T	C T	0 0

229	0 0	A A	C C	A A	A G	T T	T T	C C	T T	0 0	C T	C T	0 0
230	0 0	A C	C C	A A	A A	C T	C T	C T	T T	0 0	T T	C T	0 0
231	0 0	C C	C C	A C	A A	T T	C T	C T	T T	0 0	T T	T T	0 0
232	0 0	A C	C C	A A	A A	C C	C C	C C	G T	0 0	T T	T T	0 0
233	0 0	C C	C C	A C	A G	T T	C T	C T	T T	0 0	T T	T T	0 0
234	0 0	C C	C C	A C	A G	C T	C T	C T	T T	0 0	T T	C T	0 0
235	0 0	C C	A C	A C	A G	C T	C T	C C	T T	0 0	T T	T T	0 0
236	0 0	A C	A C	A A	A A	C T	C C	C T	T T	0 0	T T	T T	0 0
237	0 0	C C	A C	A C	A A	T T	T T	C T	T T	0 0	C T	T T	0 0
238	0 0	A C	C C	C C	G G	T T	T T	C C	T T	0 0	T T	C T	0 0
239	0 0	C C	A C	A A	A A	C C	C C	C C	T T	0 0	T T	C C	0 0
240	0 0	A C	C C	A C	A A	C T	C T	C T	G T	0 0	T T	C T	0 0
241	0 0	A C	C C	A C	A G	T T	C T	C C	T T	0 0	T T	T T	0 0
242	0 0	A C	C C	A A	A G	C T	C T	C T	T T	0 0	C T	T T	0 0
243	0 0	A C	A C	A C	A A	T T	C T	C T	T T	0 0	T T	T T	0 0
244	0 0	C C	C C	A C	A G	C T	C T	C C	T T	0 0	T T	T T	0 0
245	0 0	A C	C C	A A	A A	T T	C C	T T	G T	0 0	T T	T T	0 0
246	0 0	A C	C C	A C	A A	C T	C C	C C	G T	0 0	T T	T T	0 0
247	0 0	A C	C C	A C	A G	T T	C T	C T	T T	0 0	T T	T T	0 0
248	0 0	C C	A C	A C	A G	T T	C T	T T	T T	0 0	C T	T T	0 0
249	0 0	A A	C C	A C	A G	T T	T T	C C	G T	0 0	T T	C T	0 0
250	0 0	A C	C C	A C	A G	C T	C T	C T	T T	0 0	T T	T T	0 0
251	0 0	C C	C C	A C	A G	C T	C T	C T	T T	0 0	T T	T T	0 0
252	0 0	A C	A C	A A	A A	C T	C C	C T	T T	0 0	T T	T T	0 0
253	0 0	A C	C C	A A	A A	C C	C C	T T	T T	0 0	T T	T T	0 0
254	0 0	C C	C C	A A	A A	C C	C C	C T	G T	0 0	T T	T T	0 0
255	0 0	C C	C C	A A	A A	C T	C T	C T	G T	0 0	T T	T T	0 0
256	0 0	C C	C C	A C	A G	C T	C T	C C	G G	0 0	T T	T T	0 0
257	0 0	A A	A C	A A	A A	C T	C C	C T	T T	0 0	C C	T T	0 0
258	0 0	A C	A C	A C	A A	T T	T T	C T	G T	0 0	C C	T T	0 0
259	0 0	A C	C C	A A	A A	C C	C C	T T	G T	0 0	T T	T T	0 0
260	0 0	A C	C C	A C	A A	C T	C T	C T	T T	0 0	C T	T T	0 0
261	0 0	C C	C C	A C	A A	C T	C C	C T	G G	0 0	T T	T T	0 0

262	0 0	C C	C C	A C	A G	C T	C T	C C	T T	0 0	C C	C T	0 0
263	0 0	C C	C C	A A	A A	C C	C C	C C	T T	0 0	T T	T T	0 0
264	0 0	A C	C C	A A	A A	C C	C C	T T	G T	0 0	T T	T T	0 0
265	0 0	A C	C C	A A	A A	T T	C T	C C	T T	C C	C T	T T	A A
266	0 0	A A	C C	A A	A A	T T	C C	T T	T T	C T	C T	C T	G G
267	0 0	C C	C C	A C	A A	C T	C C	C C	T T	T T	C C	T T	G G
268	0 0	A C	C C	C C	G G	T T	T T	C T	T T	C C	T T	C T	A G
269	0 0	A C	C C	A A	A A	C C	C C	C T	T T	C C	C C	T T	A A
270	0 0	A C	C C	C C	A G	T T	T T	C T	T T	C T	C T	T T	A A
271	0 0	A C	C C	C C	A G	T T	T T	C C	T T	C C	T T	T T	A A
272	0 0	C C	C C	A C	A G	C T	C T	C C	T T	C T	C T	C T	A G
273	0 0	C C	C C	C C	G G	T T	C T	C T	T T	C T	C C	C T	A A
274	0 0	A C	C C	A A	A A	C T	C C	C T	G T	C C	T T	T T	A A
275	0 0	A C	A C	A A	A A	T T	C C	T T	T T	C C	C T	C T	A G
276	0 0	C C	C C	A A	A A	C T	C C	C T	T T	C C	T T	C T	A G
277	0 0	A C	C C	C C	G G	T T	T T	C C	G T	C C	T T	C T	G G
278	0 0	A A	A C	A A	A A	C T	C T	C C	T T	C C	T T	T T	A A
279	0 0	A A	C C	A A	A A	C C	C C	C T	T T	C C	T T	T T	A G
280	0 0	A C	C C	A A	A A	C C	C C	T T	T T	C C	C T	T T	A A
281	0 0	C C	C C	A C	A A	C T	C T	C T	T T	C C	T T	T T	A A
282	0 0	A C	A C	A C	A G	C T	C T	C C	T T	C C	C T	T T	G G
283	0 0	C C	A C	A A	A A	T T	T T	C C	G T	C C	C T	T T	A A
284	0 0	C C	C C	A C	A G	C T	C T	C C	T T	C C	T T	C T	G G

GENE	HIP1	HIP1	HIP1	HIP1	HIP1	HIP1	HIP1	HIP1	HIP1	HIP1	HIP1	HIP1	HIP1
SNP/Sample	rs794356	rs237238	rs237236	rs807874	rs2705788	rs2240133	rs2240134	rs6957776	rs12533075	rs4385416	rs7457874	rs6945301	rs4620231
1	C T	A A	A G	C T	A T	C T	C C	C G	A G	G G	A A	A A	A A
2	T T	A A	G G	C T	T T	C C	C C	C C	A G	A G	A G	G G	A A
3	C C	A A	G G	C T	A T	C T	C C	C G	A A	A A	G G	A G	A C
4	T T	A A	A G	T T	A T	C T	C C	C C	A A	A A	G G	A G	A A
5	C T	A G	A G	C T	A T	C C	T T	G G	A G	A G	A G	A A	A A
6	C C	A A	G G	C C	T T	T T	C C	C C	A A	A A	G G	A A	A A
7	C T	A A	A G	T T	A T	0 0	C T	C G	A A	A A	G G	G G	A A
8	0 0	A A	0 0	C T	A T	0 0	C C	C C	A A	A A	G G	A A	A C
9	C C	A A	A G	T T	A T	C T	C T	C G	A G	A G	A G	A G	A C
10	C C	A A	G G	0 0	T T	C T	C T	C C	A A	A A	G G	A A	A C
11	C C	A G	A G	T T	A T	C C	T T	G G	G G	G G	A A	A G	A A
12	C C	A G	A G	T T	A T	C C	C T	C G	A G	A G	A G	G G	A A
13	C T	A A	G G	C T	A T	C T	C C	C G	A A	A G	G G	A G	C C
14	T T	A A	A G	T T	A A	C C	T T	G G	G G	G G	A A	A A	A A
15	C T	A A	A G	T T	A T	C T	C T	C G	A G	A G	A G	A G	A A
16	T T	A A	G G	C T	A T	T T	C C	C C	A A	A G	A G	A A	A A
17	C C	A A	G G	T T	T T	C C	C C	G G	A A	A A	G G	G G	A A
18	C T	A A	G G	T T	A A	T T	C C	C C	A A	A G	A G	A G	A A
19	C T	A A	A G	T T	A T	0 0	C C	G G	A A	A A	G G	A G	A A
20	C C	A A	G G	T T	A A	C C	C C	C C	A A	A A	G G	A G	A C
21	C T	A A	A G	T T	A A	T T	C C	C G	A G	A G	A G	A G	A A
22	C C	A G	A A	T T	A T	C T	C T	C C	A A	A A	G G	A A	A C
23	C C	A A	A G	T T	A A	C T	C T	C C	A A	A A	G G	A G	A A
24	C T	A A	G G	T T	A T	C C	C T	C G	A G	A G	A G	A G	C C
25	C C	A A	A G	T T	T T	C C	C C	G G	A A	A G	G G	G G	C C
26	T T	A A	G G	C T	A A	C C	T T	G G	G G	G G	A A	A A	A A
27	C C	A A	A G	T T	A T	C C	C T	C G	A G	A G	A G	A G	A C
28	C T	A A	G G	C T	A A	C C	C C	G G	A G	A G	G G	A G	A C
29	T T	A A	A A	T T	T T	C C	C C	C C	A A	A A	G G	G G	A A
30	T T	A A	A G	T T	A T	C T	C T	C G	A G	A A	G G	A G	A A

31	C T	A A	A G	T T	A A	C C	C C	C C	A A	A A	G G	A A	A A
32	C C	A G	A A	T T	A A	00	C C	C G	A A	A A	G G	A G	A A
33	C T	A A	G G	C T	A T	C T	C T	G G	A G	A A	G G	G G	A A
34	C T	A A	G G	T T	A A	C C	C T	G G	A G	G G	A G	A G	A A
35	C C	A A	G G	00	A T	C T	C T	C G	A G	A G	A G	A G	A A
36	C C	A A	G G	C C	T T	C T	C T	C G	A G	A A	G G	A G	A C
37	00	A A	G G	00	T T	C C	T T	G G	00	A A	G G	G G	A A
38	C T	A A	A A	T T	A A	C T	C T	C G	A G	A G	A G	A A	A A
39	00	A G	00	00	00	00	C C	C G	00	A G	G G	G G	00
40	C C	A A	G G	C C	T T	C T	C T	C G	A G	A A	G G	A G	A A
41	00	A A	00	00	00	00	C C	C G	00	A G	G G	A G	00
42	C T	A A	G G	T T	A A	C T	C C	C C	A A	A A	G G	A G	A A
43	00	A A	00	00	00	00	C T	C G	00	A G	A G	A G	00
44	C C	A A	G G	C C	T T	00	T T	G G	G G	A G	A G	G G	A A
45	00	A G	A G	00	A T	C C	T T	G G	G G	G G	00	A G	A A
46	C T	A A	A G	T T	A A	C T	C T	C C	A A	A A	G G	A A	A A
47	00	A A	G G	00	A A	T T	C C	C C	A A	A A	G G	A A	A A
48	C C	A A	G G	T T	T T	C T	C C	C C	A A	A A	G G	A G	A A
49	C T	A A	G G	T T	A T	C C	C T	C G	A G	A G	A G	A G	A A
50	00	A A	00	00	00	00	C T	C G	00	A A	G G	G G	00
51	C T	A A	G G	C T	A T	C T	C C	C C	A A	A A	G G	A G	A A
52	00	A A	00	00	00	00	C C	C G	00	A A	G G	A G	00
53	C T	A G	A A	T T	A T	C T	C T	C G	A G	A G	A G	A G	A A
54	00	A A	A G	00	A T	C T	C T	C G	A A	A A	G G	A G	00
55	C T	A A	G G	T T	A A	C C	C C	C C	A A	A A	G G	A A	A C
56	00	A A	G G	00	A T	00	C T	C G	00	A G	A G	A G	00
57	C T	A G	A G	T T	A T	C T	C C	C C	A A	A A	G G	A A	A C
58	T T	A A	G G	T T	A A	C T	C T	C G	A G	A G	A G	A G	A A
59	C C	A A	A G	C T	A A	T T	C C	C C	A A	A A	G G	A A	A A
60	C T	A A	G G	C T	A T	C T	C C	C C	A A	A A	G G	A G	A A
61	T T	A A	A G	T T	A T	C C	C T	C G	A G	A G	A G	A A	A C
62	00	A A	A G	00	A T	C T	C C	C C	A A	A A	G G	A G	A C
63	C C	A A	G G	T T	T T	C C	C T	G G	A G	A G	A G	G G	A C

64	C T	A A	A G	T T	A A	C T	0 0	0 0	A A	A A	G G	A A	A C
65	C T	A A	G G	T T	A T	C T	C T	C G	A G	A G	A G	A G	A A
66	0 0	A A	G G	0 0	T T	C T	0 0	C G	A A	A G	G G	G G	A C
67	C T	A A	A G	T T	A A	C C	C C	C G	A G	A G	A G	A A	A A
68	C C	A A	A G	C T	A T	0 0	T T	G G	G G	G G	A A	G G	A A
69	C C	A A	A G	T T	A T	C T	C T	C G	0 0	A G	A G	A G	A A
70	C T	A A	G G	C T	T T	C C	0 0	G G	A G	G G	A G	G G	A A
71	T T	A A	G G	T T	A T	C C	C C	C C	A A	A A	G G	G G	A A
72	C C	A A	G G	T T	A T	C T	C C	C C	A A	A G	A G	G G	A A
73	C C	A A	G G	C C	A T	C C	C T	G G	A G	A G	A G	G G	A A
74	C C	A A	G G	C C	T T	C T	C T	C G	A G	A G	A G	A G	A C
75	C T	A A	A A	T T	A T	C T	C T	C G	A G	A G	A G	A G	A A
76	C T	A A	G G	T T	A A	T T	C C	C C	A A	A A	G G	A A	A A
77	C C	A A	G G	T T	T T	C C	C C	C G	A A	A A	G G	G G	A A
78	T T	A A	G G	T T	A A	C T	C C	C G	A A	A G	G G	A G	A A
79	C T	A A	G G	T T	A T	C C	C C	C C	A A	A A	G G	A G	A A
80	C T	A G	A A	T T	A T	0 0	C C	C G	A G	A A	G G	A G	A C
81	C T	G G	A A	T T	T T	C C	C T	C G	A G	A G	A G	G G	A A
82	T T	A A	A G	T T	A A	C C	T T	G G	G G	G G	A G	A A	A A
83	C C	A A	G G	C T	T T	C T	C C	C C	A A	A A	G G	A G	A A
84	C T	A A	G G	C T	A T	C T	C C	C C	A A	A A	G G	A G	A A
85	C C	A A	G G	C T	T T	C C	C C	C G	A A	A A	G G	G G	A C
86	C T	A A	A G	T T	A A	T T	C C	C C	A A	A A	G G	A A	A A
87	C T	A A	A A	T T	A T	0 0	T T	G G	G G	G G	A A	A A	A A
88	CT	AA	GG	CT	AA	CT	CC	CG	AG	AA	GG	AA	AC
89	C C	0 0	A A	0 0	T T	C C	0 0	0 0	A G	0 0	G G	0 0	A C
90	T T	A A	A G	C T	A T	C C	C T	G G	A G	A G	A G	G G	A A
91	C T	A A	G G	T T	A T	C T	C C	C C	A A	A A	G G	A A	A A
92	T T	A A	A G	T T	A A	T T	C C	C C	A A	A A	G G	A A	A A
93	C C	A A	A G	T T	T T	C C	C C	C G	A A	A A	G G	G G	A A
94	C T	A A	G G	C C	T T	C C	C T	G G	A G	A G	G G	G G	A A
95	C C	A A	A G	T T	A A	C T	C T	G G	A G	A G	A G	A G	A A
96	C T	A A	A A	T T	A T	C C	T T	G G	G G	G G	A A	0 0	A A

97	C T	A G	A A	T T	A A	C C	C T	C G	A G	A G	A G	A A	A A
98	T T	A A	G G	T T	A T	C T	C C	C C	A A	A A	G G	A G	A A
99	C T	A A	G G	C T	A T	C T	C T	C G	A G	A G	A G	A A	A C
100	0 0	0 0	0 0	0 0	0 0	0 0	C T	G G	0 0	A G	A G	A G	0 0
101	C T	A A	A G	C T	A A	T T	C C	C C	A A	A A	G G	A A	A A
102	C T	A A	G G	C T	A A	C C	C C	C G	A A	A G	G G	G G	A A
103	C T	A A	A G	T T	A A	C T	C T	G G	G G	G G	A A	A G	A A
104	C T	A A	G G	T T	T T	C C	C T	C G	A G	A G	A G	A G	A C
105	C T	A G	A A	T T	A A	C C	C T	G G	A G	A G	A G	A G	A A
106	C T	A A	A G	T T	A T	C C	C T	C C	A A	A A	G G	G G	A A
107	C T	A A	A G	T T	A A	T T	C C	C G	A A	A A	G G	A A	A A
108	C T	A G	A A	T T	T T	C C	C C	C C	A A	A A	G G	A G	A A
109	C T	A A	G G	C T	A A	C C	T T	C G	A G	A G	A G	A A	A A
110	T T	A A	G G	T T	A T	C C	C T	C G	A G	A A	G G	A G	A C
111	C C	A A	G G	C T	A T	C C	C T	G G	G G	A G	A G	A G	C C
112	C T	A A	A G	C T	T T	C T	C C	C C	A A	A A	G G	A G	A C
113	C T	A A	A G	T T	A T	C T	C T	C G	A G	G G	A G	G G	A C
114	C C	A A	A A	T T	A T	C C	C T	C G	A A	A A	G G	A G	A A
115	C T	A A	G G	C T	A A	C T	C C	C C	A A	A A	G G	A G	A A
116	C C	A A	A G	T T	A A	C C	T T	C G	A G	A G	A G	G G	A A
117	C C	A A	G G	C T	A T	C T	C C	C G	A A	A A	G G	G G	A C
118	C T	A A	A G	T T	A T	C T	C T	C G	A G	A G	A G	A G	A A
119	C T	A A	G G	C T	T T	C T	C T	C G	A G	A G	A G	A G	A C
120	C C	A A	G G	C T	A A	C T	C T	C G	A G	A A	G G	A A	A A
121	C C	A A	A A	T T	T T	C C	C C	G G	A A	A G	G G	G G	A C
122	C T	A A	A G	T T	T T	C T	C T	C G	A G	A G	A G	A A	A C
123	C C	A A	A A	T T	A T	C C	T T	G G	G G	G G	A A	A A	A A
124	C C	A A	G G	T T	A A	C T	C T	C G	G G	G G	A A	A G	A A
125	0 0	A A	A A	0 0	A A	C T	C C	C G	0 0	A A	G G	A A	A C
126	C C	A A	G G	C T	A T	C C	C C	G G	A G	A A	G G	A G	A C
127	C C	A A	G G	C T	A T	C C	C T	G G	A G	G G	A G	A G	A C
128	T T	A A	G G	T T	A A	T T	C C	C C	A A	A A	G G	A A	A A
129	0 0	A A	A G	0 0	A T	C T	C T	0 0	0 0	A A	G G	0 0	A C

130	C T	A G	A G	T T	A A	C T	C T	C G	A G	A G	A G	A A	A A
131	0 0	A G	A G	0 0	A A	C T	C T	C G	0 0	A G	A G	A A	A C
132	C C	A A	G G	C C	T T	C C	C T	G G	A G	A G	A G	G G	A A
133	C C	0 0	A G	T T	A T	C C	T T	G G	G G	G G	0 0	0 0	A A
134	T T	A A	A G	T T	T T	C C	C C	C G	A A	A A	G G	G G	A A
135	0 0	A A	G G	0 0	T T	C T	C T	C G	0 0	A G	A G	A G	A C
136	C C	A G	A A	T T	A A	C C	T T	G G	G G	G G	A A	A A	A C
137	C T	A A	G G	C T	A A	C T	C C	C G	A A	A G	G G	A G	A C
138	C C	A A	G G	C T	A T	C T	C C	C C	A A	A A	G G	A A	A C
139	C T	A A	G G	T T	T T	C T	C C	C G	A A	A G	G G	A G	A C
140	C T	A A	G G	C T	A T	C C	T T	C G	A G	A G	A G	G G	A A
141	T T	A A	A G	T T	A T	C T	C C	C C	A A	A A	G G	A G	A A
142	C T	A A	A G	C T	A T	C T	C T	C G	G G	G G	A A	G G	A A
143	T T	A A	G G	C C	A T	C C	C T	C G	A G	A G	A G	G G	A A
144	C C	A A	A G	C T	A A	C T	C T	C C	A A	A A	G G	A G	A C
145	C T	A A	A G	C T	A T	C C	C C	C G	A G	A A	G G	A G	A C
146	T T	A G	A G	T T	A T	C C	C T	C G	A G	A G	A G	A G	A A
147	C T	0 0	A G	T T	A A	T T	C C	C C	A A	A A	0 0	A A	A A
148	T T	A A	A G	T T	A A	C T	C T	C G	A G	A G	A G	A A	A C
149	T T	A A	A G	C T	A T	C C	C T	G G	A G	A G	A G	A G	A C
150	0 0	A A	G G	0 0	A T	C T	C C	G G	0 0	A G	G G	G G	0 0
151	C T	A A	G G	C T	T T	C C	C T	G G	A G	G G	A G	G G	A C
152	C C	A A	G G	T T	T T	C C	C T	C G	A G	A G	A G	G G	A A
153	C C	A A	A A	T T	A T	C T	C C	C G	A A	A G	G G	A G	A A
154	C T	A A	G G	C T	A T	C C	C T	0 0	A G	0 0	G G	0 0	A A
155	C T	A A	A G	C T	A T	C C	T T	G G	G G	G G	A G	A G	A A
156	C T	A A	G G	T T	T T	C C	C T	C G	A G	A G	A G	G G	A A
157	C C	A G	A A	T T	A T	C T	C C	C G	A G	A G	A G	A G	A C
158	0 0	A A	G G	0 0	A T	C C	T T	G G	0 0	A G	A G	0 0	A A
159	C T	A A	G G	T T	T T	C C	C T	G G	A G	A A	G G	G G	A A
160	0 0	A A	0 0	0 0	0 0	0 0	C C	0 0	0 0	A A	G G	0 0	0 0
161	T T	A A	A G	T T	T T	C C	C C	C G	A A	A A	G G	G G	A A
162	C C	A A	A G	T T	T T	C C	C T	C G	A G	A G	A G	G G	A A

163	T T	A A	G G	C T	T T	C C	C C	C G	A A	A G	G G	G G	C C
164	C C	A A	G G	C C	A T	C C	C T	G G	G G	A G	A G	A G	A C
165	C C	A A	A G	C T	A T	C C	C C	G G	A G	A A	G G	A A	A C
166	C T	A A	G G	C C	A T	C T	C C	C G	A A	A G	G G	A G	A A
167	C T	A A	A G	T T	A T	C T	C C	C C	A A	A A	G G	A G	A A
168	C C	A A	A G	T T	T T	C C	T T	G G	G G	G G	A A	G G	A A
169	C C	A G	A G	T T	T T	C C	C C	C G	A A	A G	G G	G G	A C
170	C C	A A	G G	C C	T T	C C	C T	G G	A G	G G	A G	G G	A C
171	C T	A A	G G	T T	A T	C T	C T	C G	A G	A G	A G	A G	A A
172	C T	A A	G G	C T	A T	T T	0 0	0 0	A A	0 0	G G	0 0	A C
173	C T	A A	G G	T T	A T	C T	C C	C G	A A	A A	G G	A G	A C
174	C T	A A	A A	T T	T T	C C	T T	G G	G G	G G	A A	G G	A A
175	C T	A A	G G	C T	A T	C C	C T	G G	G G	A G	A G	A G	A A
176	C C	A A	A G	T T	A A	T T	C C	C C	A A	A A	G G	A A	A A
177	0 0	A A	G G	0 0	A T	C T	C T	C G	A G	A G	A G	A G	A A
178	0 0	A A	A G	0 0	A A	T T	C C	G G	A A	A G	G G	A G	A C
179	0 0	A A	A G	0 0	A T	C C	T T	G G	G G	G G	A A	0 0	A A
180	0 0	A G	A G	0 0	A T	C T	C C	C C	A A	A A	G G	A G	A A
181	0 0	A A	G G	0 0	T T	C C	C T	C G	A G	A G	A G	0 0	A A
182	0 0	A A	G G	0 0	T T	C T	C T	C G	A G	A A	G G	A G	A C
183	0 0	A A	A G	0 0	A T	C C	C T	G G	A G	A G	A G	G G	A A
184	0 0	A A	A A	0 0	T T	C C	C T	G G	G G	A G	A G	A G	A C
185	0 0	0 0	A A	0 0	T T	C C	T T	G G	G G	G G	0 0	G G	A A
186	0 0	G G	A A	0 0	A T	C C	C T	C G	A G	A G	A G	A G	A C
187	0 0	A A	G G	0 0	T T	C C	C T	G G	A G	G G	A G	G G	A C
188	0 0	A A	G G	0 0	A A	C T	C T	C G	A G	A G	A G	A A	A C
189	0 0	A A	A G	0 0	A T	C T	C C	C G	A G	A G	A G	0 0	A C
190	0 0	A A	G G	0 0	A A	T T	C C	C C	A A	A A	G G	A A	A A
191	0 0	A A	A G	0 0	A A	C T	C T	C G	G G	G G	A A	A A	A C
192	0 0	A G	A G	0 0	A T	C T	C C	C C	A A	A A	G G	A A	A C
193	0 0	A A	G G	0 0	A A	C T	C T	C G	A G	A G	A G	A G	A C
194	0 0	A A	G G	0 0	A T	C C	T T	C G	A G	A G	A G	G G	A A
195	0 0	A A	A G	0 0	T T	C C	C C	C G	A A	A G	G G	G G	A A

196	0 0	A A	A G	0 0	A T	C C	C C	C C	A A	A A	G G	G G	A A
197	0 0	A A	A G	0 0	A A	T T	C C	C C	A A	A A	G G	A A	A C
198	0 0	A A	A G	0 0	A A	T T	0 0	0 0	A A	A A	G G	A A	A A
199	0 0	A A	A G	0 0	A A	T T	C C	C C	A A	A A	G G	A A	A A
200	0 0	A A	A G	0 0	A T	C C	C C	C G	A A	A A	G G	A G	A C
201	0 0	A A	A G	0 0	A T	C C	C T	C G	A G	A G	A G	A G	A A
202	0 0	A A	G G	0 0	A T	C C	T T	G G	G G	G G	A A	A G	A A
203	0 0	A A	G G	0 0	A T	C C	T T	G G	G G	G G	A A	A G	A A
204	0 0	A A	A G	0 0	A T	C C	T T	G G	G G	A G	A G	A G	A A
205	0 0	A A	G G	0 0	A T	C C	T T	G G	G G	A G	A G	A G	A C
206	0 0	A A	A A	0 0	A A	C T	C C	G G	G G	A G	A G	A A	A C
207	0 0	A A	G G	0 0	A T	C C	C T	G G	A G	A G	A G	A G	A A
208	0 0	A G	A G	0 0	A T	C T	C C	C C	A A	A A	G G	A G	A C
209	0 0	A G	A G	0 0	A T	C T	C C	C C	A A	A A	G G	A G	A C
210	0 0	A A	G G	0 0	A A	T T	C C	C C	A A	A A	G G	A A	A A
211	0 0	A A	G G	0 0	A T	C T	0 0	C G	A A	A G	G G	A G	A A
212	0 0	A A	G G	0 0	A T	C T	0 0	C G	A A	A G	G G	A G	A C
213	0 0	A A	A G	0 0	T T	C C	T T	G G	G G	G G	A A	G G	A A
214	0 0	A A	A G	0 0	A T	C C	T T	C G	A G	A G	A G	G G	A A
215	0 0	A A	G G	0 0	A A	C C	C T	C G	A A	A G	A G	A A	A A
216	0 0	A A	G G	0 0	A A	T T	C C	C C	A A	A A	G G	A A	A A
217	0 0	A A	G G	0 0	T T	C T	C T	G G	G G	G G	A A	A G	A A
218	0 0	A A	A G	0 0	A A	T T	C C	C C	A A	A A	G G	A A	A A
219	0 0	A A	A G	0 0	A T	C T	C T	C G	A G	A G	A G	A A	A A
220	0 0	A G	A G	0 0	A T	C C	C T	C G	A G	A G	A G	A G	A A
221	0 0	A A	G G	0 0	T T	C C	T T	G G	A G	A G	A G	A G	A A
222	0 0	A A	G G	0 0	A T	T T	C C	C C	A A	A A	G G	A A	A C
223	0 0	A G	A G	0 0	T T	C C	C T	C G	A G	A G	A G	G G	C C
224	0 0	A A	A G	0 0	A T	T T	C T	C G	A G	A G	A G	0 0	A A
225	0 0	A A	G G	0 0	A A	C T	C T	C G	A G	A G	A G	A A	A A
226	0 0	A A	G G	0 0	T T	C C	C C	C G	A A	A G	G G	A G	A C
227	0 0	A A	A G	0 0	A A	C T	C T	C C	A A	A A	G G	A A	A A
228	0 0	A A	G G	0 0	A T	C T	C C	G G	A A	A A	G G	G G	A A

229	0 0	A A	G G	0 0	A A	C T	C C	C C	A A	A A	G G	A A	A A
230	0 0	A A	G G	0 0	A A	T T	C C	C C	A G	A A	G G	A G	A A
231	0 0	A A	G G	0 0	T T	C C	T T	G G	G G	G G	A A	G G	A A
232	0 0	A A	A G	0 0	T T	C C	C T	C G	A G	A G	A G	G G	A A
233	0 0	A A	G G	0 0	A A	C T	C T	C G	A A	A A	G G	A G	A A
234	0 0	A A	G G	0 0	A A	T T	C C	C C	A A	A A	G G	A A	C C
235	0 0	A A	G G	0 0	A T	C T	C C	C C	A A	A A	G G	A G	A C
236	0 0	A A	G G	0 0	A A	T T	C C	C C	A A	A A	G G	A A	A A
237	0 0	A A	G G	0 0	A A	C T	0 0	C G	A G	A G	A G	A A	A A
238	0 0	A A	G G	0 0	A T	C C	T T	G G	A G	A A	G G	G G	A C
239	0 0	G G	A A	0 0	A A	C C	T T	G G	G G	G G	A A	A A	A A
240	0 0	A A	A G	0 0	A A	C C	C T	C G	A G	A G	A G	A G	A A
241	0 0	A A	G G	0 0	A A	T T	C C	C C	A A	A A	G G	A A	A A
242	0 0	A A	A G	0 0	A T	C T	C T	C G	A G	A G	A G	A G	A C
243	0 0	A A	G G	0 0	A T	C T	C T	C G	A G	A G	A G	A G	A A
244	0 0	A A	A G	0 0	A A	C C	C T	C C	A A	A G	G G	G G	A A
245	0 0	A A	G G	0 0	A T	C C	C T	C G	A G	A G	A G	G G	A A
246	0 0	A A	G G	0 0	A T	C C	C C	C C	A A	A A	G G	A G	A A
247	0 0	A G	A A	0 0	T T	C C	C T	G G	A G	A G	A G	A A	A A
248	0 0	A G	A G	0 0	A T	C T	C C	C C	A A	A A	G G	A A	A C
249	0 0	A A	A G	0 0	A A	C T	C T	C G	A G	A G	A G	A G	A C
250	0 0	A A	A G	0 0	A A	C C	T T	G G	G G	G G	A A	G G	A A
251	0 0	A A	A G	0 0	A A	C T	C T	C G	A G	A G	A G	A A	A C
252	0 0	A A	A G	0 0	A T	T T	C C	C C	A A	A G	A G	A G	A C
253	0 0	A G	A A	0 0	A T	C C	C C	G G	A A	A A	G G	G G	A A
254	0 0	A A	A G	0 0	A T	C T	C C	C G	A G	A A	G G	A A	C C
255	0 0	A A	A G	0 0	A T	C C	T T	G G	G G	G G	A A	G G	A A
256	0 0	A A	G G	0 0	A T	C C	C T	G G	G G	A G	A G	A G	A C
257	0 0	A G	A G	0 0	A T	C T	C T	C G	A G	A G	A G	A G	A A
258	0 0	A A	G G	0 0	A A	C T	C C	C C	A A	A A	G G	A A	A C
259	0 0	A A	G G	0 0	A T	C T	C C	C G	A A	A G	G G	A G	A A
260	0 0	A A	A G	0 0	A A	C T	0 0	C C	A A	A A	G G	A A	A A
261	0 0	A A	A G	0 0	A A	C T	0 0	0 0	A A	0 0	G G	0 0	A C

262	0 0	A A	G G	0 0	A T	C C	C T	C G	A G	A G	A G	A G	A A
263	0 0	A G	A G	0 0	A T	C T	C C	C G	A A	A G	G G	A G	A A
264	0 0	A A	A A	0 0	A A	C T	C T	C C	A A	A A	G G	A G	A C
265	T T	A A	G G	C T	A T	C C	T T	G G	G G	G G	A A	A G	A A
266	C C	A G	A G	T T	A T	C C	T T	G G	G G	G G	A A	A G	A A
267	C C	A A	A G	T T	A T	C T	C C	C C	A A	A A	G G	A G	A A
268	C T	A A	G G	C C	T T	C T	C T	C G	A G	A G	A G	A G	A C
269	T T	A A	G G	T T	A A	C T	C C	C C	A A	A A	G G	A A	A A
270	C T	A A	G G	C T	A A	T T	C C	C C	A A	A A	G G	A A	A C
271	T T	A G	A G	T T	A T	C C	T T	G G	G G	A G	A G	A G	A A
272	C T	A G	A A	T T	A T	C C	T T	G G	G G	G G	A A	A G	A A
273	C T	A A	A G	C T	A A	C C	C C	G G	A G	A A	G G	A G	A A
274	T T	A A	G G	C T	A T	C C	C T	C G	A G	A G	A G	G G	A A
275	C T	A A	G G	C T	A T	C T	C T	C C	A A	A A	G G	A A	A A
276	C T	A A	A G	T T	T T	C C	0 0	0 0	A A	0 0	G G	0 0	A A
277	C C	A A	G G	C T	T T	C T	C C	C G	A A	A G	G G	A G	C C
278	C T	A A	G G	T T	A A	C T	C T	G G	A G	A G	A G	A G	A A
279	C T	A A	A G	C T	T T	C T	C C	C G	A A	A G	G G	A G	C C
280	C T	A A	A G	C T	T T	C C	C T	C G	A G	A G	A G	G G	A A
281	T T	A A	G G	T T	A A	C T	C T	C C	A A	A A	G G	A G	A A
282	C C	A A	A G	T T	A A	T T	C C	C G	A G	A G	A G	A G	A A
283	C T	A A	A G	T T	A T	C T	C C	C C	A A	A A	G G	A G	A A
284	C C	A G	A G	C T	A T	C C	C T	C C	A A	A A	G G	G G	A A

GENE	HIP1	HIP1	HIP1	CCL26	CCL26	CDK5	CDK5	CDK5	SIRT1	SIRT1	SIRT1	SIRT1	SIRT1
SNP/Sample	rs9649625	rs12538253	rs10275942	rs11465353	rs2868166	rs9278	rs2069459	rs2069443	rs17712705	rs12778366	rs10997866	rs10997870	rs7091896
1	C C	C T	T T	T T	C C	G G	G G	C C	A G	C T	A G	G T	C G
2	A A	T T	C C	G T	A C	G G	G G	A C	G G	T T	G G	T T	G G
3	C C	C T	T T	T T	A C	G G	G T	A C	A A	C T	A A	G G	C C
4	C C	T T	T T	T T	C C	G G	T T	A A	G G	T T	G G	T T	G G
5	A C	C T	C T	G T	A C	A G	G G	A A	G G	T T	G G	T T	G G
6	C C	C T	T T	T T	C C	A G	G G	A C	A G	C T	A G	G T	C G
7	C C	C C	T T	T T	C C	G G	G T	A C	A G	T T	A G	G T	C G
8	C C	C C	T T	T T	A C	A G	G G	A C	G G	0 0	G G	0 0	G G
9	A C	T T	C T	G T	A A	G G	G T	A C	G G	T T	G G	T T	G G
10	C C	C T	T T	T T	0 0	A A	G G	A A	A G	C T	A G	G T	C G
11	A C	C T	C T	G T	A C	G G	G G	C C	A G	T T	A G	G T	C G
12	C C	0 0	T T	T T	C C	0 0	0 0	A C	G G	T T	G G	0 0	G G
13	C C	C T	T T	T T	A A	G G	T T	A A	G G	T T	G G	T T	G G
14	A A	T T	C C	G G	A A	A G	G G	A C	G G	T T	G G	T T	G G
15	A C	C T	C T	G T	A C	A G	G G	A A	A G	C T	A G	G T	C G
16	C C	T T	T T	T T	C C	G G	G G	A C	A G	C T	A G	G T	C G
17	A C	T T	C T	G T	A C	A G	G T	A A	A G	C T	A G	G T	C G
18	C C	C T	T T	T T	A C	G G	G T	A A	G G	T T	G G	T T	G G
19	A C	C T	C T	G T	A C	A G	G G	A A	A G	T T	A G	G T	C G
20	C C	C C	T T	T T	A C	G G	T T	A A	A A	C C	A A	G G	C C
21	C C	C T	T T	T T	C C	G G	T T	A A	A G	T T	A G	G T	C G
22	C C	T T	T T	T T	A C	G G	T T	A A	A G	C T	A G	G T	C G
23	C C	C T	T T	T T	C C	A G	G G	A A	A G	T T	A G	G T	C G
24	C C	T T	T T	T T	A A	G G	T T	A A	G G	T T	G G	T T	G G
25	C C	T T	T T	T T	A A	G G	G T	A A	G G	T T	G G	T T	G G
26	A A	T T	0 0	G G	A A	G G	G T	A C	G G	T T	G G	T T	G G
27	C C	C T	T T	T T	A C	A G	G G	A A	G G	T T	G G	T T	G G
28	C C	T T	T T	T T	A C	G G	G G	A A	G G	T T	G G	T T	G G
29	C C	T T	T T	T T	C C	G G	G G	A C	G G	T T	A G	G T	G G
30	C C	C C	T T	T T	C C	G G	G T	A C	G G	T T	G G	T T	G G

31	A C	C T	C T	G T	A C	G G	T T	A A	G G	T T	G G	T T	G G
32	C C	C T	T T	T T	C C	A G	G G	A C	G G	T T	G G	T T	G G
33	C C	C T	T T	T T	C C	G G	G T	A C	G G	T T	G G	T T	G G
34	A C	C T	C T	G T	A C	G G	G T	A A	G G	T T	G G	T T	G G
35	A C	C T	C T	G T	A C	A G	G T	A A	G G	T T	G G	T T	G G
36	C C	C T	T T	T T	A C	G G	G G	C C	A G	C T	A G	G T	C G
37	C C	0 0	T T	T T	C C	0 0	0 0	C C	0 0	0 0	0 0	0 0	G G
38	A C	C T	C T	G T	A C	A G	G G	A C	A G	T T	A G	G T	C G
39	C C	0 0	T T	T T	C C	0 0	0 0	0 0	0 0	0 0	0 0	0 0	0 0
40	A C	T T	C T	G T	A C	A G	G G	A A	G G	T T	G G	T T	G G
41	C C	0 0	T T	T T	A C	0 0	0 0	0 0	0 0	0 0	0 0	0 0	0 0
42	A C	T T	C T	G T	A C	G G	G T	A A	A G	C T	A G	G T	C G
43	C C	0 0	T T	T T	A C	0 0	0 0	0 0	0 0	0 0	0 0	0 0	0 0
44	C C	C T	T T	T T	C C	G G	G T	A C	A G	T T	A G	G T	C G
45	C C	0 0	T T	T T	C C	0 0	0 0	A C	0 0	T T	0 0	0 0	C C
46	A A	T T	C C	G T	A C	G G	G T	A C	A G	C T	A G	G T	C G
47	A A	T T	C C	G G	A A	0 0	0 0	A A	G G	T T	0 0	0 0	G G
48	C C	C T	0 0	T T	C C	G G	G G	C C	G G	T T	G G	T T	G G
49	A C	C T	C T	G T	A C	G G	G G	A C	A G	T T	A G	G T	C G
50	A C	0 0	C T	G T	A C	0 0	0 0	0 0	0 0	0 0	0 0	0 0	0 0
51	A C	C T	C T	G T	A C	G G	G G	C C	G G	T T	G G	T T	G G
52	A C	0 0	C T	T T	C C	0 0	0 0	0 0	0 0	0 0	0 0	0 0	0 0
53	A C	C T	C T	G T	A C	G G	G T	A A	G G	T T	G G	T T	G G
54	C C	C C	T T	T T	C C	0 0	0 0	A A	0 0	0 0	0 0	0 0	0 0
55	C C	C T	T T	T T	A C	G G	G G	A C	A G	C T	A G	G T	C G
56	C C	0 0	T T	T T	C C	0 0	0 0	A A	0 0	0 0	0 0	0 0	0 0
57	C C	C T	T T	T T	C C	A G	G G	A C	A G	C T	A G	G T	C G
58	A A	T T	C C	G G	A A	G G	T T	A A	G G	T T	G G	T T	G G
59	C C	C C	T T	T T	C C	G G	G G	A C	A G	C T	A G	G T	C G
60	C C	C C	T T	T T	C C	0 0	G T	A A	G G	T T	G G	T T	G G
61	C C	C T	T T	T T	A C	G G	G T	A C	A G	T T	A G	G T	C G
62	A C	0 0	C T	G T	A C	0 0	0 0	A A	G G	0 0	0 0	0 0	G G
63	A C	T T	C T	G T	A A	A G	G T	A A	G G	T T	G G	T T	G G

64	A C	C T	T T	0 0	A A	G G	G G	C C	G G	T T	G G	T T	G G
65	C C	C C	T T	T T	C C	G G	G G	C C	A G	T T	A G	G T	C G
66	C C	0 0	T T	0 0	A C	A A	0 0	A A	0 0	0 0	0 0	0 0	0 0
67	A C	C T	C T	G T	A C	G G	G G	C C	A G	C T	A G	G T	C G
68	A C	C C	C T	G T	A C	A G	G T	A A	A A	T T	A A	G G	C C
69	C C	T T	T T	T T	C C	G G	T T	A A	G G	T T	G G	T T	G G
70	A A	T T	C C	G G	A A	G G	G T	A A	G G	T T	G G	T T	G G
71	A C	T T	C T	G T	A C	G G	G T	A A	G G	T T	G G	T T	G G
72	C C	T T	T T	T T	C C	G G	G G	A C	A G	T T	A G	G T	C G
73	C C	C T	T T	T T	C C	G G	G G	C C	G G	T T	G G	T T	G G
74	C C	T T	T T	T T	C C	G G	T T	A A	A G	T T	A G	G T	C G
75	A C	C T	C T	G T	A C	G G	G T	A C	G G	T T	G G	T T	G G
76	A C	C T	C T	G T	A C	A G	G T	A A	G G	T T	G G	T T	G G
77	C C	C T	T T	T T	C C	G G	G G	A A	A G	T T	A G	G T	C G
78	A C	C T	C T	G T	A C	A A	G G	A A	A A	C T	A A	G G	C C
79	C C	T T	T T	T T	C C	A G	G T	A A	A G	T T	A G	G T	C G
80	C C	C T	T T	T T	A C	G G	G G	A C	A G	T T	A G	G T	C G
81	A C	C T	C T	G T	A C	G G	G G	C C	G G	T T	G G	T T	G G
82	A C	C T	C T	G T	A C	G G	G T	A C	G G	T T	G G	T T	G G
83	A C	C T	C T	G T	A C	G G	G T	A A	G G	T T	G G	T T	G G
84	C C	C T	T T	T T	C C	A G	G G	A A	A G	C T	A G	G T	C G
85	C C	C T	T T	T T	A C	G G	T T	A A	G G	T T	G G	T T	G G
86	C C	C C	T T	T T	C C	G G	G G	A C	G G	T T	G G	T T	G G
87	A A	C T	C C	G G	A A	G G	G T	A C	G G	T T	G G	T T	G G
88	CC	00	TT	TT	AC	AG	GG	AC	GG	TT	GG	TT	GG
89	0 0	T T	0 0	0 0	0 0	A G	G T	A C	G G	0 0	G G	T T	G G
90	A C	C T	0 0	G T	A C	G G	G T	A A	A G	T T	A A	G G	C G
91	C C	C T	T T	T T	C C	A G	G T	A A	A G	T T	0 0	0 0	C G
92	A C	C T	C T	G T	A C	A G	G G	A C	G G	T T	A G	G T	G G
93	A C	T T	0 0	G T	A C	A G	G T	A A	G G	T T	G G	T T	G G
94	C C	C T	T T	T T	C C	A G	G T	A A	A A	T T	A A	G G	C C
95	A C	C T	0 0	G T	A C	G G	G T	A C	G G	T T	G G	T T	G G
96	A C	C T	C T	G T	A C	G G	G T	A C	A G	T T	A G	G T	C G

97	A C	C T	0 0	G T	A C	G G	G G	A C	G G	T T	G G	T T	G G
98	A C	C T	0 0	G T	A C	G G	G T	A A	A A	C C	A A	G G	C C
99	C C	T T	T T	T T	A C	G G	G G	A C	A G	C T	A G	G T	C G
100	C C	0 0	T T	T T	A C	0 0	0 0	0 0	0 0	0 0	0 0	0 0	0 0
101	A C	C T	C T	G T	A C	A G	G G	A C	A G	T T	A G	G T	C G
102	C C	T T	T T	T T	C C	G G	G G	A C	A G	C T	A G	G T	C G
103	A C	T T	C T	G T	A C	G G	G G	A C	A A	C T	A A	G G	C C
104	C C	C T	T T	T T	A C	G G	G T	A C	G G	T T	G G	T T	G G
105	A C	C T	C T	T T	C C	G G	G T	A C	A A	C T	A A	G G	C C
106	C C	T T	T T	T T	C C	G G	G T	A C	G G	T T	G G	T T	G G
107	C C	C C	T T	T T	C C	G G	G T	A A	A G	T T	A G	G T	C G
108	C C	C T	T T	T T	C C	A G	G G	A A	G G	T T	G G	T T	G G
109	A C	T T	C T	G T	A C	G G	G G	A C	A G	T T	A G	G T	C G
110	C C	C T	T T	T T	A C	A G	G T	A A	G G	T T	G G	T T	G G
111	C C	T T	T T	T T	A A	G G	G T	A A	G G	T T	G G	T T	G G
112	C C	T T	T T	T T	A C	A G	G G	A C	A G	T T	A G	G T	C G
113	A C	T T	C T	G T	A A	G G	T T	A A	G G	T T	G G	T T	G G
114	C C	C T	T T	T T	C C	G G	G T	A A	G G	T T	G G	T T	G G
115	A C	T T	C T	G T	A C	G G	G T	A C	G G	T T	G G	T T	G G
116	C C	C T	T T	T T	C C	G G	G T	A A	G G	T T	G G	T T	G G
117	C C	T T	T T	T T	A C	G G	G T	A A	A G	C T	A G	G T	C G
118	C C	C C	T T	T T	C C	A G	G G	A C	A G	C T	A G	G T	C G
119	C C	T T	T T	T T	A C	G G	G G	C C	G G	T T	G G	T T	G G
120	A C	C T	C T	T T	C C	A A	G G	A A	A G	C T	A G	G T	C G
121	C C	T T	T T	T T	A C	G G	G T	A C	A G	T T	A G	G T	C G
122	C C	C T	T T	T T	A C	G G	G T	A C	G G	T T	G G	T T	G G
123	A C	T T	C T	G T	A C	A G	G T	A A	A G	C T	A G	G T	C G
124	A A	T T	0 0	G G	A A	G G	G T	A C	A G	C T	A G	G T	C G
125	C C	0 0	T T	T T	A C	A A	0 0	0 0	0 0	T T	A A	0 0	0 0
126	A C	T T	0 0	G T	A A	G G	G G	C C	A A	C T	A A	G G	C C
127	C C	T T	T T	T T	A C	A G	G G	A C	A G	C T	A G	G T	C G
128	A C	C T	C T	T T	C C	A G	G G	A C	G G	T T	G G	T T	G G
129	A C	T T	0 0	0 0	A A	0 0	0 0	A C	0 0	C T	G G	0 0	0 0

130	A C	C T	C T	G T	A C	G G	T T	A A	A G	T T	A G	G T	C G
131	C C	C T	T T	T T	A C	0 0	0 0	A A	A A	C T	A A	G G	C C
132	A C	T T	C T	G T	A C	G G	T T	A A	A G	C T	A G	G T	C G
133	A C	T T	0 0	0 0	A A	G G	G T	A C	A A	T T	A A	G G	C C
134	A C	C T	C T	G T	A C	A G	G G	A A	A G	T T	A G	G T	C G
135	A C	T T	C T	T T	C C	A A	G G	A C	G G	T T	0 0	0 0	0 0
136	A C	T T	C T	G T	A A	A G	G G	A C	G G	T T	G G	T T	G G
137	C C	C T	T T	T T	A C	A G	G T	A A	A G	C T	A G	G T	C G
138	C C	T T	T T	T T	A C	G G	G T	A C	G G	T T	A G	G T	C G
139	A C	T T	C T	G T	A A	A G	G G	A C	A G	T T	A G	G T	C G
140	C C	T T	T T	T T	C C	A G	G T	A A	G G	T T	G G	T T	G G
141	A A	T T	0 0	G G	A A	G G	T T	A A	A G	T T	A G	G T	C G
142	A C	C T	C T	G T	A C	G G	G T	A A	G G	T T	A G	G T	G G
143	C C	T T	T T	T T	C C	A G	G G	A C	A G	C T	A G	G T	C G
144	C C	T T	T T	T T	A C	A G	G G	A A	A G	T T	A G	G T	C G
145	C C	T T	T T	T T	A C	A G	G T	A A	G G	T T	G G	T T	G G
146	C C	T T	T T	T T	C C	A G	G G	A A	A G	T T	A G	G T	C G
147	A C	C T	C T	G T	A C	G G	G G	A C	G G	T T	A G	G T	G G
148	C C	C T	T T	T T	A C	G G	G T	A A	A G	T T	A G	G T	C G
149	A C	T T	C T	G T	A A	G G	T T	A A	A G	T T	A G	G T	C G
150	A C	C T	C T	G T	A C	A A	0 0	A C	A G	T T	G G	0 0	0 0
151	C C	T T	T T	T T	A C	A G	G T	A A	A G	T T	A G	G T	C G
152	0 0	C T	T T	T T	C C	A G	G G	A A	G G	T T	G G	T T	G G
153	C C	C C	T T	T T	C C	G G	T T	A A	A G	C T	A G	G T	C G
154	A C	T T	0 0	0 0	0 0	G G	T T	A A	G G	T T	G G	T T	G G
155	A C	C T	C T	G T	A C	G G	G T	A C	G G	T T	G G	T T	G G
156	C C	C T	T T	T T	C C	G G	G T	A A	A G	T T	A G	G T	C G
157	A C	T T	C T	G T	A A	G G	G T	A A	A A	C T	A A	G G	C C
158	A C	C T	0 0	0 0	A C	0 0	G G	A C	G G	T T	0 0	0 0	0 0
159	C C	C C	T T	T T	C C	A G	G G	A A	G G	T T	G G	T T	G G
160	A C	0 0	0 0	0 0	A C	0 0	0 0	0 0	0 0	0 0	0 0	0 0	0 0
161	A A	T T	C C	G G	A A	A G	G T	A A	A G	T T	A G	G T	C G
162	C C	T T	C T	G T	A C	A G	G T	A C	A G	C T	A G	G T	C G

163	C C	T T	T T	T T	A A	G G	T T	A A	G G	T T	G G	T T	G G
164	A C	T T	C T	G T	A A	A G	G T	A A	G G	T T	G G	T T	G G
165	C C	C T	T T	T T	A C	G G	G T	A C	G G	T T	A G	G T	G G
166	C C	C T	T T	T T	C C	A G	G G	A C	A G	C T	A G	G T	C G
167	C C	C T	T T	T T	C C	G G	G T	A A	G G	T T	G G	T T	G G
168	A C	T T	C T	G T	A C	G G	T T	A A	A G	T T	A G	G T	C G
169	C C	T T	T T	T T	C C	G G	G T	A C	G G	T T	G G	T T	G G
170	A C	T T	C T	G T	A A	G G	T T	A A	G G	T T	G G	T T	G G
171	A C	C T	C T	G T	A C	G G	T T	A A	G G	T T	G G	T T	G G
172	0 0	T T	0 0	0 0	0 0	G G	0 0	A A	A A	T T	A A	G G	C C
173	A C	T T	C T	G T	A A	A G	G T	A A	A G	T T	A G	G T	C G
174	A A	T T	C C	G G	A A	G G	G T	A A	A G	T T	A G	G T	C G
175	C C	C C	T T	T T	C C	A G	G T	A A	G G	T T	G G	T T	G G
176	C C	C C	T T	T T	C C	A G	G T	A A	G G	T T	G G	T T	G G
177	A C	T T	0 0	0 0	A C	0 0	0 0	A C	G G	T T	0 0	0 0	G G
178	C C	T T	0 0	0 0	A C	0 0	0 0	A C	A G	T T	0 0	0 0	C G
179	A A	T T	0 0	0 0	A A	0 0	0 0	A A	G G	T T	0 0	0 0	C G
180	C C	C T	0 0	0 0	C C	0 0	0 0	A A	G G	T T	0 0	0 0	G G
181	A C	T T	0 0	0 0	A C	0 0	0 0	C C	A G	C T	0 0	0 0	C G
182	C C	C T	0 0	0 0	A C	0 0	0 0	A A	A A	T T	0 0	0 0	C C
183	A C	T T	0 0	0 0	A C	0 0	0 0	A A	G G	T T	0 0	0 0	G G
184	A C	T T	0 0	0 0	A A	0 0	0 0	A A	G G	C T	0 0	0 0	C G
185	A A	T T	0 0	0 0	A C	0 0	0 0	A A	G G	T T	0 0	0 0	G G
186	A C	T T	0 0	0 0	A A	0 0	0 0	A A	A G	T T	0 0	0 0	C G
187	C C	T T	0 0	0 0	A C	0 0	0 0	A A	A G	T T	0 0	0 0	C G
188	C C	T T	0 0	0 0	A C	0 0	0 0	A C	0 0	T T	0 0	0 0	C G
189	C C	0 0	0 0	0 0	A C	0 0	0 0	A C	G G	T T	0 0	0 0	G G
190	A C	T T	0 0	0 0	A C	0 0	G G	C C	A G	C T	0 0	0 0	C G
191	A C	T T	0 0	0 0	A C	0 0	0 0	A A	G G	T T	0 0	0 0	G G
192	A C	T T	0 0	0 0	A C	0 0	0 0	A C	G G	T T	0 0	0 0	G G
193	A C	T T	0 0	0 0	A A	0 0	T T	A A	A A	T T	0 0	0 0	C C
194	C C	C T	0 0	0 0	C C	0 0	0 0	A A	A G	T T	0 0	0 0	C G
195	C C	T T	0 0	0 0	C C	0 0	0 0	A A	G G	T T	0 0	0 0	G G

196	A C	T T	0 0	0 0	A C	0 0	0 0	A C	G G	T T	0 0	0 0	G G
197	C C	C T	0 0	0 0	A C	0 0	G G	A C	G G	T T	0 0	0 0	G G
198	C C	C C	0 0	0 0	C C	0 0	0 0	A C	A G	T T	0 0	0 0	C G
199	C C	C C	0 0	0 0	C C	0 0	G G	A A	G G	T T	0 0	0 0	G G
200	C C	T T	0 0	0 0	A C	0 0	G T	A C	G G	T T	0 0	0 0	G G
201	A C	0 0	0 0	0 0	C C	0 0	0 0	A A	G G	T T	0 0	0 0	G G
202	A C	T T	0 0	0 0	A C	0 0	G G	A C	G G	T T	0 0	0 0	G G
203	C C	C T	0 0	0 0	C C	0 0	0 0	A C	0 0	T T	0 0	0 0	G G
204	C C	C T	0 0	0 0	C C	0 0	G G	A A	G G	T T	0 0	0 0	G G
205	C C	C T	0 0	0 0	A C	0 0	T T	A A	G G	T T	0 0	0 0	G G
206	A C	T T	0 0	0 0	A A	0 0	0 0	A C	A G	C T	0 0	0 0	C G
207	A C	T T	0 0	0 0	A C	0 0	0 0	A A	G G	T T	0 0	0 0	G G
208	C C	T T	0 0	0 0	A C	0 0	0 0	A C	A A	C C	0 0	0 0	C C
209	C C	T T	0 0	0 0	A C	0 0	0 0	A C	A A	C C	0 0	0 0	C C
210	C C	C T	0 0	0 0	C C	0 0	T T	A A	0 0	T T	0 0	0 0	G G
211	A C	T T	0 0	0 0	A C	0 0	0 0	A C	G G	T T	0 0	0 0	G G
212	A C	T T	0 0	0 0	A A	0 0	0 0	A A	A G	T T	0 0	0 0	C G
213	A C	T T	0 0	0 0	A C	0 0	G G	A A	G G	T T	0 0	0 0	G G
214	C C	T T	0 0	0 0	C C	0 0	0 0	A A	A G	T T	0 0	0 0	C G
215	A A	0 0	0 0	0 0	A C	0 0	G T	A A	G G	T T	0 0	0 0	G G
216	C C	C T	0 0	0 0	C C	0 0	0 0	A C	G G	T T	0 0	0 0	G G
217	A A	T T	0 0	0 0	A A	0 0	T T	A A	A G	T T	0 0	0 0	C G
218	C C	C C	0 0	0 0	C C	0 0	0 0	A C	A G	T T	0 0	0 0	C G
219	C C	C T	0 0	0 0	C C	0 0	G G	C C	A G	C T	0 0	0 0	C G
220	A A	T T	0 0	0 0	A A	0 0	G T	A A	G G	T T	0 0	0 0	G G
221	C C	C T	0 0	0 0	C C	0 0	G T	A A	G G	T T	0 0	0 0	G G
222	C C	C T	0 0	0 0	A C	0 0	T T	A A	G G	T T	0 0	0 0	G G
223	C C	T T	0 0	0 0	A A	0 0	G T	A A	A A	C C	0 0	0 0	C C
224	A C	C T	0 0	0 0	A C	0 0	0 0	A C	A G	T T	0 0	0 0	C G
225	A C	T T	0 0	0 0	A C	0 0	G G	A C	G G	T T	0 0	0 0	G G
226	A C	0 0	0 0	0 0	A A	0 0	G G	A C	G G	T T	0 0	0 0	G G
227	A C	C T	0 0	0 0	A C	0 0	G G	C C	0 0	T T	0 0	0 0	G G
228	A A	T T	0 0	0 0	A A	0 0	G G	A C	0 0	T T	0 0	0 0	G G

229	C C	C T	0 0	0 0	C C	0 0	T T	A A	A G	T T	0 0	0 0	C G
230	A A	T T	0 0	0 0	A C	0 0	0 0	A C	A G	T T	0 0	0 0	G G
231	C C	T T	0 0	0 0	C C	0 0	T T	A A	G G	T T	0 0	0 0	G G
232	A C	T T	0 0	0 0	A C	0 0	0 0	A A	0 0	T T	0 0	0 0	G G
233	C C	C C	0 0	0 0	C C	0 0	G T	A C	0 0	T T	0 0	0 0	G G
234	C C	T T	0 0	0 0	A A	0 0	G G	A C	A G	T T	0 0	0 0	C G
235	C C	T T	0 0	0 0	A C	0 0	G T	A A	G G	T T	0 0	0 0	G G
236	C C	C C	0 0	0 0	C C	0 0	G T	A C	0 0	T T	0 0	0 0	G G
237	A C	0 0	0 0	0 0	A C	0 0	G T	A A	G G	T T	0 0	0 0	G G
238	C C	T T	0 0	0 0	A C	0 0	G G	A A	A G	T T	0 0	0 0	C G
239	A A	T T	0 0	0 0	A A	0 0	G T	A C	G G	T T	0 0	0 0	G G
240	A C	C T	0 0	0 0	A C	0 0	G T	A C	G G	T T	0 0	0 0	G G
241	C C	T T	0 0	0 0	C C	0 0	T T	A A	G G	T T	0 0	0 0	G G
242	A C	T T	0 0	0 0	A A	0 0	G T	A A	A G	C T	0 0	0 0	C G
243	C C	T T	0 0	0 0	C C	0 0	G G	A C	G G	T T	0 0	0 0	G G
244	C C	C C	0 0	0 0	C C	0 0	0 0	A C	G G	T T	0 0	0 0	G G
245	A A	T T	0 0	0 0	A A	0 0	G T	A A	A G	T T	0 0	0 0	C G
246	A C	C T	0 0	0 0	A C	0 0	G T	A A	G G	T T	0 0	0 0	G G
247	A A	T T	0 0	0 0	A A	0 0	G T	A A	G G	T T	0 0	0 0	G G
248	C C	C T	0 0	0 0	A C	0 0	T T	A A	A G	C T	0 0	0 0	C G
249	A C	0 0	0 0	0 0	A A	0 0	G G	A C	A G	T T	0 0	0 0	G G
250	A C	T T	0 0	0 0	A C	0 0	G G	C C	A G	T T	0 0	0 0	C G
251	A C	T T	0 0	0 0	A A	0 0	G T	A A	G G	T T	0 0	0 0	G G
252	A C	T T	0 0	0 0	C C	0 0	G G	A A	0 0	T T	0 0	0 0	G G
253	A C	C T	0 0	0 0	A C	0 0	T T	A A	A G	T T	0 0	0 0	C G
254	C C	T T	0 0	0 0	A A	0 0	0 0	A A	A G	C T	0 0	0 0	C G
255	C C	C T	0 0	0 0	C C	0 0	T T	A A	A G	T T	0 0	0 0	C G
256	C C	T T	0 0	0 0	A C	0 0	G G	A A	G G	T T	0 0	0 0	G G
257	C C	C C	0 0	0 0	C C	0 0	G G	A A	G G	T T	0 0	0 0	G G
258	C C	T T	0 0	0 0	A C	0 0	T T	A A	A G	C T	0 0	0 0	C G
259	C C	C T	0 0	0 0	C C	0 0	G G	A C	A G	T T	0 0	0 0	C G
260	A C	T T	0 0	0 0	A C	0 0	G G	A C	A G	T T	0 0	0 0	C G
261	0 0	0 0	0 0	0 0	0 0	0 0	0 0	A C	G G	T T	0 0	0 0	G G

262	C C	C C	0 0	0 0	C C	0 0	G T	A C	A A	C T	0 0	0 0	C C
263	A C	T T	0 0	0 0	C C	0 0	G G	A C	G G	T T	0 0	0 0	G G
264	A C	T T	0 0	0 0	A A	0 0	G T	A A	G G	T T	0 0	0 0	G G
265	C C	C T	T T	0 0	C C	G G	G T	A C	G G	T T	G G	T T	G G
266	A A	T T	C C	0 0	A A	A G	G G	A C	G G	T T	G G	T T	G G
267	C C	C T	T T	0 0	C C	G G	G T	A A	A G	T T	A G	G T	C G
268	A C	T T	C T	0 0	A C	G G	G G	A A	G G	T T	G G	T T	G G
269	C C	C T	T T	0 0	C C	G G	G T	A C	A G	C T	A G	G T	C G
270	C C	T T	T T	0 0	A C	A G	G G	A A	A G	T T	A G	G T	C G
271	A C	C T	C T	0 0	A C	A G	G T	A A	A G	C T	A A	G G	C C
272	C C	C C	T T	0 0	C C	G G	G G	A C	A G	C T	A A	G G	C G
273	C C	T T	T T	0 0	C C	A G	G T	A A	G G	T T	G G	T T	G G
274	A A	T T	C C	0 0	A A	G G	G G	A C	A G	C T	A A	G G	C G
275	A C	C T	0 0	0 0	C C	A G	G G	A C	A G	T T	A G	G T	C G
276	0 0	T T	0 0	0 0	0 0	G G	G T	A C	G G	T T	G G	T T	G G
277	C C	T T	T T	0 0	A A	A G	G T	A A	G G	T T	G G	T T	G G
278	A C	T T	C T	0 0	A C	G G	T T	A A	G G	T T	G G	T T	G G
279	C C	T T	T T	0 0	A A	A G	G T	A A	G G	T T	G G	T T	G G
280	C C	T T	T T	0 0	C C	G G	G G	C C	A G	T T	A G	G T	C G
281	C C	C T	T T	0 0	C C	G G	G T	A C	A G	T T	A G	G T	C G
282	A C	C T	C T	0 0	A C	G G	G T	A A	G G	T T	G G	T T	G G
283	A C	T T	C T	0 0	C C	G G	G G	C C	G G	T T	G G	T T	G G
284	A C	C T	C T	0 0	A C	G G	G T	A A	G G	T T	G G	T T	G G

GENE	SIRT1	BDNF	BDNF	BDNF	LINC01559	LINC01559	LINC01559	LINC01559	GRIN2B	GRIN2B	GRIN2B	GRIN2B	GRIN2B
SNP/Sample	rs10997875	rs7124442	rs6265	rs11030121	rs1457624	rs17821405	rs12423809	rs10845757	rs10845763	rs10744030	rs12814951	rs1806191	rs1806201
1	C T	T T	A A	C C	A C	G G	A A	C T	A C	A G	G T	A G	G G
2	C C	C T	G G	C T	A C	G G	A A	T T	A C	A G	T T	A A	G G
3	T T	C T	G G	C T	C C	G T	A C	C C	A C	G G	G T	A G	G G
4	C C	T T	G G	C C	A A	G G	A A	T T	A A	G G	G T	A A	G G
5	C C	T T	A A	C C	A C	G T	A C	C C	A A	G G	G T	A G	A G
6	C T	T T	G G	C C	A C	G T	A C	C C	A C	G G	T T	A A	G G
7	0 0	T T	G G	C C	A C	G G	A A	T T	A A	A A	G T	A G	A G
8	0 0	T T	A G	C C	A C	G T	C C	C C	C C	A A	0 0	A G	A G
9	C C	C C	G G	T T	A A	G G	A A	C T	A C	A G	G G	A G	G G
10	C T	T T	A G	C C	C C	G T	A C	C C	A C	G G	G T	A G	G G
11	C T	T T	G G	C C	A A	G G	A A	C C	A C	A G	G G	G G	A G
12	C C	T T	A G	C C	C C	G T	A C	C T	A C	G G	T T	A A	G G
13	C C	T T	G G	C C	C C	T T	A C	C C	A A	A G	T T	A A	G G
14	C C	C C	G G	T T	C C	T T	C C	C C	C C	A A	G T	A G	G G
15	C T	T T	G G	C T	A C	G T	A C	C T	A C	A G	G T	A G	A G
16	C T	T T	A A	C C	A C	G T	A A	C T	A C	A G	G G	G G	G G
17	C T	T T	G G	C C	A C	G T	A C	C T	C C	G G	G G	G G	G G
18	C C	C T	G G	C T	A C	G T	C C	C C	C C	G G	G T	A G	G G
19	0 0	C T	G G	C T	C C	T T	C C	C C	A C	A G	T T	A G	G G
20	0 0	C C	G G	T T	A A	G G	A A	T T	C C	G G	G T	A G	G G
21	C T	T T	G G	C C	C C	G T	A C	C T	A C	G G	T T	A A	G G
22	C T	T T	A G	C C	A C	G G	A C	C T	C C	A A	G T	A A	G G
23	C T	C T	A G	C T	A A	G G	A A	T T	A C	G G	T T	A A	G G
24	C C	C T	G G	C T	C C	G T	A C	C T	A C	A G	T T	A A	G G
25	C C	T T	A G	C C	C C	G G	A A	T T	A A	G G	G T	A G	A G
26	C C	C T	G G	C T	C C	G T	A C	C C	A A	A G	T T	A A	G G
27	C C	T T	G G	C C	A A	G G	A A	C T	A C	G G	G G	G G	A G
28	C C	T T	G G	C C	A A	G G	A C	C T	A C	G G	T T	A A	G G
29	C C	T T	A G	C C	A A	G G	A A	C T	A C	A G	T T	A A	G G
30	C C	T T	G G	C C	C C	T T	A C	C C	A A	A G	G T	A G	A G

31	0 0	T T	A G	C C	A A	G G	A A	C T	C C	A G	T T	A G	G G
32	0 0	C C	G G	T T	C C	G T	A C	C T	A C	A G	G G	G G	A A
33	C C	C T	A G	C T	C C	G T	A C	C T	A C	G G	T T	A A	G G
34	C C	T T	A G	C C	A A	G G	A A	T T	A C	G G	T T	A A	G G
35	C C	C T	G G	C T	A C	G T	A C	C C	C C	A A	G G	G G	A G
36	C T	T T	A G	C C	A A	G G	A A	T T	A A	A G	G T	G G	A G
37	0 0	C C	G G	T T	A A	G G	A A	C T	A C	A G	0 0	A A	G G
38	C T	T T	G G	C C	A A	G G	A A	C T	A A	G G	G T	A A	G G
39	0 0	T T	G G	C T	A C	G T	A C	C T	C C	A A	0 0	A A	G G
40	C C	T T	A G	C C	A C	G T	A C	C C	A C	A A	G G	G G	A A
41	0 0	T T	A G	C C	A C	G T	A C	T T	A A	A G	0 0	A G	A G
42	C T	C T	G G	C T	C C	G T	A C	C T	A A	G G	G T	A G	A G
43	0 0	C T	G G	C C	C C	G T	A C	C C	C C	G G	0 0	A G	A G
44	0 0	T T	A G	C C	C C	G T	C C	C C	C C	A G	G G	G G	G G
45	T T	C T	G G	C T	A A	G G	A A	T T	C C	G G	0 0	A G	A G
46	C T	C C	G G	T T	C C	G T	A C	C T	A C	G G	G T	G G	G G
47	C C	T T	G G	C C	A A	G G	A C	C T	A A	G G	0 0	A A	G G
48	C C	C C	G G	T T	C C	T T	A C	C T	C C	G G	T T	A G	G G
49	C T	C T	A G	C T	A A	G G	A A	C T	A A	G G	G T	A G	A G
50	0 0	C T	G G	C T	A C	G T	A C	C T	A C	A G	0 0	A A	G G
51	C C	T T	G G	C C	C C	G T	C C	C C	A C	G G	T T	A A	G G
52	0 0	T T	A G	C C	A C	G G	A A	T T	C C	A G	0 0	G G	A G
53	C C	C T	A G	C T	A C	G T	A C	C T	A A	A G	G T	A G	A G
54	C T	T T	G G	C C	A C	G G	A C	C C	A C	A G	0 0	G G	G G
55	0 0	T T	G G	C C	C C	T T	C C	C C	C C	A G	G T	A G	G G
56	0 0	T T	G G	C C	A A	G G	A A	C C	A C	G G	0 0	G G	A G
57	C T	C T	G G	C T	A C	G G	A C	C T	A C	A G	G T	A A	G G
58	C C	C T	G G	0 0	A A	G G	A A	T T	A C	A A	G T	A G	A G
59	C T	T T	G G	C C	A A	G G	A A	T T	A A	A G	T T	A A	G G
60	C C	T T	G G	C C	A C	G T	A C	C T	A C	G G	G G	G G	A G
61	C T	T T	A G	C C	C C	T T	A C	C C	C C	A G	G G	A A	G G
62	C C	C T	G G	C T	A C	G G	A A	T T	C C	G G	0 0	A G	A G
63	C C	C T	A G	C T	A C	G G	C C	C C	C C	G G	G T	A A	G G

64	C C	T T	G G	C C	C C	0 0	C C	C T	A C	G G	G T	A G	G G
65	C T	T T	A G	C C	A A	G G	A A	T T	C C	A G	T T	A A	G G
66	C T	T T	G G	C C	0 0	T T	A A	0 0	A A	A A	G G	G G	A A
67	0 0	C T	A G	C T	A C	G T	A C	C T	A C	A A	G G	A G	A G
68	0 0	T T	G G	C C	A C	G G	A C	C T	A A	G G	G T	A G	A G
69	C C	T T	A G	C C	A C	G G	C C	C C	A C	A G	G T	A A	G G
70	C C	C T	G G	C T	A C	G T	A C	C T	A C	A G	G T	A A	G G
71	C C	T T	G G	C C	A C	G G	A A	T T	C C	G G	G G	G G	A G
72	C T	T T	A G	C C	A C	G T	A C	C T	A C	A A	G T	A G	A G
73	C C	T T	A A	C C	A C	G T	C C	C C	C C	G G	G G	G G	G G
74	C T	T T	G G	C C	A C	G T	A A	C T	A C	A G	T T	A A	G G
75	C C	C C	G G	T T	C C	G T	C C	C C	A C	G G	T T	A A	G G
76	C C	C T	G G	C T	A C	G G	A A	C C	C C	A G	T T	A A	G G
77	C T	C C	G G	T T	C C	T T	C C	C C	C C	A A	G T	A A	G G
78	T T	T T	G G	C C	A C	G T	A C	C C	A A	G G	G T	A G	G G
79	0 0	C T	A G	C T	A C	G T	A C	C T	A A	G G	G T	A A	G G
80	0 0	C T	G G	C T	A C	G G	A C	C T	C C	A G	G T	A G	A G
81	C C	C T	G G	C T	A A	G G	A C	C T	A C	A G	G G	A G	A G
82	C C	T T	G G	C C	A C	G G	A A	T T	A C	A G	G T	A G	A G
83	C C	T T	G G	C C	A C	G T	A C	C T	C C	A G	G T	A G	G G
84	C T	T T	G G	C C	A C	G T	A C	C T	A C	A G	G T	G G	A G
85	C C	C T	G G	C T	C C	G G	A C	C T	C C	A A	T T	A A	G G
86	C C	C T	G G	C T	A C	G G	A A	C T	A A	G G	G T	A G	G G
87	0 0	C T	A G	C T	A C	G T	A C	C T	C C	G G	G T	A G	G G
88	CC	TT	AG	CC	AA	GG	AA	CC	AC	GG	GT	AA	GG
89	C C	C C	G G	0 0	0 0	0 0	0 0	0 0	0 0	G G	0 0	0 0	0 0
90	C T	C T	G G	C T	C C	G G	A A	T T	C C	G G	G G	G G	G G
91	C T	T T	G G	C C	C C	G T	C C	C C	A C	A G	G T	A G	A G
92	C C	C T	G G	C T	A A	G G	A A	C T	A A	G G	G T	A A	G G
93	C C	T T	A G	C C	A C	G T	A A	C T	A C	A G	G G	G G	A A
94	T T	C T	G G	C T	C C	T T	A C	C T	A A	G G	G T	A A	G G
95	C C	C T	G G	C C	C C	G G	A A	C T	A A	A G	G T	A G	G G
96	C T	C C	G G	T T	C C	G T	C C	C C	A C	A G	G G	G G	A G

97	C C	C T	G G	C T	A C	G T	A C	C C	A C	A A	G T	A G	G G
98	T T	T T	G G	C C	C C	G G	A C	C T	A C	A G	T T	G G	G G
99	C T	T T	A A	C C	A A	G G	A A	T T	A C	G G	T T	A G	G G
100	0 0	T T	G G	C T	A A	G G	A A	C T	C C	G G	0 0	A G	G G
101	C T	T T	A G	C C	A C	G T	A C	C T	A C	A G	T T	A G	G G
102	C T	T T	G G	C C	A A	G G	A A	C C	A A	A G	G G	G G	A A
103	T T	T T	G G	C C	C C	G T	C C	C C	C C	G G	G G	G G	A A
104	C C	T T	G G	C C	C C	T T	C C	C C	C C	A G	G T	A G	G G
105	T T	T T	A A	C C	A C	G G	A A	C T	A C	G G	T T	A A	G G
106	C C	C T	G G	C T	A A	G G	A A	C T	A C	A G	T T	A A	G G
107	C T	C T	G G	C T	C C	G G	A A	C T	A A	A G	G T	A G	G G
108	C C	C C	G G	T T	C C	T T	C C	C C	A C	G G	G G	A G	A G
109	C T	C T	A G	C T	A A	G G	A A	T T	A C	A A	T T	A A	G G
110	C C	C T	G G	C T	C C	T T	C C	C C	A C	G G	G T	A G	A G
111	C C	T T	A G	C C	C C	G G	C C	C C	A C	G G	G T	G G	A G
112	C T	C C	G G	0 0	A C	G G	A A	T T	C C	A A	G T	A G	A G
113	C C	C T	A G	C T	A C	G T	A A	T T	C C	A G	G T	A G	G G
114	C C	C T	G G	C C	C C	G T	A C	C T	A C	A G	G T	A A	G G
115	C C	C T	G G	C T	A A	G G	A C	C T	A A	G G	G T	A G	A G
116	C C	T T	G G	C C	A C	G T	A C	C T	A A	A G	G T	A G	A G
117	C T	T T	G G	C C	A A	G G	A A	T T	A C	G G	G T	A G	A G
118	C T	C T	G G	C T	A C	G T	A C	C T	A A	G G	G T	A G	A G
119	C C	C T	A G	C T	A C	G G	A A	C T	C C	A A	G T	A G	A G
120	C T	C T	A G	C T	C C	G G	C C	C C	A C	A G	G G	G G	A G
121	C T	C T	G G	C T	A C	G T	A A	C T	C C	G G	T T	A A	G G
122	C C	C T	G G	C T	A C	G T	A C	C C	C C	A G	T T	A A	G G
123	C T	C T	A G	C T	A C	G T	A C	C T	A C	A G	T T	A A	G G
124	C T	C T	G G	C T	C C	G G	A A	C T	C C	G G	G G	G G	G G
125	C T	C C	G G	T T	A A	G G	A C	C T	A A	A G	G G	A G	A G
126	T T	T T	G G	C C	C C	T T	A C	C C	A C	A G	G G	G G	A G
127	C T	T T	G G	C C	C C	T T	A A	C C	A C	G G	G G	A G	G G
128	C C	T T	G G	C C	A C	G T	A C	C T	C C	A G	G T	A G	A G
129	C T	T T	G G	C C	C C	0 0	A A	0 0	A C	G G	G T	G G	A G

130	C T	T T	G G	C C	A A	G G	A A	T T	A C	G G	G T	A G	A G
131	T T	T T	G G	C C	C C	T T	A C	C C	A C	A G	G G	G G	A A
132	C T	T T	A G	C C	C C	G T	A C	C T	A C	A G	T T	A A	G G
133	T T	0 0	0 0	0 0	C C	0 0	C C	0 0	A A	0 0	T T	0 0	G G
134	C T	T T	A G	C C	C C	G T	A C	C C	A C	G G	T T	A A	G G
135	C C	T T	A G	C C	A C	G T	A C	C T	C C	A G	T T	A G	G G
136	C C	T T	G G	C C	A A	G G	A C	C T	A C	G G	G T	A G	A G
137	C T	C T	A G	C T	A C	G G	A C	C T	C C	G G	G G	A G	G G
138	C T	C T	A G	C T	A C	G T	A C	C T	A C	A G	G T	A A	G G
139	C T	T T	A G	C C	A C	G T	A C	C C	A A	A G	T T	A A	G G
140	C C	C C	G G	T T	A C	G T	A C	C C	A C	G G	G G	A G	G G
141	C T	C C	G G	T T	C C	G T	A C	C T	C C	G G	T T	A G	G G
142	C C	T T	A G	C C	C C	T T	C C	C C	A A	G G	T T	A A	G G
143	C T	C C	G G	T T	A C	G T	A C	C C	A A	A G	G G	G G	A G
144	C T	T T	A A	C C	A C	G T	A C	C T	A C	A G	G G	A A	G G
145	C C	T T	A G	C C	C C	G T	A C	C T	C C	A G	G G	G G	A A
146	C T	C T	G G	C T	C C	G T	C C	C C	C C	G G	G T	A G	G G
147	C C	0 0	0 0	0 0	C C	G G	C C	C C	C C	0 0	G T	0 0	G G
148	C T	C T	A G	C T	A C	G T	C C	C C	A C	A G	G T	A G	A G
149	C T	C T	G G	C T	C C	T T	A C	C C	A C	A G	G T	A G	G G
150	C T	C T	G G	C C	C C	T T	A C	C C	A C	A G	G G	G G	A G
151	C T	T T	A G	C C	A C	G T	A A	C C	A C	G G	G T	A A	G G
152	C C	T T	G G	C C	A C	G T	A C	C T	A C	A A	G T	A G	G G
153	C T	C T	G G	C T	A A	G G	A A	C T	A A	G G	G G	A A	G G
154	C C	C T	G G	C T	0 0	T T	A A	0 0	C C	A G	G T	A A	G G
155	C C	C T	G G	C T	A C	G G	A A	T T	A C	G G	T T	A A	G G
156	C T	C T	A G	C T	A C	G T	A C	C T	A C	A G	G G	A G	A G
157	T T	T T	G G	C C	C C	G T	C C	C C	A C	A A	T T	A A	G G
158	C C	C T	G G	C T	C C	0 0	C C	C T	A A	A G	G G	A G	A G
159	C C	C T	A G	C T	A C	G T	A C	C T	A C	G G	G T	A A	G G
160	0 0	C T	G G	C T	0 0	0 0	A C	0 0	A C	G G	0 0	A G	A G
161	C T	T T	G G	C C	A C	G T	A C	C T	A C	A G	T T	A A	G G
162	C T	C T	G G	C T	A C	G G	A A	C T	C C	A G	G G	G G	A G

163	C C	C T	G G	C T	C C	G G	A A	T T	A C	A G	G G	A G	A G
164	C C	T T	A G	C C	A C	G G	A C	C T	A C	G G	G T	A A	G G
165	C C	T T	G G	C C	A A	G G	A A	T T	A A	G G	G T	A A	G G
166	C T	C T	G G	C T	C C	T T	A C	C C	A C	G G	G T	A G	A G
167	C C	T T	G G	C C	A C	G T	A A	C T	A C	A G	G G	A A	G G
168	C T	C T	G G	C T	A A	G G	A A	C T	C C	A A	G T	A A	G G
169	C C	T T	A G	C C	A C	G T	A C	C T	A A	A G	G T	A G	A G
170	C C	C C	G G	T T	C C	G T	A C	C T	C C	A G	G T	A G	G G
171	C C	T T	A G	C C	A C	G T	A C	C T	A C	A G	G G	G G	A A
172	T T	T T	A A	C C	0 0	0 0	0 0	0 0	0 0	A A	0 0	A G	0 0
173	C T	C T	G G	C T	A A	G G	A A	C T	A C	G G	G T	G G	A G
174	C T	T T	G G	C C	A A	G G	A A	T T	A A	G G	T T	A A	G G
175	C C	C T	G G	C T	A C	G G	A A	C T	A A	A G	T T	A A	G G
176	C C	T T	G G	C C	A C	G T	A C	C C	A A	G G	G T	A G	G G
177	C T	C T	G G	0 0	A C	0 0	A C	C T	A C	A G	G G	G G	A G
178	C T	T T	A G	0 0	C C	0 0	A C	C C	C C	A G	G G	G G	A G
179	C T	T T	G G	0 0	A A	0 0	A A	T T	A C	G G	T T	A A	G G
180	C C	T T	A G	0 0	A C	0 0	A C	C T	A A	G G	G T	A G	G G
181	C T	T T	G G	0 0	A A	0 0	A A	C T	A C	A G	T T	A G	G G
182	T T	C T	G G	0 0	A A	0 0	A A	C T	A C	G G	G G	A G	A G
183	C C	T T	A A	0 0	A A	0 0	A C	C T	A C	G G	G G	G G	G G
184	C T	T T	A G	0 0	A C	0 0	A C	C C	A A	G G	G G	A G	G G
185	C C	0 0	0 0	0 0	A C	0 0	A A	C T	C C	0 0	G G	0 0	G G
186	C T	C T	A G	0 0	A A	0 0	A A	T T	A C	A A	G G	A G	G G
187	C T	C T	G G	0 0	A A	0 0	A C	C C	A A	G G	G G	A A	G G
188	C T	T T	A G	0 0	A C	0 0	A C	C C	A A	G G	G G	A G	A G
189	C T	T T	A G	0 0	C C	0 0	A C	C T	A C	A G	G G	A G	A G
190	C T	T T	A G	0 0	C C	0 0	A C	C C	A C	A G	G T	A A	G G
191	C C	C T	G G	0 0	A C	0 0	A A	T T	A C	G G	G T	A A	G G
192	C C	T T	G G	0 0	A A	0 0	A A	T T	A C	A G	G G	A A	G G
193	T T	T T	G G	0 0	A C	0 0	A C	C C	A A	G G	G G	A G	A G
194	C T	C T	A G	0 0	C C	0 0	C C	C C	A C	A G	G G	G G	G G
195	C C	C T	G G	0 0	A C	0 0	A C	C T	A A	G G	G T	A A	G G

196	C C	C T	A G	0 0	A C	0 0	A A	C T	C C	A A	G T	A A	G G
197	C C	T T	A G	0 0	A C	0 0	A C	C T	A A	G G	G G	G G	0 0
198	C T	C T	G G	0 0	A C	0 0	A A	C C	C C	A G	G G	A G	G G
199	C C	T T	G G	0 0	A C	0 0	A A	C T	A C	G G	G T	G G	G G
200	C C	C C	G G	0 0	C C	0 0	C C	C C	C C	A G	G G	A G	G G
201	C T	C T	G G	0 0	A C	0 0	A A	T T	A C	A G	G T	A G	G G
202	0 0	T T	A G	0 0	A C	0 0	A A	C T	C C	A G	G G	A G	G G
203	C C	T T	G G	0 0	A C	0 0	A C	C C	A C	A A	G G	G G	G G
204	C C	T T	G G	0 0	A A	0 0	A A	C C	A C	G G	G G	A G	A G
205	C C	C T	G G	0 0	A C	0 0	A C	C C	A C	A A	G T	0 0	G G
206	C T	C T	A G	0 0	A A	0 0	A A	T T	C C	A G	G G	A G	A G
207	C C	C C	G G	0 0	A C	0 0	A A	T T	C C	G G	G T	A G	G G
208	T T	T T	G G	0 0	C C	0 0	A C	C C	A C	G G	G G	G G	A G
209	T T	T T	G G	0 0	C C	0 0	A C	C C	A C	G G	G G	G G	A G
210	C C	T T	A G	0 0	C C	0 0	C C	C C	A C	A G	G G	G G	G G
211	C C	C T	G G	0 0	A C	0 0	A C	C C	C C	G G	G T	A G	G G
212	C T	T T	A G	0 0	C C	0 0	A C	C C	A C	G G	G G	A G	G G
213	C T	T T	A G	0 0	C C	0 0	C C	C C	C C	A G	G G	A G	A G
214	C T	T T	G G	0 0	A A	0 0	A A	T T	A C	A G	G T	A A	G G
215	0 0	C T	G G	0 0	C C	0 0	A C	C T	A C	A G	G G	G G	A A
216	C C	T T	G G	0 0	A C	0 0	C C	C C	A C	A G	G T	A A	G G
217	C T	C C	G G	0 0	C C	0 0	A C	C T	A C	G G	G G	G G	A A
218	C T	C T	G G	0 0	C C	0 0	C C	C C	C C	G G	G G	G G	G G
219	C T	C T	A G	0 0	C C	0 0	C C	C C	A C	A A	G T	A A	G G
220	C C	C T	A G	0 0	C C	0 0	C C	C C	A C	A G	G G	G G	A G
221	C C	T T	A A	0 0	C C	0 0	A C	C T	C C	G G	G G	A G	G G
222	C C	C T	A G	0 0	C C	0 0	C C	C C	A C	A G	G G	G G	G G
223	T T	T T	G G	0 0	A C	0 0	A A	C T	C C	A G	G G	A G	G G
224	C T	T T	G G	0 0	A C	0 0	A A	C T	0 0	A G	G G	G G	A G
225	C T	T T	G G	0 0	A C	0 0	A C	C T	A C	G G	G G	A G	G G
226	C T	T T	G G	0 0	A C	0 0	A A	T T	A C	A G	G G	G G	A G
227	C C	C T	G G	0 0	C C	0 0	C C	C C	C C	G G	G G	G G	A G
228	C C	T T	G G	0 0	A C	0 0	A A	C T	C C	A G	G T	A A	G G

229	T T	T T	A A	0 0	A A	0 0	A A	T T	A A	A G	T T	A G	G G
230	C C	C C	G G	0 0	A C	0 0	0 0	C T	A A	A G	G G	A G	A G
231	C C	T T	G G	0 0	A A	0 0	A A	C T	C C	A G	G G	G G	G G
232	C C	T T	G G	0 0	A C	0 0	A C	C C	A A	G G	G G	A G	G G
233	C C	T T	G G	0 0	A C	0 0	A C	C C	C C	A G	G G	G G	0 0
234	C T	C T	G G	0 0	A C	0 0	C C	C C	A A	A G	G G	A G	A G
235	C C	C C	G G	0 0	A C	0 0	A C	C T	A A	G G	G T	A G	G G
236	C C	C T	G G	0 0	C C	0 0	A A	T T	A A	A G	G G	A G	A G
237	C T	C T	G G	0 0	0 0	0 0	A C	T T	C C	A G	G G	A G	A G
238	C T	T T	A G	0 0	C C	0 0	C C	C C	A C	A G	G T	A G	G G
239	C C	T T	G G	0 0	A C	0 0	A C	C C	A A	A G	G G	A G	G G
240	C C	C T	G G	0 0	A A	0 0	A C	C C	A C	G G	G G	A G	A G
241	C C	T T	A G	0 0	A C	0 0	A C	C T	C C	A A	G G	A G	A G
242	C T	C T	G G	0 0	C C	0 0	C C	C C	A A	A A	G T	A A	G G
243	C C	C T	G G	0 0	A C	0 0	A C	C T	A C	G G	G G	A G	G G
244	C C	T T	A G	0 0	A C	0 0	A C	C C	A C	A G	G G	A G	G G
245	C T	T T	G G	0 0	C C	0 0	A C	C C	C C	G G	G G	G G	A G
246	C C	T T	G G	0 0	A A	0 0	A A	T T	A C	A A	G G	G G	0 0
247	C C	C T	G G	0 0	C C	0 0	C C	C C	C C	A A	G G	A G	G G
248	C T	T T	G G	0 0	A C	0 0	A A	C T	A A	A G	G G	G G	A A
249	C T	C T	G G	0 0	C C	0 0	C C	C C	A C	A G	G G	G G	A G
250	C T	T T	A G	0 0	A A	0 0	A A	C T	C C	G G	G G	A G	G G
251	C C	C T	G G	0 0	A A	0 0	A A	T T	C C	G G	G G	G G	G G
252	C C	C C	G G	0 0	C C	0 0	A C	C T	A C	A G	G G	A G	A G
253	C T	T T	A G	0 0	C C	0 0	A C	T T	A A	G G	G G	A G	G G
254	C T	C T	G G	0 0	C C	0 0	A C	C C	C C	A A	G G	A G	A G
255	C T	T T	G G	0 0	A C	0 0	A C	C T	C C	A A	G G	G G	G G
256	C C	T T	G G	0 0	C C	0 0	C C	C C	C C	A A	G G	A G	G G
257	C C	C T	G G	0 0	C C	0 0	A C	C T	A C	G G	G T	A A	G G
258	C T	C T	G G	0 0	A A	0 0	A A	T T	A C	A G	G G	A G	A G
259	C T	T T	A G	0 0	A C	0 0	A C	C C	A C	A G	G G	G G	A G
260	C T	C T	G G	0 0	A A	0 0	A A	T T	A A	A G	G T	A G	G G
261	C T	C T	G G	0 0	0 0	0 0	0 0	0 0	0 0	A G	G G	G G	0 0

262	T T	T T	A G	0 0	C C	0 0	A A	C T	A C	G G	G G	A G	A G
263	C C	C T	G G	0 0	C C	0 0	A C	C T	C C	A G	G G	A G	G G
264	C C	C T	A G	0 0	A C	0 0	A A	T T	C C	A A	G T	A G	G G
265	C C	T T	G G	C C	A A	0 0	A A	T T	A A	G G	G G	G G	A A
266	C C	C T	G G	C T	A A	0 0	A A	T T	A C	A G	T T	A A	G G
267	C T	T T	G G	C C	C C	0 0	C C	C C	C C	G G	G G	G G	A A
268	C C	T T	A G	C C	C C	0 0	C C	C C	A C	A G	G T	A G	A G
269	C T	C T	G G	C T	A A	0 0	A C	C C	C C	G G	G T	A G	G G
270	C T	C T	G G	C T	A C	0 0	A A	C T	A C	G G	G T	A G	G G
271	T T	C T	G G	C T	A C	0 0	A C	C C	C C	A G	G G	G G	A G
272	C T	T T	A G	C C	A A	0 0	A A	C T	A C	G G	G G	A G	A G
273	C C	T T	A A	C C	A A	0 0	A A	T T	A A	A G	G G	G G	A A
274	C T	T T	A G	C C	C C	0 0	A C	C T	C C	G G	G G	A G	G G
275	C T	C T	G G	C T	C C	0 0	C C	C C	A C	A G	T T	A A	G G
276	C C	T T	G G	C C	0 0	0 0	0 0	0 0	0 0	A G	G T	A G	0 0
277	C C	C T	G G	C C	A A	0 0	A A	C T	A C	G G	G G	A G	A G
278	C C	C C	G G	T T	A C	0 0	A C	C T	A C	A G	G G	G G	A A
279	C C	C T	A G	C T	A A	0 0	A A	C T	A C	A G	G T	G G	A G
280	C T	C T	G G	C T	C C	0 0	C C	C C	A C	A G	G T	A G	A G
281	C T	C T	G G	C T	C C	0 0	A C	C C	C C	A G	G T	A G	G G
282	C C	C T	A G	C T	A A	0 0	A A	T T	A A	G G	T T	A G	G G
283	C C	T T	A G	C C	A C	0 0	A C	C T	C C	G G	G T	A G	A G
284	C C	C T	A G	C T	A A	0 0	A C	C C	C C	G G	G T	A G	A G

GENE	GRIN2B	GRIN2B	GRIN2B	GRIN2B	GRIN2B	GRIN2B	GRIN2B	GRIN2B	GRIN2B	GRIN2B	GRIN2B	GRIN2B-ATF7IP	GRIN2B-ATF7IP
SNP/Sample	rs4764011	rs1805539	rs2300235	rs2284406	rs2268115	rs2216128	rs220557	rs220567	rs10845847	rs12828473	rs10772722	rs7966469	rs7310659
1	A G	G G	A G	0 0	T T	T T	A C	0 0	C C	G G	G T	C C	G G
2	A A	G G	G G	0 0	T T	T T	A C	0 0	A A	A G	G T	C T	A G
3	A G	C C	A A	0 0	G T	C T	A C	0 0	A C	A G	T T	C C	G G
4	A A	G G	G G	0 0	T T	C T	A A	0 0	C C	G G	G G	C T	A G
5	A G	C G	A A	0 0	T T	T T	A A	0 0	C C	A G	G T	C T	A G
6	A A	G G	G G	0 0	G G	T T	C C	0 0	A A	A A	G G	C C	A G
7	A G	C G	A G	0 0	G T	C T	A A	0 0	A A	A G	G T	C C	A G
8	0 0	0 0	A G	0 0	G G	T T	0 0	0 0	0 0	A G	G T	C C	0 0
9	A G	G G	A A	0 0	G G	T T	A A	0 0	C C	G G	G T	C C	G G
10	A G	C G	A G	0 0	G G	C T	A C	0 0	0 0	A G	T T	C T	A G
11	G G	C C	A A	0 0	T T	T T	A A	0 0	C C	G G	G T	T T	A A
12	A A	0 0	A A	0 0	G T	C T	C C	0 0	0 0	0 0	0 0	C C	A G
13	A A	C G	A A	0 0	G T	C C	A C	0 0	C C	A G	G G	C T	G G
14	A G	G G	G G	0 0	G G	C T	A C	0 0	A C	A G	T T	C C	G G
15	A G	C G	A G	0 0	G G	T T	A A	0 0	C C	A G	G G	C C	G G
16	G G	C G	A G	0 0	G T	T T	A C	0 0	A C	A A	G T	C C	A G
17	G G	C C	A A	0 0	T T	T T	A A	0 0	C C	A G	T T	C C	G G
18	A G	G G	G G	0 0	G T	T T	A A	0 0	C C	G G	T T	C C	G G
19	A G	C C	A A	0 0	G G	T T	A C	0 0	A C	A G	G G	C T	A G
20	A G	G G	G G	0 0	G T	T T	A C	0 0	A A	A G	G G	C C	A G
21	A A	C G	A G	0 0	G T	C T	A C	0 0	A A	A G	G T	C C	G G
22	A A	C G	A G	0 0	G T	C T	A A	0 0	A C	A A	G T	C T	A G
23	A A	G G	G G	0 0	T T	T T	A A	0 0	C C	A A	G T	C T	A G
24	A A	G G	G G	0 0	T T	T T	A A	0 0	A A	A A	G T	C C	A G
25	A G	G G	A G	0 0	T T	T T	A A	0 0	A C	A A	G T	C T	A A
26	A A	C G	A G	0 0	T T	C C	A A	0 0	C C	G G	G T	T T	A A
27	G G	C C	A A	0 0	T T	C T	A A	0 0	C C	G G	G T	C C	G G
28	A A	G G	G G	0 0	G T	T T	A C	0 0	A A	A G	G G	C C	G G
29	A A	G G	A G	0 0	G T	C T	A C	0 0	C C	G G	G G	C T	A A

30	A G	C G	A G	0 0	G T	C T	A C	0 0	A C	A G	G T	T T	A A
31	A G	G G	A G	0 0	G T	T T	A A	0 0	A A	A G	T T	C C	G G
32	G G	C C	A A	0 0	G T	C T	A A	0 0	C C	A A	G G	C T	A G
33	A A	G G	G G	0 0	G T	T T	A C	0 0	A A	A G	G T	C T	A G
34	A A	G G	G G	0 0	T T	T T	A A	0 0	C C	A G	G G	C C	G G
35	G G	C G	A G	0 0	G T	C T	A A	0 0	C C	A G	G T	T T	A A
36	A G	C G	A G	0 0	T T	T T	A A	0 0	C C	G G	T T	C C	G G
37	0 0	0 0	G G	0 0	G T	T T	0 0	0 0	0 0	0 0	0 0	C C	G G
38	A A	G G	A G	0 0	G T	T T	A C	0 0	A A	A A	G G	T T	A A
39	0 0	0 0	A G	0 0	0 0	C T	0 0	0 0	0 0	0 0	0 0	C C	0 0
40	G G	C C	A A	0 0	G T	C T	C C	0 0	A A	A G	G T	C T	G G
41	0 0	0 0	A G	0 0	0 0	T T	0 0	0 0	0 0	0 0	0 0	C C	0 0
42	A G	C C	A A	0 0	T T	T T	A A	0 0	C C	A A	G T	C T	G G
43	0 0	0 0	A A	0 0	0 0	C T	0 0	0 0	0 0	0 0	0 0	C C	0 0
44	G G	G G	G G	0 0	T T	T T	A C	0 0	A A	A A	T T	C C	G G
45	0 0	0 0	A G	0 0	G T	T T	0 0	0 0	0 0	0 0	0 0	T T	A G
46	G G	C G	A A	0 0	G G	C T	A A	0 0	C C	G G	T T	C C	G G
47	A A	G G	A G	0 0	G T	C T	0 0	0 0	0 0	0 0	G G	C C	A G
48	A G	C G	A G	0 0	T T	T T	A A	0 0	C C	G G	G G	C C	G G
49	A G	C G	A G	0 0	G T	T T	A A	0 0	0 0	A G	G G	C C	G G
50	0 0	0 0	A G	0 0	0 0	C T	0 0	0 0	0 0	0 0	0 0	C C	0 0
51	A A	G G	G G	0 0	G T	T T	A C	0 0	A A	A G	T T	C C	G G
52	0 0	0 0	A A	0 0	0 0	C T	0 0	0 0	0 0	0 0	0 0	C C	0 0
53	A G	G G	A G	0 0	T T	T T	A C	0 0	C C	A G	G T	C C	G G
54	0 0	0 0	A A	0 0	G T	C T	0 0	0 0	A A	0 0	G G	C T	A G
55	A G	C G	A G	0 0	G T	T T	A A	0 0	C C	G G	G G	C C	G G
56	0 0	0 0	A A	0 0	T T	T T	0 0	0 0	0 0	0 0	0 0	C C	G G
57	A A	G G	G G	0 0	G G	T T	A A	0 0	C C	G G	G T	C C	G G
58	A G	C G	A G	0 0	T T	T T	A A	0 0	A A	A G	T T	C T	A G
59	A A	G G	G G	0 0	G T	T T	A A	0 0	C C	G G	T T	C T	A G
60	G G	C G	A G	0 0	G G	T T	A C	0 0	A A	A A	G G	C C	G G
61	A A	C G	A A	0 0	G T	T T	A C	0 0	A A	A A	G G	C C	A G
62	0 0	0 0	A G	0 0	G T	C T	0 0	0 0	0 0	0 0	0 0	C T	A G

63	A A	C G	A G	0 0	T T	T T	A A	0 0	A A	A G	G T	C C	A G
64	A G	C C	A A	0 0	G T	C T	A A	0 0	A C	A G	G T	C T	A G
65	A A	G G	G G	0 0	G G	C T	A C	0 0	A C	A G	G G	C T	G G
66	0 0	0 0	A A	0 0	T T	C T	0 0	0 0	0 0	0 0	0 0	C C	A G
67	A G	C G	A A	0 0	G G	C T	A C	0 0	A C	G G	G T	C C	A G
68	A G	G G	A G	0 0	G T	T T	A C	0 0	A A	A G	G G	C T	A G
69	A A	G G	A G	0 0	G G	T T	A C	0 0	A C	A G	G G	C C	G G
70	A A	G G	A G	0 0	T T	T T	A A	0 0	0 0	A G	G T	C C	A G
71	G G	C G	A G	0 0	G T	C T	A C	0 0	A C	A G	G T	C C	G G
72	A A	C G	A G	0 0	G T	T T	A A	0 0	C C	G G	G G	C C	A G
73	G G	C G	A G	0 0	G T	T T	A C	0 0	A C	A G	G T	C T	A A
74	A A	G G	G G	0 0	G T	T T	A C	0 0	A A	A G	T T	C C	G G
75	A A	C G	A G	0 0	T T	T T	A C	0 0	A C	A A	T T	C C	G G
76	A A	G G	G G	0 0	T T	C T	A A	0 0	C C	G G	G T	C T	A G
77	A A	G G	A G	0 0	G G	T T	A A	0 0	C C	A G	G T	C T	A G
78	A G	C C	A A	0 0	G T	C T	A A	0 0	C C	G G	G T	C C	G G
79	A A	G G	A G	0 0	G T	T T	A C	0 0	A C	A G	G G	T T	A A
80	A G	C G	A G	0 0	G G	T T	A C	0 0	A C	A A	G T	C C	G G
81	A G	C G	A A	0 0	G T	T T	A C	0 0	A C	A G	T T	C C	G G
82	A G	C G	A G	0 0	T T	T T	A A	0 0	0 0	A G	G T	T T	A A
83	A G	C G	A G	0 0	G T	C T	A C	0 0	A C	A G	G G	C T	A G
84	G G	C G	A A	0 0	G T	T T	A C	0 0	A C	A A	G T	C T	A A
85	A A	G G	G G	0 0	G G	T T	A A	0 0	C C	G G	G T	C C	G G
86	A G	C G	A A	0 0	G T	C T	A C	0 0	A C	A A	G T	C C	G G
87	A G	G G	A G	0 0	G T	T T	A A	0 0	C C	A G	G T	C C	G G
88	AA	GG	AG	0 0	TT	CT	AA	0 0	CC	AG	GT	CT	AG
89	G G	C G	0 0	0 0	G T	0 0	A A	0 0	C C	A A	G G	C T	A A
90	A A	G G	A G	0 0	G T	C T	A C	C G	A C	A G	G G	T T	A A
91	A G	C G	A A	0 0	G T	C T	A C	C G	A C	A G	T T	C C	G G
92	A A	G G	A G	0 0	G T	C T	A C	C G	A A	A G	T T	T T	A A
93	G G	C C	A A	0 0	T T	T T	A C	G G	A A	A G	G T	C T	A G
94	A A	C G	A A	0 0	G G	C C	C C	G G	A A	A A	T T	T T	A A
95	A G	G G	G G	0 0	G T	T T	A C	C G	A C	A A	G G	T T	A A

96	G G	G G	G G	0 0	G G	T T	C C	G G	A A	A A	G T	C C	G G
97	A G	G G	G G	0 0	T T	T T	A A	C C	C C	A A	T T	C C	G G
98	G G	C C	A A	0 0	G T	T T	A A	C C	C C	A G	G T	C C	A G
99	A G	C G	A A	0 0	G G	C T	A C	G G	A C	A A	G T	C T	A G
100	0 0	0 0	G G	0 0	0 0	T T	0 0	C G	0 0	0 0	0 0	C C	0 0
101	A G	C G	A G	0 0	G G	C T	A C	C G	A C	A G	G T	C T	A A
102	G G	C C	A A	0 0	T T	T T	A A	C C	C C	G G	T T	C T	A G
103	G G	C C	A A	0 0	T T	T T	A A	C G	C C	G G	G G	0 0	A A
104	A G	C G	A G	0 0	G T	T T	A C	C G	A C	A G	T T	T T	A A
105	A A	G G	A G	0 0	T T	T T	A A	G G	C C	G G	G T	C T	A G
106	A A	G G	G G	0 0	G T	T T	A A	C C	C C	G G	G G	C C	G G
107	A G	G G	G G	0 0	G T	T T	A C	C G	A C	A A	G G	T T	A A
108	A G	C G	A G	0 0	G G	C C	A C	G G	A C	A A	G G	C C	G G
109	A A	C G	A G	0 0	G T	T T	A C	C G	A A	A G	G T	C T	A G
110	A G	C G	A G	0 0	T T	T T	A C	C G	A C	G G	G T	C T	A A
111	G G	C G	A G	0 0	G T	C T	A C	C G	A C	A G	G G	C C	G G
112	A G	C G	A G	0 0	T T	T T	A A	C G	C C	G G	G G	C C	G G
113	A G	G G	G G	0 0	G T	T T	A A	G G	C C	A G	G T	C T	A G
114	A G	G G	G G	0 0	G G	C T	C C	G G	A A	A A	T T	C T	A G
115	A G	C G	A G	0 0	G G	C T	A A	C G	A C	A G	T T	C C	G G
116	A G	C G	A G	0 0	T T	T T	A C	C G	C C	A G	G G	C T	A G
117	A G	C G	A G	0 0	T T	T T	A A	C G	C C	G G	G G	C C	A G
118	A G	G G	A G	0 0	T T	T T	A A	C C	C C	G G	G G	C T	A G
119	A G	C G	A G	0 0	G T	T T	A C	G G	A C	A A	T T	C C	G G
120	G G	C G	A G	0 0	G T	T T	A C	C G	A A	A G	G T	C T	A A
121	A A	G G	G G	0 0	G T	T T	A C	C G	A A	A G	G T	C C	A G
122	A A	G G	G G	0 0	G T	T T	A A	C G	C C	A A	G T	C T	A G
123	A A	G G	G G	0 0	G T	T T	A C	G G	A A	A A	G T	C T	A A
124	G G	C G	A G	0 0	G T	T T	C C	G G	A A	A A	G T	C C	G G
125	0 0	0 0	A A	0 0	G T	T T	0 0	G G	A A	A A	0 0	C C	0 0
126	G G	C G	A A	0 0	T T	T T	A A	C G	C C	G G	G T	C T	A G
127	A G	G G	A G	0 0	G T	C T	A C	G G	A A	A A	G T	C T	A G
128	A G	C G	A G	0 0	G G	C T	A A	C G	A C	A G	G T	C T	A G

129	G G	0 0	A A	0 0	G T	T T	0 0	0 0	C C	A A	T T	T T	0 0
130	A G	C C	A A	0 0	G T	T T	A A	C C	C C	A G	G G	C C	A G
131	G G	C C	A A	0 0	T T	T T	0 0	G G	A A	0 0	0 0	C T	0 0
132	A A	G G	G G	0 0	G T	T T	A A	C G	C C	A G	G G	C C	A G
133	A G	C G	A A	0 0	T T	0 0	A C	C C	C C	G G	G T	0 0	A G
134	A A	C G	A A	0 0	G T	T T	A A	C G	C C	A G	G G	C T	A G
135	0 0	0 0	A G	0 0	G G	C T	0 0	G G	A C	0 0	G G	C C	0 0
136	A G	G G	A G	0 0	G T	T T	A C	C G	A A	A G	G G	C C	G G
137	A G	G G	A G	0 0	G G	T T	A A	C G	A A	A G	G T	C C	G G
138	A A	G G	G G	0 0	T T	T T	A A	C G	C C	A A	G T	C C	G G
139	A A	C G	A G	0 0	G T	C T	A C	G G	A C	A G	T T	C C	A G
140	A G	C G	A A	0 0	T T	0 0	A A	C C	C C	G G	G T	C T	A A
141	A G	C G	G G	0 0	G T	T T	C C	G G	A A	A A	T T	C T	A A
142	A A	G G	G G	0 0	T T	T T	A A	G G	C C	G G	G T	T T	A A
143	A G	C C	A A	0 0	T T	T T	A A	C G	C C	G G	T T	C C	G G
144	A A	G G	G G	0 0	G T	C T	A C	C G	A C	A G	G T	C T	A G
145	G G	C C	A A	0 0	G G	C T	C C	G G	A A	G G	G T	C C	G G
146	A G	C G	A A	0 0	G T	T T	A A	C G	C C	A A	G T	C C	A G
147	A G	G G	G G	0 0	G T	T T	C C	G G	A A	A A	G G	0 0	G G
148	A G	C C	A A	0 0	G T	C C	A C	G G	A C	A G	G G	C C	G G
149	A A	G G	A G	0 0	G T	T T	A C	G G	A A	A A	G T	C C	G G
150	G G	G G	A G	0 0	G T	C T	0 0	C G	0 0	0 0	G G	C T	0 0
151	A A	G G	A G	0 0	T T	C T	A A	C G	C C	G G	G T	C C	A G
152	A A	C G	A G	0 0	G T	T T	A C	C G	A A	A G	G T	C T	A G
153	A A	G G	A A	0 0	G G	T T	A C	G G	A C	A G	G G	C T	A G
154	A A	G G	A A	0 0	G T	T T	A C	C C	A A	A G	G T	C T	A G
155	A A	G G	G G	0 0	T T	C T	A A	G G	C C	G G	G T	C C	G G
156	A G	C G	A A	0 0	G T	T T	A A	C G	C C	G G	G G	C C	A G
157	A A	G G	G G	0 0	G G	C T	A C	C G	A C	A G	G T	C T	A G
158	A G	C G	0 0	0 0	T T	C T	0 0	0 0	A C	G G	G T	C C	G G
159	A A	C G	A G	0 0	T T	T T	A C	C G	A A	A G	G G	C C	G G
160	0 0	0 0	0 0	0 0	0 0	C T	0 0	C G	0 0	0 0	0 0	C C	0 0
161	A A	G G	G G	0 0	G T	T T	A C	0 0	A A	A G	G T	C C	G G

162	G G	C C	A A	0 0	G T	C T	A C	C G	A C	A G	G T	C C	A G
163	A G	C C	A A	0 0	G T	C T	A C	C G	C C	G G	G T	C T	A G
164	A A	G G	A G	0 0	G T	C T	A C	C G	A A	A G	G T	C T	A G
165	A A	G G	A G	0 0	G T	T T	A A	C C	C C	A G	G T	C C	A G
166	A G	C G	A A	0 0	G T	T T	A C	C G	A A	G G	G G	C T	A A
167	A A	G G	A G	0 0	T T	T T	A A	C G	C C	G G	G G	C T	G G
168	A A	G G	A A	0 0	G T	T T	A A	C C	C C	G G	G G	C C	A G
169	A G	C G	A G	0 0	G T	C T	A C	G G	A C	A G	G G	C C	A G
170	A G	C G	A G	0 0	G T	C T	A C	C G	A C	G G	G T	C C	A G
171	G G	C C	A A	0 0	G G	C C	A C	G G	A C	A G	G G	C C	A G
172	A G	C G	0 0	0 0	G T	0 0	A A	0 0	A C	0 0	G T	C T	0 0
173	G G	G G	A A	0 0	T T	T T	A A	G G	C C	G G	G T	C T	A G
174	A A	G G	G G	0 0	T T	T T	A C	G G	C C	A G	G G	C C	G G
175	A A	G G	G G	0 0	G T	T T	A A	C G	C C	G G	G G	C C	G G
176	A G	G G	A G	0 0	T T	T T	A A	C G	C C	G G	G G	C C	G G
177	G G	C G	A A	0 0	G T	C T	A A	0 0	A A	A G	G G	C C	G G
178	G G	C G	A G	0 0	G T	T T	A A	0 0	0 0	A G	G G	C C	G G
179	A A	G G	G G	0 0	T T	T T	A C	0 0	0 0	A G	G T	C C	A G
180	A G	G G	A G	0 0	T T	C T	A A	0 0	C C	A A	G G	C T	G G
181	A G	C G	A G	0 0	G T	C T	A A	0 0	0 0	G G	G T	C C	A G
182	A G	C G	A G	0 0	T T	T T	A A	0 0	C C	G G	G G	C C	A G
183	G G	G G	A A	0 0	G T	T T	A A	0 0	C C	A A	G T	C T	A G
184	A G	C G	A G	0 0	T T	T T	A A	0 0	C C	G G	T T	C T	A G
185	A G	C G	A A	0 0	T T	T T	A A	0 0	0 0	G G	T T	0 0	G G
186	A G	C G	A G	0 0	G T	C T	A C	0 0	A C	A A	G T	C T	A G
187	A A	C G	A A	0 0	G G	T T	C C	0 0	A A	A G	G G	C T	A G
188	A G	C G	A A	0 0	G T	T T	A C	0 0	A A	A A	G G	C T	A G
189	A G	G G	A G	0 0	G G	T T	A C	0 0	A C	A A	G T	C C	G G
190	A A	G G	G G	0 0	G T	T T	A A	0 0	0 0	G G	T T	C T	A G
191	A A	G G	G G	0 0	T T	C T	A A	0 0	C C	G G	0 0	C C	A G
192	A A	G G	A A	0 0	G G	C T	A C	0 0	A C	A G	G T	C T	A G
193	A G	C G	A G	0 0	G T	C C	C C	0 0	A A	A A	G G	C C	A G
194	G G	G G	A G	0 0	T T	T T	A C	0 0	A C	A G	G T	C T	A G

195	A A	G G	A G	0 0	T T	T T	A A	0 0	C C	G G	G T	C T	A A
196	A A	G G	G G	0 0	G G	T T	A C	0 0	A C	A G	G T	T T	A A
197	G G	C G	A A	0 0	T T	T T	A A	0 0	C C	A A	G T	C T	A G
198	A G	C G	0 0	0 0	G G	C C	C C	0 0	A A	A A	G T	T T	A A
199	A G	C C	A A	0 0	G G	C T	A C	0 0	A A	A A	G G	C T	A A
200	A G	C G	A G	0 0	G T	T T	A A	0 0	C C	A G	T T	C C	G G
201	A G	C G	A G	0 0	G T	C T	A C	0 0	0 0	A G	G T	C T	A G
202	A G	G G	G G	0 0	G T	T T	A C	0 0	A C	A G	T T	C C	G G
203	A G	C C	A A	0 0	G T	C T	C C	0 0	A A	A A	T T	C C	G G
204	A G	C G	A A	0 0	G T	T T	C C	0 0	A A	A A	0 0	C C	A G
205	A A	C G	A G	0 0	G T	T T	A C	0 0	A C	A G	T T	C C	G G
206	A G	C G	A G	0 0	G G	T T	A A	0 0	C C	G G	G G	C C	G G
207	A G	G G	A G	0 0	G T	T T	A A	0 0	C C	G G	T T	C C	A A
208	G G	G G	A G	0 0	G T	T T	A C	0 0	A C	A G	G T	C C	G G
209	G G	G G	A G	0 0	G T	T T	A C	0 0	A C	A G	G T	C C	G G
210	A G	C G	A A	0 0	T T	C T	C C	0 0	A A	A A	G T	C C	G G
211	A G	C G	A G	0 0	G T	T T	A C	0 0	A A	A G	G T	C C	G G
212	A G	G G	A A	0 0	G T	T T	A C	0 0	A C	A G	G G	C T	A G
213	A G	C G	A G	0 0	T T	C T	A C	0 0	A A	A A	G T	C C	A G
214	A A	C G	A G	0 0	G G	C T	A C	0 0	A C	A A	G T	C C	G G
215	G G	C C	A A	0 0	G T	C T	A C	0 0	A C	A G	G G	C C	G G
216	A A	G G	G G	0 0	T T	C T	A A	0 0	A A	A A	T T	C C	G G
217	G G	C G	A A	0 0	G G	C C	A C	0 0	A C	A G	G G	C T	A G
218	G G	C G	A G	0 0	G T	T T	C C	0 0	A A	A A	T T	C C	A G
219	A A	C G	A G	0 0	G T	C T	A C	0 0	A C	A G	G T	C T	A G
220	G G	C C	A A	0 0	T T	T T	A A	0 0	C C	G G	G T	C T	A G
221	A G	G G	G G	0 0	G G	T T	A A	0 0	C C	A G	G T	C T	A A
222	A G	C G	A A	0 0	T T	C T	C C	0 0	A A	A A	G T	C C	G G
223	A G	C G	A A	0 0	G T	C T	A C	0 0	A C	A G	G T	C T	A G
224	G G	C G	A G	0 0	G T	0 0	A A	0 0	C C	A G	G T	C T	A G
225	A G	G G	G G	0 0	G T	T T	A C	0 0	A A	A G	G T	C C	G G
226	G G	C C	A A	0 0	G G	C C	A A	0 0	C C	A A	G G	C C	G G
227	G G	C C	A A	0 0	G T	C T	A C	0 0	A C	A G	G T	C C	G G

228	A A	G G	A G	0 0	G T	T T	A C	0 0	A C	A G	G T	C C	G G
229	A G	G G	A G	0 0	G T	T T	A C	0 0	A C	A G	T T	C T	A G
230	A G	C G	A A	0 0	G T	C T	A C	0 0	A C	A G	G T	C T	A G
231	G G	G G	A G	0 0	G T	T T	A A	0 0	C C	G G	G T	C C	A G
232	A G	G G	A A	0 0	G T	C T	C C	0 0	A A	A A	G T	C C	G G
233	G G	C C	A A	0 0	G G	C T	A C	0 0	A C	A A	G T	C C	A A
234	A G	C G	A G	0 0	G G	T T	C C	0 0	A A	A A	T T	C C	G G
235	A G	C G	A G	0 0	G T	C T	A A	0 0	C C	G G	G T	C T	A G
236	A G	0 0	A G	0 0	G T	T T	A C	0 0	C C	G G	G T	C C	A G
237	A G	C G	A G	0 0	G T	T T	A A	0 0	C C	G G	G T	C C	G G
238	A G	C G	A G	0 0	T T	T T	A C	0 0	A A	A G	T T	C C	G G
239	A G	G G	A G	0 0	G T	T T	A C	0 0	A C	A G	G G	C T	A G
240	A G	C G	A A	0 0	T T	T T	C C	0 0	A A	A A	G G	C T	A G
241	A G	C G	A G	0 0	G G	C T	C C	0 0	A C	A A	G G	C C	A G
242	A A	C G	A G	0 0	G T	C T	A A	0 0	C C	G G	G T	C C	A G
243	A G	C G	A G	0 0	G T	C T	A A	0 0	C C	G G	T T	C T	A G
244	A A	C G	A G	0 0	G G	T T	A C	0 0	A A	A A	T T	C T	A G
245	G G	C G	A G	0 0	G G	C T	C C	0 0	A A	A A	G T	C C	G G
246	G G	C G	A A	0 0	G G	C T	A C	0 0	A A	A G	G T	C C	G G
247	A G	G G	G G	0 0	G G	C T	A C	0 0	A C	A G	G G	C C	A G
248	A G	C G	A G	0 0	G T	T T	A A	0 0	C C	G G	T T	C C	G G
249	A G	C G	A A	0 0	T T	C T	C C	0 0	A C	A G	T T	C T	A G
250	A G	C G	A G	0 0	G T	T T	A A	0 0	C C	G G	T T	C C	G G
251	G G	C G	A G	0 0	G T	T T	A C	0 0	A C	A A	G T	C C	G G
252	A G	C C	A A	0 0	G T	T T	A A	0 0	C C	G G	G T	C C	G G
253	A G	G G	G G	0 0	T T	T T	A A	0 0	C C	A G	G G	C T	A A
254	A G	C G	A G	0 0	T T	T T	A A	0 0	C C	G G	G G	T T	A A
255	A G	C G	A G	0 0	G T	T T	A A	0 0	C C	A G	G T	C C	G G
256	A G	C C	A A	0 0	G G	C T	A C	0 0	A A	A G	T T	C C	A A
257	A A	G G	A G	0 0	G T	C T	A C	0 0	A C	A G	G T	C T	A G
258	A G	C G	A G	0 0	G G	C C	A A	0 0	0 0	G G	G T	C C	G G
259	G G	C G	A G	0 0	G T	T T	A A	0 0	C C	G G	G T	T T	G G
260	A G	G G	G G	0 0	G T	T T	A A	0 0	C C	G G	G T	C C	G G

261	G G	C C	0 0	0 0	T T	0 0	A A	0 0	C C	G G	G T	C C	A G
262	A G	C G	A G	0 0	G T	T T	A A	0 0	C C	G G	G G	C T	A G
263	A G	G G	G G	0 0	G T	C T	A C	0 0	A A	A G	G T	C C	G G
264	A G	G G	A G	0 0	G T	T T	C C	0 0	A A	A A	G T	C T	A G
265	G G	C C	A A	0 0	G T	C T	A A	0 0	C C	A G	G G	C T	A A
266	A A	G G	G G	0 0	G T	T T	A C	0 0	A A	A G	G T	C C	A G
267	G G	C G	A A	0 0	G T	T T	A A	0 0	C C	A A	T T	C T	G G
268	A G	G G	A G	0 0	G G	C T	A C	0 0	A C	G G	G T	C C	G G
269	A G	C G	A A	0 0	G T	T T	A C	0 0	A C	A G	G G	C C	A G
270	A G	G G	G G	0 0	G T	T T	C C	0 0	A C	A A	G T	C C	A G
271	G G	G G	A G	0 0	T T	C T	A A	0 0	C C	A G	G T	C C	A G
272	A G	C G	A A	0 0	G T	T T	A A	0 0	C C	A G	G T	C C	A G
273	G G	C C	A A	0 0	G T	C T	A A	0 0	C C	G G	G G	C T	G G
274	A G	C C	A A	0 0	G T	C T	A C	0 0	A C	A G	G T	C C	G G
275	A A	G G	A G	0 0	G T	C T	A C	0 0	A C	A G	T T	C C	G G
276	A A	C G	0 0	0 0	T T	0 0	A C	0 0	A A	A G	T T	C C	G G
277	A G	G G	A A	0 0	G T	T T	A C	0 0	A C	A G	G G	C C	G G
278	G G	C C	A A	0 0	G T	C T	A C	0 0	C C	A G	G T	T T	A A
279	G G	C C	A A	0 0	T T	T T	A A	0 0	A A	A A	T T	C C	A G
280	A G	C G	A G	0 0	G T	T T	A C	0 0	A A	A G	T T	C C	A G
281	A G	G G	A G	0 0	G G	C T	A C	0 0	A C	A G	T T	C T	A G
282	A G	C G	A A	0 0	G G	C T	A C	0 0	A C	A G	G T	C C	G G
283	A G	G G	A G	0 0	T T	C T	A A	0 0	A C	A G	G T	C C	G G
284	A G	C G	A G	0 0	T T	C T	A C	0 0	A A	A A	G T	C T	A A

GENE	GRIN2B-ATF7IP	ATF7IP	ATF7IP	ATF7IP	ATF7IP	ATF7IP	ATF7IP	ATF7IP	ATF7IP	ATF7IP	ATF7IP	SP1	SP1
SNP/Sample	rs10845905	rs10845923	rs4341624	rs4764074	rs11055896	rs10845943	rs10744055	rs10845987	rs2231909	rs3213764	rs11055989	rs7131938	rs3741651
1	A A	C C	0 0	A G	C C	G G	C C	C T	A T	A G	A A	C T	A G
2	A G	C C	C C	G G	C G	G G	A C	C T	A T	A G	A G	C C	A A
3	A A	C C	C C	A G	C G	G G	A A	C C	A A	G G	G G	C T	A G
4	A G	C T	C T	A A	G G	G G	A A	C C	A A	G G	G G	C C	A A
5	A G	C C	C T	A A	G G	C G	A C	C C	A A	G G	A G	C T	G G
6	A G	C C	C C	A G	C G	G G	A C	C T	A T	A G	A A	C C	A A
7	A A	C T	T T	A G	C G	G G	A C	T T	A T	A A	A A	T T	A A
8	A A	T T	T T	G G	C G	C G	C C	C C	A A	G G	G G	C C	A G
9	A A	C T	C T	G G	C G	G G	A C	C T	A A	A G	A A	C T	A G
10	A G	C T	C T	A G	C G	G G	A C	C C	A A	G G	A G	C C	A A
11	G G	C C	C C	A A	G G	C G	A C	C T	A T	A G	A G	C T	A G
12	A A	C T	C T	A G	C G	G G	A C	C T	A A	A G	A G	0 0	A G
13	A G	C C	C T	A G	G G	C G	A C	T T	T T	A A	A A	C C	A A
14	G G	C C	T T	A G	C G	G G	A C	C T	A T	A G	A G	C C	A A
15	A G	C C	C T	A G	G G	C G	A C	C C	A A	G G	G G	C C	A A
16	A G	C T	T T	A G	C G	G G	A C	T T	A T	A A	A A	T T	A G
17	A G	C C	C T	A A	G G	C C	C C	C T	A T	G G	A G	C C	A A
18	A G	C T	T T	A G	C G	C G	C C	C C	A A	G G	A A	C C	A A
19	A G	C C	C C	A G	C G	G G	A C	C T	A T	A G	A G	C C	A A
20	A A	C T	C T	G G	G G	G G	A A	T T	A T	A A	A A	C C	A A
21	A G	C C	C T	A G	C G	C G	C C	C T	A T	A G	A A	C C	A A
22	A G	C T	C T	A G	C G	G G	A A	C C	A A	G G	G G	C C	A A
23	A G	C T	C T	A G	C G	G G	A C	T T	T T	A A	A A	C C	A A
24	A A	C T	C T	G G	C C	G G	C C	C C	A A	G G	A G	C T	A G
25	A G	C T	C T	A G	G G	G G	A A	C T	A T	A G	A G	C C	A A
26	G G	C C	C C	G G	C G	G G	A C	T T	T T	A A	A A	C C	A A
27	A A	C C	C C	G G	C C	G G	C C	T T	T T	A A	A A	C C	A A
28	A A	C C	C C	A G	C G	G G	A C	C C	A A	G G	A G	C T	A G
29	G G	C C	C C	A A	G G	G G	A A	C T	A T	A G	A G	T T	G G

30	G G	C C	C C	G G	C C	G G	C C	T T	T T	A A	A A	C T	A G
31	A G	C C	C T	A A	G G	C G	A C	C T	A T	A G	A G	C C	A A
32	G G	C C	C T	A G	C G	G G	A C	C T	A T	A G	A G	C C	A A
33	A G	C C	C C	G G	C C	G G	C C	T T	T T	A A	A A	C T	A A
34	A A	C T	C T	G G	C G	G G	A C	C C	A A	G G	G G	C C	A A
35	G G	C C	C C	A A	G G	C C	C C	C T	A T	A G	A A	C C	A A
36	A A	C T	C T	G G	G G	C G	A C	C T	A A	A G	A G	C C	A G
37	A A	C T	C T	A A	G G	G G	A A	C C	A A	G G	G G	0 0	A A
38	G G	C C	C C	A A	G G	C G	A C	C T	A T	A G	A G	C C	A A
39	0 0	0 0	C C	A G	G G	C G	A C	C T	A T	A G	A G	0 0	0 0
40	A A	C T	C T	G G	G G	C G	A C	C C	A A	G G	A G	T T	A G
41	0 0	0 0	C T	A G	G G	C G	A C	C T	A T	A G	A A	0 0	0 0
42	A G	C C	C T	G G	G G	G G	A A	T T	T T	A A	A A	C C	A A
43	0 0	0 0	C C	A G	G G	G G	A A	C C	A A	G G	G G	0 0	0 0
44	A A	C C	C C	G G	C G	G G	A C	T T	T T	A A	A A	C C	A G
45	A G	C C	C C	A G	C C	G G	A C	C T	A T	A G	A A	T T	G G
46	A G	C C	C T	G G	G G	C G	A C	T T	A A	A A	A A	C T	A G
47	A A	C T	C T	A G	C G	C G	C C	C T	A T	A G	A G	0 0	A A
48	A G	C C	C T	A A	G G	C G	0 0	C T	A T	A G	A G	C C	A A
49	A A	C C	C C	G G	C G	C C	C C	C T	A T	A G	A A	C T	A G
50	0 0	0 0	C T	A G	G G	C C	C C	T T	A T	A A	A A	0 0	0 0
51	A A	C C	C C	G G	G G	C G	A C	C T	A A	A G	A G	C C	A A
52	0 0	0 0	C T	G G	C G	G G	C C	C T	A A	A G	A A	0 0	0 0
53	A G	C T	T T	A G	G G	G G	A A	T T	A T	A A	A A	C C	A A
54	A G	C T	C T	G G	C G	G G	A C	T T	T T	A G	A G	0 0	A A
55	A A	C C	C C	G G	C G	C G	A C	C C	A A	G G	A G	C C	A A
56	A A	C T	C T	A G	G G	C G	A A	C T	A T	A G	A G	0 0	A A
57	A G	C C	C T	A G	G G	C G	A C	T T	A T	A A	A A	C T	A G
58	G G	C C	C T	A G	C G	G G	A C	C T	A T	A G	A G	C T	A A
59	A G	C C	C C	A G	G G	G G	A A	C T	A A	A G	A G	C C	A A
60	A A	C C	C C	G G	C G	C G	C C	T T	A T	A A	A A	C C	A A
61	A G	C C	C C	A G	G G	G G	A A	C T	A T	A G	A G	T T	G G
62	A G	C C	C C	A G	G G	G G	A A	T T	T T	A A	A A	0 0	A A

63	A A	C T	C T	G G	C C	G G	A C	C T	A T	A G	A A	C T	A G
64	A G	C C	C C	G G	C C	G G	0 0	C C	0 0	A G	0 0	C T	A G
65	A A	C C	C C	G G	G G	G G	A A	T T	T T	A A	A A	C T	A G
66	A A	T T	0 0	G G	G G	G G	0 0	C C	0 0	A A	0 0	0 0	A A
67	A G	C C	C C	A A	G G	G G	A A	C C	A A	G G	G G	C C	A G
68	G G	C C	C T	A G	G G	G G	A A	C T	A T	A G	A G	C T	A G
69	A A	C T	C T	G G	C C	G G	C C	C T	A T	A G	A A	T T	G G
70	A G	C T	C T	0 0	G G	G G	A A	C T	A A	A G	A G	C C	A A
71	A A	C T	C T	G G	C G	G G	A A	C T	A T	A G	A A	C C	A A
72	A G	C T	C T	A G	C G	C G	C C	T T	T T	A A	A A	C T	A G
73	A G	C T	C T	A G	C G	G G	A C	T T	T T	A G	A G	C T	A G
74	A A	C T	C T	A G	G G	G G	A A	C C	A A	G G	G G	C C	A A
75	A A	C T	C T	G G	C C	G G	C C	C T	A T	A A	A A	C C	A A
76	G G	C C	C T	G G	C G	G G	A C	T T	A T	A G	A A	C T	A G
77	A G	C C	C C	A G	G G	C G	A C	C T	A A	A G	A G	C T	A A
78	A A	C C	C C	A G	C G	G G	A C	C T	A A	A G	A G	C C	A A
79	G G	C C	C C	A G	G G	C G	A C	T T	A T	A A	A A	C T	A G
80	A A	C T	C T	A G	G G	G G	A A	C T	A A	A G	A G	T T	G G
81	A A	C C	C C	A G	G G	C G	A C	T T	A T	A A	A A	C T	A G
82	G G	C C	C C	A A	C G	G G	A C	C T	A T	A G	A G	C C	A A
83	A G	C C	C C	G G	C G	G G	A C	T T	A T	A G	A G	C C	A A
84	A G	C T	C T	A G	C G	G G	A C	C T	A T	A G	A G	C C	A A
85	A G	C T	T T	A G	G G	G G	A A	T T	A A	A A	A A	C T	A A
86	A A	C T	C T	G G	C G	G G	A A	T T	A T	A A	A A	C T	A G
87	A A	C C	C C	A G	C G	C G	C C	C C	A A	G G	A A	C C	A A
88	G G	C C	C T	A A	C G	C G	C C	C T	A T	A G	A A	C T	A G
89	A G	C T	0 0	0 0	0 0	0 0	0 0	0 0	0 0	0 0	0 0	C T	A G
90	G G	C C	T T	G G	C C	G G	A A	C T	A A	A G	A A	C C	A A
91	A A	T T	T T	G G	C G	G G	A C	C C	A A	G G	A G	C T	A G
92	G G	C C	C C	A G	G G	G G	A A	C C	A A	A G	A G	C C	A A
93	A G	C T	C T	G G	C G	C G	C C	T T	T T	A A	A A	C C	A A
94	G G	C C	C T	G G	C C	G G	A C	C T	A T	A G	A G	C T	A A
95	G G	C C	C C	A A	G G	G G	A A	C C	A A	G G	G G	C C	A A

96	A A	C T	C T	G G	C G	G G	A C	T T	T T	A A	A A	C T	A G
97	A A	C T	C T	G G	C C	G G	C C	C T	A T	A G	A A	C T	A A
98	A A	C C	C C	G G	C C	G G	C C	C T	A T	A G	A G	C C	A A
99	G G	C C	C T	A G	G G	G G	A A	C T	A T	A G	A G	C C	A A
100	0 0	0 0	T T	A G	C G	G G	A C	T T	T T	A A	A A	0 0	0 0
101	A G	C T	C T	A G	C G	G G	A C	C T	A T	A G	A G	C C	A A
102	A G	C T	C T	A A	G G	G G	A A	C T	A A	A G	A G	C T	A A
103	G G	C C	C C	A A	G G	C G	A C	C C	A A	G G	A G	C T	A G
104	G G	C C	C C	A G	G G	C C	C C	C C	A A	G G	A G	C T	A G
105	A G	C C	C C	A G	G G	C G	A C	C T	A T	A G	A A	C C	A A
106	G G	C C	T T	G G	G G	G G	A A	T T	T T	A A	A A	C C	A A
107	G G	C C	C C	A A	G G	G G	0 0	C C	A A	G G	G G	C C	A A
108	A A	T T	T T	A A	G G	G G	A A	T T	T T	A A	A A	C C	A A
109	G G	C C	C C	A A	G G	C C	C C	C T	A T	A G	A A	C T	A G
110	G G	C C	C C	A G	G G	C G	A C	C T	A A	A G	A A	C C	A A
111	A G	C C	C T	A G	G G	C G	A C	T T	A T	A A	A A	C C	A A
112	A A	C T	C T	G G	G G	G G	A A	C T	A A	A G	A G	C C	A G
113	A G	C T	C T	G G	C C	G G	C C	C T	A T	G G	A G	C C	A G
114	A G	C C	C T	A G	C C	G G	C C	C T	A T	A G	A A	C T	A A
115	A G	C C	C T	G G	G G	C G	A C	C T	A A	A G	A G	C C	A A
116	A G	C C	C C	A G	G G	C G	A C	T T	A T	A A	A A	C T	A A
117	A G	C T	T T	G G	C G	G G	A C	T T	A T	A A	A A	C C	A A
118	G G	C C	C T	G G	C C	G G	A C	C C	A A	G G	G G	C C	A A
119	A A	C T	C T	A G	G G	C G	C C	C T	A T	A G	A A	C C	A A
120	A G	C T	C T	A G	C G	G G	A C	T T	A T	A A	A A	C T	A A
121	A G	C T	T T	G G	C G	C G	C C	C T	A A	A G	A G	C C	A A
122	G G	C C	C T	G G	C G	G G	A C	T T	T T	A A	A A	C C	A A
123	A G	C T	C T	A A	G G	C C	C C	C T	A T	A G	A A	C T	A G
124	A A	C T	C T	G G	C G	G G	A C	T T	T T	A G	A A	C C	A A
125	A A	T T	T T	A G	G G	G G	A A	C C	A A	G G	G G	C T	A G
126	A G	C C	C C	A G	G G	G G	A A	C C	A A	G G	G G	T T	A G
127	A G	C C	C C	A A	G G	G G	A A	C T	A T	A G	A G	C C	A A
128	A G	C C	C C	A G	C G	C C	C C	T T	T T	A A	A A	C T	A A

129	G G	C C	C C	A G	0 0	G G	0 0	0 0	0 0	G G	0 0	C T	A G
130	A A	T T	T T	G G	C G	G G	A C	T T	A A	A A	A A	C T	A G
131	A G	C C	C C	A G	G G	G G	A A	C C	A A	G G	G G	C T	A A
132	G G	C C	C C	A G	C G	G G	A C	C T	A T	A G	A G	T T	A G
133	A G	C C	C C	A G	0 0	G G	0 0	0 0	0 0	A G	0 0	C C	A G
134	G G	C C	C T	G G	C G	G G	A C	C T	A T	A G	A A	C C	A A
135	A G	C C	C C	A A	G G	G G	A A	C T	A T	A G	A G	C T	A A
136	A A	C C	C C	G G	C C	G G	A C	C T	A T	A G	A G	C T	A G
137	A A	C T	C T	G G	C G	G G	A C	T T	A T	A G	A G	C C	A A
138	A G	C C	C T	G G	C G	G G	A C	T T	A T	A G	A A	C C	A A
139	A A	C T	C T	A G	C G	G G	A C	C T	A A	A G	A G	C T	A A
140	G G	C C	C C	A A	G G	C G	A C	T T	T T	A A	A A	C T	A G
141	G G	C C	C C	A G	C G	C G	C C	C T	A T	A G	A A	C C	A G
142	A G	C T	C T	G G	C C	G G	C C	C T	A T	G G	A A	C C	A A
143	A A	C T	C T	G G	C G	C G	C C	C C	A A	G G	A A	C T	A A
144	A G	C C	C C	A G	G G	C G	A C	C T	A A	A G	A G	C C	A A
145	A A	C T	C T	G G	G G	G G	A A	C T	A T	A G	A A	C T	A A
146	A G	C T	T T	G G	C G	G G	A C	T T	A T	A A	A A	C C	A A
147	A A	C C	C C	A G	C G	G G	A A	C C	A A	G G	A G	C C	A A
148	A A	C T	C T	G G	C G	C G	C C	T T	A T	A A	A A	C C	A A
149	A A	C T	C T	G G	C G	C G	A C	C T	A T	A G	A G	C T	A A
150	G G	C C	C T	A A	C G	C G	A C	C C	A A	G G	A G	C C	A A
151	G G	C C	C T	A G	C G	G G	A A	C T	A T	A G	A G	C C	A A
152	A G	C C	C C	G G	C G	C G	A C	C T	A A	A G	A G	C T	A G
153	A G	C T	C T	A G	G G	G G	A A	C C	A A	G G	G G	T T	A G
154	A G	C C	C C	A G	0 0	G G	0 0	0 0	0 0	G G	0 0	C C	A A
155	A A	C C	C C	G G	C G	G G	A C	T T	A T	A A	A A	C T	A A
156	A G	C T	T T	G G	G G	G G	A A	T T	T T	A A	A A	C T	A A
157	A G	C C	C C	A G	C G	G G	A C	C T	A T	A G	A G	C C	A A
158	A A	C T	C T	G G	C C	G G	C C	0 0	0 0	A G	0 0	C T	A G
159	A A	C C	C C	A G	C G	G G	A A	C C	A A	G G	G G	C T	A G
160	0 0	0 0	C T	A G	0 0	G G	0 0	0 0	0 0	A G	0 0	0 0	0 0
161	A A	C T	C T	G G	C G	C G	A C	T T	A T	A A	A A	C C	A A

162	A G	C C	C C	A G	C G	G G	A A	C C	A A	G G	G G	C C	A A
163	A G	C C	C T	A G	G G	C C	C C	C T	A T	A G	A A	C C	A A
164	A G	C T	C T	A G	C G	G G	A C	C T	A T	A G	A A	C C	A A
165	A A	T T	T T	G G	C G	G G	A C	T T	A T	A G	A A	C C	A A
166	A G	C T	C T	G G	C C	G G	C C	T T	A T	A A	A A	C C	A A
167	A G	C C	C T	A G	G G	G G	A A	T T	T T	A A	A A	C C	A A
168	G G	C C	C T	A G	C G	G G	A C	C T	A T	A G	A G	C C	A A
169	A A	C T	C T	A G	C C	G G	A C	C T	A T	A G	A G	C T	A A
170	A G	C C	C C	A A	G G	C G	A C	C T	A T	A G	A G	C C	A A
171	A A	C T	C T	A G	C G	G G	A C	C C	A A	G G	A A	C C	A A
172	A G	C C	0 0	0 0	0 0	0 0	0 0	0 0	0 0	0 0	0 0	C T	A G
173	A G	C T	C T	A G	G G	G G	A A	C T	A A	A G	A G	C C	A A
174	A G	C T	T T	A G	C G	C G	C C	C T	A T	A G	A G	C C	A A
175	A G	C T	T T	G G	C C	G G	C C	T T	T T	A A	A A	C T	A G
176	A A	C T	C T	A G	C G	G G	A A	C T	A T	A G	A A	C C	A A
177	A A	T T	T T	A A	G G	G G	0 0	0 0	A A	G G	G G	C T	A G
178	A A	C T	C T	G G	C G	C G	0 0	0 0	A A	A G	A G	C C	A A
179	A G	C T	C T	A G	C G	G G	0 0	0 0	A T	G G	A G	C C	A A
180	A A	C C	C C	A G	C G	C G	0 0	0 0	T T	A A	A A	C C	A A
181	A A	C T	C T	A G	G G	G G	0 0	0 0	A A	G G	G G	C C	A A
182	A G	C T	T T	G G	C G	G G	0 0	0 0	T T	A A	A A	C T	A A
183	A G	C T	C T	A G	C G	G G	0 0	0 0	A A	G G	G G	C C	A A
184	A G	C C	C C	A G	G G	C G	0 0	0 0	A A	A G	A G	C T	A G
185	A G	C C	C T	G G	C G	G G	0 0	0 0	A T	G G	A A	C T	A G
186	A A	C T	C T	A G	C G	G G	0 0	0 0	A A	A G	A G	C C	A A
187	A G	C T	C T	A G	G G	G G	0 0	0 0	A T	A G	A G	C C	A A
188	A G	C C	C C	A G	C G	G G	0 0	0 0	A A	G G	A G	C C	A A
189	A A	C C	C C	G G	C G	G G	0 0	0 0	A T	A G	A G	C C	A G
190	A G	C C	C C	A G	G G	G G	0 0	0 0	A A	G G	G G	C T	A A
191	A G	C T	C T	A G	C G	G G	0 0	0 0	A A	G G	A G	C C	A A
192	G G	C C	C T	A A	G G	C G	0 0	0 0	A T	A G	A G	T T	A G
193	A G	C T	C T	A G	G G	G G	0 0	0 0	T T	A A	A A	C C	A G
194	A G	C C	C C	A A	G G	G G	0 0	0 0	A A	G G	G G	C C	A A

195	G G	C C	C C	A G	C G	G G	0 0	0 0	A A	G G	A G	C C	A A
196	G G	C C	C C	G G	C G	G G	0 0	0 0	A A	G G	G G	C T	A G
197	A G	C C	C C	G G	C C	G G	0 0	0 0	A T	G G	A G	C T	A A
198	G G	C C	C C	A A	G G	G G	0 0	0 0	A A	G G	G G	C C	A A
199	G G	C C	C C	A A	C G	G G	0 0	0 0	A A	G G	A G	C T	A G
200	A A	C C	C C	G G	C C	G G	0 0	0 0	A T	A G	A A	C T	A G
201	A G	C C	C C	G G	G G	C G	0 0	0 0	A T	A A	A A	C T	A G
202	A A	T T	T T	A A	G G	G G	0 0	0 0	A T	A G	A G	C C	A A
203	A A	C C	C C	G G	C G	C G	0 0	0 0	A T	A A	A A	C T	A A
204	A A	C T	C T	A G	G G	C G	0 0	0 0	A A	G G	A G	C C	A A
205	A G	C T	T T	G G	G G	C G	0 0	0 0	T T	A A	A A	C T	A G
206	A A	C C	C C	G G	G G	C C	0 0	0 0	A A	A G	A A	C C	A A
207	G G	C C	C C	A G	C G	C G	0 0	0 0	A T	A G	A A	C C	A A
208	A G	C C	C C	G G	C C	G G	0 0	0 0	A T	A G	A G	C T	A A
209	A G	C C	C C	G G	C C	G G	0 0	0 0	A T	A G	A G	C T	A A
210	A A	C C	C C	G G	C G	C G	0 0	0 0	A T	A G	A A	C T	A G
211	G G	C C	T T	A A	G G	C G	0 0	0 0	A T	A G	A G	C C	A A
212	A A	C T	C T	A G	G G	C C	0 0	0 0	A T	A G	A A	C C	A A
213	A G	C T	T T	G G	G G	C G	0 0	0 0	A T	A G	A A	C C	A A
214	A A	C T	C T	A G	G G	G G	0 0	0 0	A T	A G	A G	C T	A G
215	A A	C C	C C	A A	G G	C C	0 0	0 0	A T	A G	A A	C C	A A
216	A G	C C	C T	A G	G G	G G	0 0	0 0	A A	G G	G G	C C	A A
217	A G	C T	C T	A A	G G	C G	0 0	0 0	T T	A A	A A	C C	A A
218	A A	C T	C T	A G	C G	G G	0 0	0 0	A A	A G	A G	C C	A A
219	A G	C C	C C	A G	C G	G G	0 0	0 0	A A	G G	G G	C C	A A
220	A G	C C	C C	A G	G G	G G	0 0	0 0	A A	A G	A G	C C	A A
221	A G	C T	C T	A G	C G	C G	0 0	0 0	A A	G G	A G	C T	A G
222	A A	C C	C C	G G	C G	C G	0 0	0 0	A T	A G	A A	T T	A G
223	G G	C C	C T	A A	G G	C C	0 0	0 0	T T	A A	A A	C C	A A
224	A G	C T	C T	A G	C G	C G	0 0	0 0	A T	0 0	0 0	C C	A G
225	A A	0 0	T T	G G	C G	G G	0 0	0 0	T T	A A	A A	C C	A A
226	A A	T T	T T	G G	C G	G G	0 0	0 0	A T	A A	A A	C T	G G
227	A A	C T	C T	G G	C G	C G	0 0	0 0	T T	A G	A G	C C	A A

228	A G	C C	C T	A G	C G	G G	0 0	0 0	A A	G G	A G	C C	A A
229	A G	C C	C C	A G	C C	G G	0 0	0 0	A T	A G	A G	C C	A A
230	G G	C C	C T	A G	C G	C G	0 0	0 0	T T	A A	A A	C C	A A
231	A G	C C	C C	A G	G G	C G	0 0	0 0	A A	A G	A G	C T	A A
232	A A	C C	C C	G G	G G	C C	0 0	0 0	A T	A A	A A	C C	A G
233	A A	T T	T T	G G	C G	G G	0 0	0 0	A A	A G	A G	C C	A A
234	A A	C T	C T	G G	C C	G G	0 0	0 0	T T	A A	A A	C C	A A
235	G G	C C	C T	A G	C G	C G	0 0	0 0	T T	A A	A A	C C	A A
236	A G	C T	C T	A G	C G	G G	0 0	0 0	T T	A A	A A	C C	A A
237	A A	C T	C T	G G	C C	G G	0 0	0 0	A T	A G	A A	C C	A A
238	A A	C C	C C	G G	C G	G G	0 0	0 0	T T	A A	A A	C C	A A
239	G G	C C	C T	A G	C G	G G	0 0	0 0	A T	A G	A G	C T	A G
240	A G	C C	C C	A G	C G	G G	0 0	0 0	A A	A G	A G	C C	A A
241	A G	C T	C T	A G	C G	G G	0 0	0 0	A T	A G	A G	C C	A A
242	A A	C C	C C	G G	C G	C G	0 0	0 0	A A	A A	A A	C T	A G
243	A G	C C	C C	G G	G G	G G	0 0	0 0	A A	G G	G G	C C	A A
244	A G	C T	C T	A G	C G	G G	0 0	0 0	A A	G G	A G	C T	A A
245	A G	C T	T T	G G	C G	C G	0 0	0 0	T T	A A	A A	C C	A A
246	A G	C C	C T	A G	G G	C G	0 0	0 0	A T	A G	A A	C T	A G
247	A G	C C	C C	A G	G G	C G	0 0	0 0	T T	A A	A A	C C	A A
248	A A	C T	C T	A G	C G	G G	0 0	0 0	A T	A G	A A	C C	A A
249	A G	C C	C C	G G	C G	G G	0 0	0 0	T T	A A	A A	C T	A G
250	A A	C T	C T	G G	C G	G G	0 0	0 0	A T	A G	A G	C T	A G
251	A A	C C	C C	A G	C G	G G	0 0	0 0	A T	A G	A A	C C	A A
252	A A	C C	C C	G G	C G	C G	0 0	0 0	T T	A A	A A	C T	A G
253	A G	C T	C T	A G	C C	G G	0 0	0 0	A T	A A	A A	C C	A A
254	G G	C C	C C	G G	C G	C G	0 0	0 0	A T	G G	A G	C C	A A
255	A A	T T	T T	G G	C G	G G	0 0	0 0	A T	A G	A A	C C	A A
256	A G	C T	C T	A A	G G	G G	0 0	0 0	A A	A G	A G	C C	A A
257	A G	C T	C T	G G	C G	G G	0 0	0 0	T T	A G	A A	T T	A G
258	A G	C C	C T	A G	G G	C C	0 0	0 0	A T	A G	A A	C T	A G
259	A A	C C	C C	A G	C G	C G	0 0	0 0	T T	A A	A A	T T	G G
260	A G	C T	T T	G G	C C	G G	0 0	0 0	A T	A G	A A	C T	A A

261	A A	T T	0 0	0 0	0 0	0 0	0 0	0 0	0 0	0 0	0 0	C C	A A
262	A G	C C	C C	G G	C G	G G	0 0	0 0	A T	A G	A G	C C	A A
263	A A	C C	C C	A G	C G	G G	0 0	0 0	A T	A G	A G	C C	A A
264	A G	C C	C C	A G	G G	G G	0 0	0 0	A A	A G	A G	C T	A G
265	A G	C T	C T	A G	G G	G G	0 0	C C	A A	G G	A G	C C	A A
266	A G	C C	C C	A A	G G	G G	0 0	C T	A T	A G	A G	C C	A A
267	A A	C T	C T	G G	C C	G G	C C	C T	A T	G G	A G	C C	A A
268	A A	C T	C T	G G	C C	G G	C C	C T	A T	A G	A A	C C	A G
269	A G	C C	C C	A G	C G	C G	A C	C T	A T	A G	A G	C C	A A
270	A A	C T	C T	G G	C G	G G	A C	T T	A A	A A	A A	C C	A A
271	A A	C T	C T	G G	G G	C G	A C	T T	A A	A A	A A	C C	A A
272	A G	C T	C T	A G	C G	G G	A C	C T	A T	G G	A G	C C	A A
273	A A	C T	C T	A A	G G	C G	A C	T T	T T	A A	A A	C C	A A
274	A A	C C	C C	A G	G G	C C	C C	T T	A A	A A	A A	C C	A A
275	A A	C C	C C	A A	G G	G G	0 0	C C	A A	G G	G G	C T	A A
276	A A	C C	0 0	0 0	0 0	0 0	0 0	0 0	0 0	0 0	0 0	C T	A G
277	A A	C T	C T	A G	C G	G G	A C	C C	A A	G G	A G	C C	A A
278	G G	C C	C C	A G	G G	G G	0 0	C T	A T	A G	A G	C C	A A
279	A A	C T	C T	G G	C C	G G	C C	C T	A T	A G	A G	T T	G G
280	A A	T T	T T	G G	C C	G G	A C	C T	A T	A G	A G	C C	A A
281	A G	C C	C C	A G	C G	G G	A C	C T	A T	A G	A G	C C	A A
282	A A	C T	T T	G G	G G	C G	A C	C C	A A	G G	A G	C C	A A
283	G G	C C	T T	A A	G G	C G	A C	C T	A T	A G	A G	C T	A A
284	A G	C T	C T	A G	C G	G G	A A	C C	A A	G G	A G	C C	A A

GENE	SP1	SP1	GRIN2A	GRIN2A	GRIN2A	GRIN2A	GRIN2A	HAP1	HAP1	HAP1	HAP1	E2F1	E2F1
SNP/Sample	rs2694847	rs10876450	rs8049651	rs1969060	rs8049174	rs8044807	rs11646268	rs7213337	rs11867808	rs35612698	rs4796693	rs3213183	rs3213142
1	A G	C T	C T	T T	C T	C G	C T	0 0	A A	A A	A A	C T	C C
2	A A	T T	C C	T T	C T	G G	C T	0 0	A A	A A	A A	C C	C C
3	A G	C T	C T	C T	T T	G G	C C	0 0	A G	A C	A A	C C	C C
4	A A	T T	C C	T T	C T	C G	C C	0 0	G G	C C	G G	C C	C C
5	G G	C C	C C	C T	C T	C G	C C	0 0	A G	A C	A A	C T	C C
6	A A	T T	C C	T T	T T	C G	C C	0 0	G G	C C	A G	C C	C C
7	A A	T T	C T	T T	C T	C C	C C	0 0	A G	C C	A G	C C	C C
8	A A	T T	C C	T T	C C	C G	C T	0 0	A A	A A	A A	C C	C C
9	A G	C T	C T	T T	T T	C G	C T	0 0	A G	A A	A A	C T	C C
10	A A	T T	C C	T T	C C	G G	C C	0 0	A G	A C	A A	C C	C C
11	A G	C T	C T	T T	T T	G G	C T	0 0	G G	C C	A A	C T	C C
12	A G	C T	C T	C T	T T	C G	C T	0 0	A G	A C	A A	C T	C C
13	A A	T T	C C	T T	C C	G G	C T	0 0	G G	C C	A G	C T	C C
14	A A	T T	C T	T T	T T	G G	C T	0 0	G G	C C	A A	T T	C C
15	A A	T T	C C	T T	C T	G G	C C	0 0	G G	C C	A A	C T	C C
16	A G	C T	T T	C T	C C	G G	C T	0 0	A G	A C	A A	C C	C C
17	A A	T T	C T	T T	C T	C G	C C	0 0	G G	C C	A A	C T	C C
18	A A	T T	C C	T T	C T	G G	C T	0 0	G G	C C	G G	C C	C C
19	0 0	T T	C C	C T	C T	G G	C C	0 0	G G	C C	A G	C T	C C
20	0 0	T T	C C	C T	C T	G G	C T	0 0	A G	A C	A A	C C	C C
21	A A	T T	C C	T T	C T	C G	C C	0 0	G G	C C	A A	C C	C C
22	A A	T T	C C	T T	C T	G G	C C	0 0	A A	A A	A A	C C	C C
23	A A	T T	C T	T T	T T	G G	C T	0 0	A G	A C	A G	C C	C C
24	A G	C T	C T	T T	C T	C G	C C	0 0	A G	C C	A A	C T	C C
25	A A	T T	C T	T T	C T	G G	C T	0 0	A G	A C	A A	C C	C C
26	A A	T T	C T	T T	C T	C G	C C	0 0	A G	A C	A G	C T	C C
27	A A	T T	C T	C T	T T	C G	C C	0 0	A G	A C	A A	C C	C C
28	A G	C T	C T	C T	C C	C G	C T	0 0	A G	A C	A G	C C	C C
29	G G	C C	C T	T T	T T	G G	C T	0 0	A G	A C	A A	C T	C C
30	A G	C T	C T	T T	C T	G G	T T	0 0	G G	C C	A G	C T	C C

31	0 0	T T	T T	T T	T T	C G	C T	0 0	A G	A C	0 0	C T	C C
32	0 0	T T	C T	T T	C C	G G	C C	0 0	A G	A C	A A	T T	C C
33	A A	T T	C C	C T	C C	G G	C T	0 0	G G	C C	A G	C C	C C
34	A A	T T	C T	T T	C T	G G	C C	0 0	G G	C C	A G	C T	C C
35	A A	T T	C T	C T	T T	G G	C C	0 0	A A	A A	A A	C T	C C
36	A G	C T	C T	T T	C T	C G	C C	0 0	G G	C C	A G	C T	C C
37	A G	T T	C T	C T	T T	G G	C T	0 0	G G	C C	A G	C T	C C
38	A A	T T	C C	C C	T T	C G	C T	0 0	A G	A C	A G	C T	C C
39	0 0	0 0	T T	T T	C T	C G	C T	0 0	A G	A C	A A	C T	C C
40	A G	C T	C C	T T	C T	G G	C T	CC	A A	A A	A A	T T	C C
41	0 0	0 0	C C	T T	C C	G G	C C	0 0	A G	A C	A A	C C	C C
42	A A	T T	C C	C T	T T	G G	C C	0 0	G G	C C	A G	C C	C C
43	0 0	0 0	C C	T T	T T	G G	T T	0 0	A G	A C	A A	C T	C C
44	G G	C T	C C	T T	C T	G G	C C	0 0	G G	C C	A A	T T	C C
45	G G	C C	C C	T T	C T	C G	C T	0 0	G G	C C	A A	C T	C C
46	A G	C T	C C	T T	C C	G G	C C	0 0	A G	A C	A A	C C	C C
47	A A	T T	C T	T T	T T	G G	C C	0 0	A G	A C	A G	C T	C C
48	A A	T T	C T	T T	C T	G G	C T	0 0	A G	C C	A A	C C	C C
49	A G	C T	C T	T T	C T	G G	C C	0 0	A G	A C	A G	C C	C C
50	0 0	0 0	C C	T T	T T	C G	C C	0 0	A G	A C	A G	C C	C C
51	A A	T T	C T	T T	C T	C G	C C	0 0	A G	A C	A G	C T	C C
52	0 0	0 0	C C	C T	C T	C G	C T	0 0	A G	A C	A A	C T	C C
53	A A	T T	C C	T T	C C	C G	C T	0 0	A A	A A	A A	C T	C C
54	A A	T T	T T	T T	C T	G G	C T	0 0	A G	A C	0 0	C T	C C
55	0 0	T T	T T	T T	C T	C G	C C	0 0	A A	A A	A A	C C	C C
56	0 0	T T	C C	T T	C T	C C	C C	0 0	A G	A C	A A	C T	C C
57	A G	C T	C C	T T	C T	G G	C C	0 0	A G	A C	A G	T T	C C
58	A A	T T	C T	C T	T T	G G	C C	0 0	A G	A C	0 0	C C	C C
59	A A	T T	C T	C T	C T	C G	C T	0 0	A G	A C	A A	C C	C C
60	A A	T T	C T	C T	C C	G G	C C	0 0	G G	C C	0 0	C T	C C
61	G G	C C	C T	C T	T T	G G	C T	0 0	A G	A C	A G	C T	C C
62	A A	T T	C C	C T	T T	G G	C T	0 0	G G	C C	A G	C C	C C
63	A G	C T	C C	T T	C T	C G	C C	0 0	A G	A A	A A	C C	C C

64	A G	C T	C C	T T	C T	G G	C T	0 0	A A	A A	A A	C T	C C
65	A G	C T	C T	C T	T T	G G	T T	0 0	A G	A C	A A	C T	C C
66	A A	T T	C C	T T	C T	G G	C C	0 0	A G	A C	A G	C C	C C
67	G G	C T	C T	C T	T T	G G	C T	0 0	G G	C C	A G	C T	C C
68	G G	C T	C T	C T	C T	C C	C C	0 0	G G	C C	A A	C T	C C
69	G G	C C	C C	T T	C T	C G	C T	0 0	G G	C C	A G	C C	C C
70	A A	T T	T T	T T	C C	G G	T T	0 0	A G	A C	0 0	C C	C C
71	A A	T T	C C	T T	C T	G G	C C	0 0	G G	C C	A G	C C	C C
72	A G	C T	C C	T T	C T	C G	C T	0 0	A G	A C	A A	C C	C C
73	A G	C T	T T	T T	T T	C G	C C	0 0	G G	C C	A G	C T	C C
74	A A	T T	C C	T T	T T	G G	C C	0 0	G G	C C	G G	C C	C C
75	A A	T T	T T	T T	T T	C G	C T	0 0	A A	A A	A A	C C	C C
76	A G	C T	C C	C T	C C	C G	C T	0 0	A G	A C	A G	C C	C C
77	A A	T T	C T	T T	C T	G G	C T	0 0	A A	A C	A G	C T	C C
78	A A	T T	C T	T T	C C	C C	C T	0 0	0 0	A A	A A	C C	C C
79	G G	C T	C T	T T	T T	G G	C C	0 0	A G	A C	A A	C C	C C
80	G G	C C	C C	C T	T T	C G	C C	0 0	A G	A C	A A	C T	C C
81	A G	C T	C C	T T	C T	C G	C T	0 0	A G	A C	A G	C C	C C
82	A A	T T	C C	T T	C C	C G	C T	0 0	A G	A C	A A	C T	C C
83	A A	T T	C C	T T	T T	C G	C C	0 0	A G	A C	A A	C C	C C
84	A A	T T	C C	T T	T T	G G	C C	0 0	A G	A C	A G	C C	C C
85	A A	T T	C C	T T	T T	C G	C T	0 0	A G	A C	A A	C C	C C
86	A G	C T	C T	T T	C T	C G	C T	0 0	G G	C C	A A	C T	C C
87	0 0	T T	C C	T T	T T	G G	C C	0 0	G G	A C	A A	C T	C C
88	A G	C T	C T	T T	C C	C G	C C	0 0	G G	C C	A A	C T	C C
89	A G	C T	C C	0 0	C T	G G	0 0	0 0	0 0	A C	A G	0 0	C C
90	A G	T T	C T	T T	C C	G G	C C	0 0	A G	A C	A G	T T	C C
91	A G	C T	C C	C T	T T	C G	C C	0 0	A G	A C	A A	C C	C C
92	A A	T T	C C	T T	C T	G G	C T	0 0	G G	C C	A G	C C	C C
93	A A	T T	C C	T T	T T	C G	C C	0 0	G G	C C	A G	T T	C C
94	A A	T T	C T	T T	T T	C G	C T	0 0	A G	A C	A A	C C	C C
95	A A	T T	T T	C T	C T	G G	C C	0 0	G G	C C	A A	C C	C C
96	A G	C T	C C	C T	C T	G G	C T	0 0	A A	A A	A A	C C	C C

97	A A	T T	C C	C T	C T	C G	C C	0 0	A A	A A	A A	C C	C C
98	A A	T T	C C	T T	C T	G G	C T	0 0	G G	C C	A A	T T	C C
99	A A	T T	C T	T T	C T	G G	C C	0 0	A A	A A	A A	C T	C C
100	0 0	0 0	C T	T T	T T	G G	T T	0 0	0 0	A C	0 0	0 0	C C
101	A A	T T	C T	C T	T T	G G	C C	0 0	G G	C C	A A	C T	C C
102	A A	T T	C C	T T	C T	C G	T T	0 0	A G	A C	A A	C C	C C
103	A G	C T	C C	T T	C T	0 0	C T	0 0	A A	A A	A A	C C	C C
104	A G	C T	C T	T T	C T	G G	C T	0 0	A G	A C	A A	T T	C C
105	A A	T T	C C	C T	C T	C C	T T	0 0	G G	C C	A G	T T	C C
106	A A	T T	C C	T T	C T	C G	C C	0 0	G G	C C	A G	C T	C C
107	A A	T T	C T	C C	C T	G G	C C	0 0	G G	C C	A A	C C	C C
108	A A	T T	C C	T T	C T	G G	C T	0 0	A G	A C	A A	C C	C C
109	A G	C T	C C	C T	C T	G G	C T	0 0	G G	C C	A G	C C	C C
110	A A	T T	C C	C T	C T	G G	C C	0 0	G G	C C	A A	C T	C C
111	A A	T T	C C	C T	C C	G G	C C	0 0	G G	C C	A G	C C	C C
112	A G	C T	C C	T T	T T	G G	C C	0 0	0 0	C C	0 0	C C	C C
113	A G	C T	C C	T T	T T	G G	C C	0 0	G G	C C	G G	C C	C C
114	A A	T T	C T	T T	C T	C G	C C	0 0	A G	A C	A A	C T	C C
115	A A	T T	C C	C C	C T	G G	C T	0 0	A G	A C	A A	C C	C C
116	A A	T T	C T	C T	C T	G G	C T	0 0	A G	A C	A A	C C	C C
117	A A	T T	C C	C T	T T	G G	C C	0 0	A G	A A	A A	C C	C C
118	A A	T T	C T	T T	C T	G G	C C	0 0	G G	C C	A A	C C	C C
119	A A	T T	C C	T T	T T	G G	C C	0 0	G G	C C	A G	C C	C C
120	A A	T T	C C	T T	T T	G G	C T	0 0	A G	A C	A A	C C	C C
121	A A	T T	C C	C T	T T	G G	T T	0 0	A G	A C	A G	C C	C C
122	A A	T T	C T	C T	T T	G G	C T	0 0	A G	A C	A G	C C	C C
123	A G	C T	C C	T T	C T	G G	C T	0 0	A G	A C	A A	C T	C C
124	A A	T T	T T	C T	T T	G G	T T	0 0	G G	C C	A G	C T	C C
125	A G	C T	C C	T T	C T	G G	C T	0 0	G G	C C	A A	C C	C C
126	A G	C T	C C	C T	T T	C G	C C	0 0	G G	C C	A G	C T	C C
127	A A	T T	C C	T T	T T	G G	C C	0 0	A G	C C	A G	C C	C C
128	A A	T T	C C	T T	T T	C G	C C	0 0	A A	A A	A A	T T	C C
129	A G	C T	T T	T T	T T	G G	T T	0 0	G G	C C	A A	C C	C C

130	A G	C T	C C	C C	T T	G G	C C	0 0	A G	A C	A G	C T	C C
131	A A	T T	C C	C T	T T	C G	C C	0 0	G G	C C	A A	C C	C C
132	A G	C T	C T	T T	T T	G G	C T	0 0	G G	C C	A G	C T	C C
133	A G	C T	0 0	T T	0 0	0 0	0 0	0 0	0 0	0 0	0 0	0 0	0 0
134	A A	T T	C C	T T	C T	G G	C C	0 0	A G	A C	A A	C T	C C
135	A G	T T	C T	T T	T T	G G	C C	0 0	G G	C C	A A	C T	C C
136	A G	C T	C T	C T	T T	G G	C C	0 0	A G	A C	A A	C T	C C
137	A A	T T	C C	C C	C T	G G	T T	0 0	G G	C C	A G	C T	C C
138	A A	T T	C T	C C	T T	C G	C T	0 0	A G	A C	A A	C T	C C
139	A A	T T	C C	C C	T T	G G	C T	0 0	G G	C C	A G	C T	C C
140	A G	C T	C C	T T	T T	C G	C C	0 0	G G	C C	A G	C T	C C
141	A G	C T	C C	T T	C T	C G	C C	0 0	G G	C C	A G	T T	C C
142	A A	T T	C C	T T	C T	G G	C T	0 0	G G	C C	A A	C C	C C
143	A A	T T	C T	T T	T T	G G	C T	0 0	G G	C C	A A	C C	C C
144	A A	T T	C C	T T	C T	G G	C C	0 0	A A	A A	A A	C C	C C
145	A A	T T	C C	C C	T T	G G	C T	0 0	A G	A C	A G	C C	C C
146	A A	T T	T T	C T	T T	C G	C T	0 0	A A	A A	A A	C C	C C
147	A A	T T	0 0	T T	0 0	0 0	0 0	0 0	0 0	0 0	0 0	0 0	0 0
148	A A	T T	C T	T T	C T	G G	C C	0 0	A A	A A	A A	C C	C C
149	A A	T T	T T	T T	C T	G G	C T	0 0	A G	A C	A A	C T	C C
150	A A	T T	C C	T T	C C	C G	C C	0 0	A G	A C	A A	C C	C C
151	A A	T T	C C	T T	T T	C C	C C	0 0	A A	A A	A A	C C	C C
152	A G	C T	C C	T T	C T	C G	C C	0 0	A G	A C	A A	C T	C C
153	A G	C T	T T	T T	C T	C G	C T	0 0	A G	A C	A A	C T	C C
154	A A	T T	C C	T T	T T	C G	C T	0 0	A A	A A	A A	C C	C C
155	A A	T T	C C	C T	C T	G G	C C	0 0	G G	C C	A G	C T	C C
156	A A	T T	C C	T T	C T	G G	C T	0 0	A G	A C	A A	C T	C C
157	A A	T T	C C	T T	C T	G G	C T	0 0	A A	A A	A A	C C	C C
158	A G	C T	C C	T T	C T	C G	C C	0 0	G G	C C	G G	C T	C C
159	A G	C T	C C	C T	C C	C G	C T	0 0	G G	C C	A G	C T	C C
160	0 0	0 0	C T	T T	T T	C G	C C	0 0	A G	A C	A A	C T	C C
161	A A	T T	T T	T T	C T	C G	C C	0 0	G G	C C	A A	C T	C C
162	A A	T T	C C	C T	T T	G G	C T	0 0	A G	A C	A G	C T	C C

163	A A	T T	C C	T T	T T	G G	C T	0 0	A G	A C	A A	C C	C C
164	A A	T T	C C	T T	T T	G G	C C	0 0	A G	A C	A A	C C	C C
165	A A	T T	C T	T T	C T	G G	C C	0 0	G G	C C	A G	C C	C C
166	A A	T T	C T	T T	T T	G G	C C	0 0	G G	C C	G G	C T	C C
167	A A	T T	C C	C T	C T	G G	C T	0 0	G G	C C	A G	C C	C C
168	A A	T T	C C	T T	C T	G G	C T	0 0	A G	A C	A A	C T	C C
169	A A	T T	T T	C T	T T	C G	C T	0 0	A G	A C	A A	C C	C C
170	A A	T T	C C	C T	T T	G G	C T	0 0	G G	C C	A A	C C	C C
171	A A	T T	C C	T T	T T	G G	C C	0 0	A G	A A	A A	C C	C C
172	A G	C T	C C	0 0	C T	C G	C C	0 0	A G	A C	A G	C C	C C
173	A A	T T	C C	T T	T T	G G	C T	0 0	A G	A C	A A	C T	C C
174	A A	T T	C C	T T	T T	G G	C T	0 0	G G	C C	A A	T T	C C
175	A G	C T	C T	T T	C T	G G	C C	0 0	A A	A A	A A	C C	C C
176	A A	T T	C C	T T	C T	C G	C C	0 0	A G	A C	A G	C C	C C
177	A G	C T	C T	T T	T T	G G	C T	0 0	0 0	C C	0 0	C C	0 0
178	A A	T T	C C	T T	C C	C G	C T	0 0	0 0	A A	0 0	C T	0 0
179	0 0	T T	C C	T T	C T	C G	C T	0 0	0 0	A A	0 0	C T	0 0
180	A A	T T	C T	T T	C T	G G	C C	0 0	0 0	A A	0 0	C C	0 0
181	A A	C T	C T	C T	C C	G G	C C	0 0	0 0	C C	0 0	C C	0 0
182	0 0	T T	C T	T T	T T	C G	C T	0 0	0 0	C C	0 0	C T	0 0
183	0 0	T T	C T	C T	T T	G G	C C	0 0	0 0	A C	0 0	C T	0 0
184	0 0	C T	C C	C T	C T	C G	C T	0 0	0 0	A C	0 0	C T	0 0
185	A G	C T	0 0	T T	0 0	0 0	0 0	0 0	0 0	0 0	0 0	0 0	0 0
186	A A	T T	C T	C T	C T	G G	C C	0 0	0 0	C C	0 0	T T	0 0
187	0 0	T T	C C	T T	C T	C G	C C	0 0	0 0	A C	0 0	C C	0 0
188	A A	T T	C C	C T	C T	C G	C C	0 0	A G	A A	0 0	C C	0 0
189	A G	C T	C C	T T	C T	G G	C C	0 0	0 0	A C	0 0	C T	0 0
190	A A	T T	C T	T T	T T	C G	C T	0 0	A G	A C	0 0	C T	0 0
191	A A	T T	C C	C T	T T	G G	C C	0 0	0 0	C C	0 0	C T	0 0
192	A G	C T	C C	T T	C T	C G	C C	0 0	0 0	A C	0 0	C C	0 0
193	A G	C T	C T	C T	C T	G G	C C	0 0	0 0	A C	0 0	C C	0 0
194	A A	T T	C T	T T	T T	C G	C T	0 0	A G	A C	0 0	C T	0 0
195	A A	T T	C C	C T	T T	G G	C C	0 0	0 0	A C	0 0	C C	0 0

196	A G	C T	C C	T T	C T	G G	C T	0 0	A G	A C	0 0	C C	0 0
197	A A	T T	C C	C T	C C	C G	C T	0 0	0 0	C C	0 0	C T	0 0
198	A A	T T	T T	T T	C C	G G	C C	0 0	G G	C C	0 0	C T	0 0
199	A G	C T	C C	T T	C T	G G	C T	0 0	G G	C C	0 0	C C	0 0
200	A G	C T	C T	T T	C T	C G	C C	0 0	A A	A A	0 0	C C	0 0
201	A G	C T	C T	C T	T T	G G	C C	0 0	0 0	A C	0 0	C T	0 0
202	A A	T T	C T	T T	T T	C G	C C	0 0	A G	C C	0 0	C C	0 0
203	A A	T T	C T	T T	T T	C G	C C	0 0	0 0	A C	0 0	C C	0 0
204	A A	T T	C T	C T	C C	G G	T T	0 0	0 0	C C	0 0	C C	0 0
205	A G	C T	C T	T T	C T	G G	C C	0 0	0 0	A A	0 0	C C	0 0
206	A A	T T	C T	T T	T T	G G	C C	0 0	G G	C C	0 0	C C	0 0
207	A A	T T	C C	T T	C C	C G	C T	0 0	A A	A A	0 0	C T	0 0
208	A A	T T	C T	C C	T T	G G	C C	0 0	A G	A C	0 0	C T	0 0
209	A A	T T	C T	C C	T T	G G	C C	0 0	0 0	A C	0 0	C T	0 0
210	A G	C T	C C	T T	C T	G G	C C	0 0	A G	A C	0 0	C C	0 0
211	A A	T T	C C	T T	C T	G G	C C	0 0	A G	A C	0 0	C T	0 0
212	A A	T T	C C	T T	C C	C G	C T	0 0	G G	C C	0 0	C C	0 0
213	A A	T T	C T	T T	T T	C G	C T	0 0	A G	A C	0 0	C C	0 0
214	A G	C T	C C	T T	C C	C G	C T	0 0	A G	A C	0 0	C C	0 0
215	A A	T T	C T	T T	T T	C G	C C	0 0	A A	A A	0 0	C C	0 0
216	A A	T T	C T	T T	C T	C G	C T	0 0	A A	A A	0 0	C T	0 0
217	A A	T T	C C	C T	C T	G G	C C	0 0	A A	A A	0 0	C C	0 0
218	A A	T T	C T	T T	C T	G G	C C	0 0	A G	A C	0 0	C T	0 0
219	A A	T T	C C	C T	C T	C G	C T	0 0	A A	A A	0 0	C T	0 0
220	A A	T T	C C	C T	T T	C G	C C	0 0	G G	C C	0 0	C C	0 0
221	A G	C T	C T	T T	C T	G G	C C	0 0	G G	C C	0 0	C T	0 0
222	A G	C T	C C	T T	C T	G G	C C	0 0	A G	A C	0 0	C C	0 0
223	A A	T T	C C	T T	T T	G G	C C	0 0	G G	C C	0 0	C T	0 0
224	A G	C T	C T	T T	C T	C G	C C	0 0	A G	A C	0 0	C C	0 0
225	A A	T T	C C	T T	C T	G G	C C	0 0	0 0	C C	0 0	C T	0 0
226	G G	C C	C C	T T	C C	G G	C C	0 0	0 0	C C	0 0	C C	0 0
227	A A	T T	C C	T T	C T	G G	C T	0 0	A G	A C	0 0	C T	0 0
228	A A	T T	T T	C T	T T	G G	C C	0 0	A G	A C	0 0	C C	0 0

229	A A	T T	C C	T T	C T	G G	C T	0 0	A G	A C	0 0	C T	0 0
230	A A	T T	C C	C T	T T	C G	C T	0 0	0 0	C C	0 0	C T	0 0
231	A A	T T	C T	T T	C T	G G	C C	0 0	A G	A C	0 0	T T	0 0
232	A G	C T	C T	T T	T T	G G	C T	0 0	A G	A C	0 0	C T	0 0
233	A A	T T	C T	C T	T T	G G	C C	0 0	G G	C C	0 0	T T	0 0
234	A A	T T	C C	T T	C C	C G	C T	0 0	G G	C C	0 0	T T	0 0
235	A A	T T	C T	C C	C T	G G	C C	0 0	G G	C C	0 0	C T	0 0
236	A A	T T	C C	T T	T T	G G	C C	0 0	A G	A C	0 0	C C	0 0
237	A A	T T	C T	C T	T T	G G	C C	0 0	A G	A C	0 0	C C	0 0
238	A A	T T	C T	C T	C T	G G	C C	0 0	A G	A C	0 0	C T	0 0
239	A G	C T	C T	C T	T T	G G	C C	0 0	G G	C C	0 0	C C	0 0
240	A A	T T	C T	T T	T T	G G	C T	0 0	A G	A C	0 0	C C	0 0
241	A A	T T	C C	T T	C C	G G	C C	0 0	A G	A C	0 0	C C	0 0
242	A G	C T	C T	T T	T T	G G	C T	0 0	0 0	C C	0 0	C C	0 0
243	A A	T T	C C	C T	C T	G G	C C	0 0	A G	A C	0 0	C C	0 0
244	A A	T T	C C	T T	C C	C G	C C	0 0	A A	A A	0 0	C C	0 0
245	A A	T T	C C	C C	T T	G G	C C	0 0	A G	A C	0 0	C C	0 0
246	A G	C T	C C	T T	C T	G G	C T	0 0	A G	A C	0 0	C T	0 0
247	A A	T T	C T	T T	T T	G G	C T	0 0	A A	A A	0 0	C T	0 0
248	A A	T T	C T	T T	C T	C G	T T	0 0	G G	C C	0 0	C T	0 0
249	A G	C T	C C	C T	T T	C G	C C	0 0	0 0	A A	0 0	C C	0 0
250	A G	C T	C C	C T	C T	G G	C T	0 0	A A	A C	0 0	C T	0 0
251	A A	T T	C C	T T	T T	G G	C T	0 0	A G	A C	0 0	C C	0 0
252	A G	C T	C T	T T	T T	G G	C T	0 0	G G	C C	0 0	C T	0 0
253	A A	T T	C T	C T	T T	G G	C T	0 0	A A	A A	0 0	C T	0 0
254	A A	T T	C C	T T	C C	G G	C T	0 0	A G	A C	0 0	C T	0 0
255	A A	T T	C C	C T	C C	G G	C C	0 0	A G	A C	0 0	C T	0 0
256	A A	T T	C C	T T	C C	G G	C T	0 0	A G	A C	0 0	C C	0 0
257	A G	C T	C C	C T	C T	G G	T T	0 0	0 0	C C	0 0	C C	0 0
258	A G	C T	C C	C T	T T	G G	C C	0 0	G G	C C	0 0	C T	0 0
259	G G	C C	C C	C T	T T	C C	C C	0 0	G G	C C	0 0	T T	0 0
260	A A	T T	C C	T T	C T	C G	C T	0 0	G G	C C	0 0	C T	0 0
261	A A	T T	C T	0 0	T T	C G	C T	0 0	A A	A A	0 0	C C	0 0

262	A A	T T	C T	C T	T T	C C	C C	0 0	A G	A C	0 0	C C	0 0
263	A A	T T	C C	T T	C T	G G	C C	0 0	A G	A C	0 0	T T	0 0
264	A G	C T	C C	T T	C C	G G	T T	0 0	A G	A C	0 0	C T	0 0
265	A A	T T	C C	T T	C C	G G	C C	C C	A G	A C	A A	C C	0 0
266	A A	T T	C C	C T	C T	G G	C T	C C	A G	A C	A A	C T	0 0
267	A A	T T	T T	T T	C T	C G	C C	C C	G G	C C	A A	C C	0 0
268	A G	C T	C T	T T	C C	G G	T T	C C	A G	A C	A A	C C	0 0
269	A A	T T	C C	T T	T T	G G	C T	C T	A G	A C	A G	T T	0 0
270	A A	T T	C T	C T	C T	G G	C C	C T	G G	C C	A G	C C	0 0
271	A A	T T	T T	T T	C T	C G	C T	C C	A G	A C	A A	T T	0 0
272	A A	T T	C T	T T	T T	G G	C C	C C	A G	A C	A A	C T	0 0
273	A A	T T	C T	T T	T T	G G	C C	C C	A G	A C	A A	C C	0 0
274	A A	T T	C C	T T	T T	G G	T T	C T	A G	A C	A G	C C	0 0
275	A A	T T	C C	C T	T T	C G	C T	C T	A G	A C	A A	T T	0 0
276	A G	C T	C T	0 0	C C	G G	C C	C T	G G	C C	A G	C C	0 0
277	A A	T T	C C	C T	C C	G G	C T	C T	A G	A C	A G	C C	0 0
278	A A	T T	C T	T T	C T	G G	C T	C T	A G	A C	A A	C C	0 0
279	G G	C C	C T	T T	C T	C G	C T	C T	G G	C C	G G	C T	0 0
280	A A	T T	C C	C C	C T	C G	C C	C C	A G	A C	A G	C C	0 0
281	A A	T T	C T	T T	C T	G G	C C	C C	A A	A A	A A	C C	0 0
282	A A	T T	C C	C T	T T	G G	C C	C C	G G	C C	A G	C C	0 0
283	A A	T T	C C	T T	C T	G G	C C	C C	G G	C C	A A	C C	0 0
284	A G	T T	C T	T T	C T	C G	C T	C C	A G	A C	A G	C T	0 0
